# Notch signaling activation reduces vesicular endocytosis in human pluripotent stem cell-derived CNS-like endothelial cells

**DOI:** 10.1186/s12987-025-00754-6

**Published:** 2026-01-16

**Authors:** Sarah M. Boutom, Luke D. Walsh, Maxwell M. Herman, Yunfeng Ding, Fatemeh Yaghoobi Hashjin, Benjamin K. August, Eric V. Shusta, Sean P. Palecek

**Affiliations:** 1 Department of Biomedical Engineering, University of Wisconsin - Madison, Madison, WI USA; 2 Department of Chemical and Biological Engineering, University of Wisconsin - Madison, Madison, WI USA; 3 Department of Chemistry, University of Wisconsin - Madison, Madison, WI USA; 4 Department of Biology, University of Wisconsin - Madison, Madison, WI USA; 5 Electron Microscopy Facility, University of Wisconsin – Madison, Madison, WI USA; 6 Department of Neurological Surgery, University of Wisconsin School of Medicine and Public Health, Madison, WI USA

**Keywords:** Endothelial cells, Notch signaling, Caveolae, Vesicles, Endocytosis

## Abstract

**Background:**

Mechanisms guiding the induction of blood-brain barrier (BBB) properties in central nervous system (CNS) endothelial cells during human development are incompletely understood. For example, there is a limited understanding of signaling pathways that influence the unique property of low vesicular endocytosis and transcytosis in brain microvascular endothelial cells (BMECs) relative to peripheral endothelial cells. Mouse studies suggest the importance of BBB-relevant developmental pathways, including Wnt and Notch signaling, for the induction of this BBB feature in developing BMECs.

**Methods:**

To explore induction of reduced vesicular endocytosis and transcytosis in human in vitro model of the BBB, we used human pluripotent stem cell (hPSC)-derived endothelial progenitor cells (EPCs) in which Wnt/β-catenin signaling was activated to generate hPSC-derived CNS-like ECs (hPSC-CECs). We assessed the effects of Notch signaling through overexpression of the Notch1 receptor intracellular domain (*N1ICD*).

**Results:**

*N1ICD* overexpression in hPSC-CECs resulted in upregulation of GLUT-1, a BBB-enriched glucose transporter, and decreased expression of both PLVAP and caveolin-1, two vesicular endocytosis-associated proteins. The combination of Wnt/β-catenin activation and *N1ICD* overexpression resulted in fewer vesicles and reduced albumin uptake.

**Conclusion:**

These findings indicate that Notch signaling reduces vesicular endocytosis and transcytosis in a human model of the developing BBB and contribute to our understanding of how Notch signaling induces these specific BBB properties in this model of human CNS EC development.

**Supplementary Information:**

The online version contains supplementary material available at 10.1186/s12987-025-00754-6.

## Background

Brain microvascular endothelial cells (BMECs) are specialized vascular endothelial cells that comprise most of the central nervous system (CNS) microvasculature [1, 2]. BMECs are characterized by blood-brain barrier (BBB) properties which distinguish them from similarly sized vessels in other organs due to their capability to restrict the transport of most solutes and cells from the vessel lumen into the central nervous system [3, 4]. These properties include expression of tight junction proteins such as claudin-5 and occludin, which seal cell-cell junctions and restrict paracellular transport [5, 6]. BMECs also express a battery of brain-selective nutrient and ion transporters, including members of the solute carrier (SLC) transporter family, that help maintain CNS homeostasis [7]. Polarized efflux pumps with broad-spectrum recognition of small molecules in the ATP-binding cassette (ABC) family also prevent the entry of the majority of pharmaceuticals [7–9]. Reduced vesicle-based substrate trafficking across the endothelial plasma membrane between the luminal and abluminal sides, or transcytosis, has been implicated as a crucial factor contributing to the restrictive properties of the BBB. A key property of BMECs is the greatly diminished level of endocytosis and transcytosis compared to vascular endothelial cells in peripheral organs [10, 11]. Although significantly diminished at the BBB, transcytosis can occur in CNS endothelial cells through caveolin- and clathrin-mediated pathways (smaller substrates, 50–150 nm in diameter), or through macropinocytosis (larger substrates, 200–500 nm in diameter) [12–14]. Our understanding of how this cohort of BBB properties arise in the human CNS is limited as most of our knowledge about signaling pathways and important regulators of BBB property induction and maintenance has been deduced through genetic studies in model animals, particularly mice [4]. These signaling pathways include canonical Wnt, VEGF, Notch, and TGF-β signaling, among other pathways [3, 15, 16].

Pericytes have also been implicated in contributing to reduced transcytosis at the BBB in vivo [17–19]. Of particular interest in pericyte-endothelial communication is Notch signaling, which may mediate interactions between mural and endothelial cells at the BBB [20]. An undetermined ligand on the surface of CNS pericytes is hypothesized to bind to the NOTCH1 receptor on the surface of BMECs to liberate the NOTCH1 intracellular domain (N1ICD) [20]. Upon NOTCH1 activation, the N1ICD interacts with SMAD4 which results in upregulation of N-cadherin and maintenance of pericyte-BMEC adhesions [21]. Furthermore, in an integrative single cell RNA-seq comparison of human brain and peripheral vasculature, subsequent analysis revealed that *NOTCH1* and genes encoding Notch signaling-related transcription factors *HES1/4* were enriched in brain endothelial cells compared to peripheral endothelial cells [22]. In addition, a recent study of the retinal vasculature in adult mouse models demonstrated the importance of Notch signaling, specifically the interaction between Notch ligand Dll4 and receptor Notch1, on the maintenance of low levels of vesicular transcytosis that characterize the blood-retina barrier (BRB) [23]. Interestingly, antibody-mediated blockade of the Notch ligand-receptor interaction in this context increased expression of vesicular transcytosis-associated structural proteins caveolin-1 and PLVAP, numbers of endothelial vesicles, and permeability of retinal vasculature to both 4 and 10 kDa fluorescent Dextran [23]. Furthermore, chromatin immunoprecipitation (ChIP) assays demonstrated that NOTCH signaling activation increased RBPJ-NOTCH1 binding at the *CLDN5* promoter, and lack of DLL4-NOTCH1 signaling in primary human BMECs reduced TEER and increased barrier permeability [24].

Because of the existence of Notch signaling between BMECs and brain pericytes and the molecular and functional similarities between the BRB and the BBB, we hypothesized that activation of the Notch signaling pathway in a human in vitro hPSC-derived CNS-like endothelial cell (hPSC-CECs) model would similarly affect BBB properties, especially reduction of vesicular transport. We chose the hPSC-CEC model both because of its human origin and because its activation of canonical Wnt signaling that induces some BBB properties but does not greatly reduce vesicular transport. Wnt signaling activation in these cells increases expression of BBB-enriched glucose transporter GLUT-1 (*SLC2A1*) as well as tight junctions claudin-5 (*CLDN5*) and angulin-1 (*LSR*) and reduces expression of PLVAP, a structural protein involved in vesicle-based transcytosis that is ubiquitously expressed in peripheral capillary endothelium [25]. However, Wnt signaling activation paradoxically increases transcytosis-associated structural protein caveolin-1 and yields only modest functional effects on endocytosis [25], offering a unique window through which to examine the impact of Notch signaling. To this end, we directly activated Notch signaling in hPSC-CECs by overexpressing *N1ICD*, which encodes the intracellular transcriptional effector of the NOTCH1 receptor and leads to constitutively active NOTCH1 signaling. We show *N1ICD* expression in hPSC-CECs resulted in BBB-like expression changes in MFSD2A, caveolin-1, PLVAP, and GLUT-1 as well as a functional reduction in albumin uptake that correlated with fewer caveolin-1-associated vesicles.

## Methods

### hPSC maintenance

Matrigel-coated tissue culture plates were prepared by thawing and resuspending a 2.5 mg aliquot of Matrigel, Growth Factor Reduced (Corning, Glendale, AZ) in 30 mL DMEM/F-12 (Life Technologies, Carlsbad, CA). The Matrigel solution was used to coat tissue culture plates (Corning) at a concentration of 8.7 µg/cm^2^ (1 mL/well in a 6-well plate, 0.5 mL/well in a 12-well plate). A 2.5 mg Matrigel aliquot dissolved in DMEM/F-12 is sufficient to coat up to five 6- or 12-well plates. Matrigel-coated plates were then incubated at 37 °C for a minimum of 1 h, maximum of 1 week before use. hPSCs used in this study were IMR90-4 iPSCs (WiCell, Madison, WI). hPSCs were maintained on Matrigel-coated 6-well plates in E8 medium (STEMCELL Technologies, Vancouver, Canada) at 37 °C, 5% CO_2_ with daily media changes.

hPSCs were passaged with Versene (Life Technologies) at 70–80% confluence or when colonies began to touch. To passage, hPSCs were washed once with Versene, then incubated with Versene for 7–8 min. Next, Versene was aspirated and hPSC colonies were dissociated by gently spraying with 4 mL E8 medium. To achieve a 1:12 split ratio, 2 mL of the hPSC-containing E8 suspension was transferred to a conical tube containing 4.2 mL E8. For a 1:6 split ratio, all 4 mL of the hPSC-containing E8 suspension was transferred to conical tube containing 2.2 mL E8. Next, for both split ratios, the cell suspension was gently mixed by lifting and lowering a serological pipette 5–10 times (without pipetting up and down). In a 6-well Matrigel-coated plate pre-filled with 1 mL/well E8, 1 mL/well of the mixed hPSC suspension was equally distributed, 0.5 mL per step in a U-shaped fashion, to achieve a final volume of 2 mL/well. The plate was subsequently agitated from left to right and front to back 3–4 times each and incubated at 37 °C, 5% CO_2_ for 24 h without disturbing before the next media change.

### Differentiation of hPSC-derived endothelial progenitor cells (hPSC-EPCs)

Both *N1ICD* overexpressing and unedited hPSCs were differentiated to EPCs according to previously published protocols [25–27], with minor modifications. On day-3 and D-3, hPSCs were treated with 1 mL/well Accutase (Innovative Cell Technologies, San Diego, CA) for 7 min at 37 °C. Accutase-treated hPSCs were triturated to completely singularize cells and subsequently quenched in 4x volume of E8 medium (STEMCELL Technologies). Singularized hPSCs were counted with a hemacytometer and subsequently centrifuged for 5 min, 200xg. The hPSC pellet was resuspended in E8 supplemented with 10 µM ROCK inhibitor Y-27632 (Tocris, Bristol, UK) and seeded onto Matrigel-coated 12-well plates at a density of (3–5) x 10^4^ cells/ cm^2^, 1 mL/well. Plates were incubated at 37 °C, 5% CO_2_. On each of the following 2 days (D-2 and D-1), medium was changed to E8, 1 mL/well. On D0, differentiation medium was added as LaSR medium (Advanced DMEM/F-12 [Life Technologies], 2.5 mM GlutaMAX [Life Technologies], and 60 µg/mL L-ascorbic acid 2-phosphate magnesium [Sigma-Aldrich, St. Louis, MO]) supplemented with 6 µM CHIR99021 (henceforth abbreviated as “CHIR”, Tocris), 2 mL/well. On D1, medium was changed with LaSR supplemented with 6 µM CHIR, 2 mL/well. Media changes on D1 and D2 were performed 24 h ± 30 min after the previous medium change. From D2-D4, medium was changed with pre-warmed 37 °C LaSR supplemented with 50 ng/mL recombinant human VEGF_165_ (Peprotech, Cranbury, NJ), 2 mL/well.

On D5, hPSC-EPCs were sorted by magnetic-activated cell sorting (MACS) based on CD31 surface antigen expression. hPSCs were dissociated with 1 mL/well Accutase for 15 min, 37 °C. 12 mL/plate of Accutase-treated cells were thoroughly singularized by trituration and strained through a 40 μm cell strainer (Beckton Dickinson, Vernon Hills, IL) in 38 mL DMEM (Life Technologies) supplemented with 10% FBS (R&D Systems, Minneapolis, MN). Quenched hPSCs were counted with a hemocytometer and centrifuged for 5 min, 200xg. Pelleted cells were resuspended in MACS buffer (Dulbecco’s phosphate buffer saline without Ca and Mg [DPBS; Life Technologies], 0.5% w/v bovine serum albumin [Sigma-Aldrich], 2 mM EDTA[Sigma-Aldrich]) at a concentration of 10^7^ cells/60 µL. Human FcR blocking reagent (Miltenyi Biotec, Auburn, CA) was added at a dilution of 1:50, and CD31 magnetic microbeads (Miltenyi Biotec) were added at a dilution of 20 µL/10^7^ cells, gently mixed by pipetting, incubated for 15 min, 4 °C. Cells were then washed by adding 2 mL MACS buffer/10^7^ cells and centrifuging for 5 min, 200xg. During centrifugation, a MidiMACS magnetic separator (Miltenyi Biotec) was prepared in the biological safety cabinet by placing LS columns (Miltenyi Biotec) into available slots with “fins” facing away from the magnet. The LS columns were primed with 3 mL MACS buffer. Next, the cell pellet was resuspended in 0.5-2 mL MACS buffer depending on the number of LS columns (1–4, respectively). 0.5 mL of cell suspension was loaded per LS column (about (4–6) x 10^7^ cells per column). After the cell suspension had completely flowed through the LS column, 3 additional MACS buffer washes (3 mL/column) were performed, allowing wash buffer to stop dripping completely before adding next wash fraction. After the final wash, each column was removed from the MidiMACS magnet, and cells were eluted with 5 mL MACS buffer and the provided plunger into a sterile conical tube. Eluate fractions were combined; then cells were counted by hemacytometer and centrifuged for 5 min at 200xg. A portion of the hPSC-EPCs were used for CD31/CD34 flow cytometry validation of pre-MACS and post-MACS purity (see “Flow cytometry” section of Materials & Methods). The remaining cells were either directly used for experiments (see “Lentiviral overexpression of *N1ICD* in unedited hPSC-EPCs” and “Doxycycline-induced *N1ICD* overexpression in PB-TRE-N1ICD hPSC-EPCs” sections of Materials & Methods) or cryopreserved in aliquots of 2.5 × 10^6^ cells/mL in EPC freezing medium. EPC freezing medium is 60% hECSR (human endothelial serum-free medium [hESFM; Life Technologies] supplemented with 1X B-27 supplement [Life Technologies] and 20 ng/mL FGF2 [Waisman Biomanufacturing, Madison, WI]), 30% FBS, 10% dimethyl sulfoxide (DMSO; Sigma-Aldrich) passed through a 0.22 μm pore Steriflip (Sigma-Aldrich) filter.

### Lentivirus production

Lentivirus plasmids pWPI (Figure S1A, Addgene plasmid #12254), psPAX2 (Addgene plasmid #12260), and pMD2.G (Addgene plasmid #12259) were obtained from Addgene (Watertown, MA) as gifts from Didier Trono. To generate transfer plasmid pWPI-N1ICD (Figure S1B), a construct encoding the native human Notch1 intracellular domain (N1ICD) was cloned into the pWPI plasmid after the EF-1α promoter and ahead of the IRES cassette. First, a cDNA fragment encoding the intracellular domain of the Notch1 receptor was amplified from a cDNA library generated from an hPSC-derived neural crest cells [28]. This fragment spans nucleotides 5,522–7,930 of NM_017617.5, corresponding to amino acids 1,754–2,556 of NP060087.3. For N1ICD, the forward primer (Table S2) contained a Kozak consensus sequence and start codon; forward and reverse primers (Table S2) included PacI restriction enzyme sites. pWPI and the resulting PCR fragments were digested with PacI. Ligation was performed with Instant Sticky-end Ligase Master Mix (New England Biolabs, Ipswich, MA), and the resulting products were transformed into NEB Stable Competent *E. coli* (New England Biolabs). Single ampicillin-resistant colonies were picked and screened via PCR for presence of insert using primers annealing to the EF-1α promoter and IRES (Table S2). Clones with forward-oriented inserts were identified and the correct sequence was confirmed via Sanger Sequencing. pWPI and pWPI-N1ICD plasmids were then expanded and purified using the EndoFree Plasmid Maxi Kit (Qiagen, Germantown, MD). pWPI-N1ICD has been deposited to Addgene (Addgene plasmid #185525).

Lentivirus encoding *N1ICD*-IRES-*GFP* (denoted N1ICD LV) and IRES-*GFP* negative control (denoted GFP LV) constructs were produced in HEK293TN cells (System Biosciences, Palo Alto, CA). 293TN cells were maintained on uncoated 6-well tissue culture plates in DMEM (Life Technologies) supplemented with 10% FBS (R&D Systems, Minneapolis, MN), 1 mM sodium pyruvate (Life Technologies), and 0.5x GlutaMAX Supplement (Life Technologies), with media changes every other day. When 293TN cells reached 70–90% confluence, they were transfected with packaging plasmids psPAX2 (1 µg/well) and pMD2.G (0.5 µg/well), and transfer plasmid pWPI-N1ICD or pWPI (1.5 µg/well) using FuGENE HD Transfection Reagent (Promega, Madison, WI). Medium was supplemented with 1X antibiotic-antimycotic (Life Technologies) on the day of transfection. Approximately 16–18 h later, medium was aspirated and replaced with fresh pre-warmed HEK293TN media, and virus-containing supernatants were collected 24, 48, and 72 h later. After 1st and 2nd collections, medium was replaced with fresh pre-warmed 293TN medium and virus-containing supernatant was cooled at 4 °C. After the final collection, the 3rd virus-containing supernatant fraction was cooled at 4 °C for a minimum of 30 min to overnight. The three virus-containing supernatants were then combined, centrifuged to remove cell debris, and passed through a 0.45 μm pore Steriflip filter (Millipore SE1M003M00) before being concentrated 100X with Lenti-X Concentrator (Takara Bio, Mountain View, CA). Briefly, Lenti-X/media mixture was incubated at 4 °C for 30 min before centrifuging at 1,500xg & 4 °C for 45 min. Subsequently, supernatant was aspirated, and lentivirus-containing pellets were resuspended in 200 µL each of sterile DPBS and subsequently combined to form a single consistent stock. 25–50 µL aliquots were prepared and frozen at -80 °C for long-term storage.

### hPSC-EPC culture

Collagen IV (Sigma-Aldrich) was dissolved in 0.5 mg/mL acetic acid to a final concentration of 1 mg/mL. Collagen IV-coated plates were made by diluting a volume of the 1 mg/mL stock solution 1:100 in sterile water and adding the resulting solution to tissue culture plates. 1 mL collagen IV solution/well was added to 6-well plates (9.5 cm^2^/well) and the volume of solution was scaled according to surface area in other multi-well plate formats (e.g., 100 µL/well for a 48-well plate [0.95 cm^2^/well]). After adding collagen IV solution, the tissue culture plates were incubated for 1 h at RT, protected from light. Collagen IV coating solution was then removed, and cryopreserved hPSC-EPCs were thawed and resuspended in hECSR medium and plated at approximately 4 × 10^4^ cells/cm^2^. hPSC-EPCs were incubated at 37 °C, 5% CO_2_ with hECSR medium changes every other day. In some experiments, small molecules and/or lentivirus were added to hECSR medium: in all cases, 4 µM CHIR (Tocris) was added to the culture medium to activate Wnt/β-catenin signaling. To activate Notch signaling, we overexpressed *N1ICD* using lentiviral transduction in unedited hPSC-derived EPCs or by addition of doxycycline in EPCs derived from hPSCs with tetracycline-inducible expression of *N1ICD*. Details describing the lentiviral and drug-inducible approaches for *N1ICD* overexpression are discussed in the subsequent sections.

### HUVEC culture

Human umbilical vein endothelial cells (HUVECs) pooled in EGM-2 (Lonza, Basel, Switzerland) were thawed and plated at a seeding density of 2500 cells/cm^2^ on an uncoated T-75 flask. Cells were incubated at 37 °C, 5% CO_2_ and medium was replaced every other day with EGM-2 medium (EBM-2 medium [Lonza] supplemented with EGM-2 Endothelial SingleQuots Kit [Lonza]). When cells reached 80–90% confluency, cells were incubated with ~ 5 mL 0.25% Trypsin-EDTA, with phenol red (Life Technologies) for 10 min at 37 °C and dissociated by trituration. Trypsinized HUVECs were quenched in 4x the volume of EGM-2 medium and centrifuged at 200xg for 5 min. The cell pellet was resuspended in EGM-2 to a concentration that would achieve a seeding density of 2500 cells/cm^2^ in an uncoated 6-well plate. Cells were cultured for 2 days before replacing EGM-2 medium supplemented with 1.9 µL/well GFP LV or 50 µL/well N1ICD LV. Medium was changed every other day with EGM-2 medium. 6 days after lentiviral transduction of HUVECs, cells were dissociated and stained with CD31 or caveolin-1 antibodies for characterization by flow cytometry (see “Flow cytometry” section of Materials & Methods for a complete description).

To study substrate accumulation in HUVECs, cells were plated at seeding density of 5000 cells/cm^2^ in each well of two 12-well plates with 1 additional well in a third 12-well plate. Cells were incubated at 37 °C, 5% CO_2_ and medium was replaced every other day with EGM-2 medium. Cells were cultured for 2 days before replacing EGM-2 medium supplemented with 0.76 µL/well GFP LV or 20 µL/well N1ICD LV (doses of both types of lentivirus scaled down by a factor of 2.5x from 6-well plate). Medium was changed every other day with EGM-2 medium. 6 days after lentiviral transduction of HUVECs, cells were pretreated with or without endocytosis inhibitors, incubated with or without fluorescent albumin, imaged by epifluorescence microscopy, and isolated for flow cytometry analysis (see “Fluorescent albumin accumulation assay” section of Materials & Methods for a complete description).

### Lentiviral overexpression of *N1ICD* in unedited hPSC-EPCs

Unedited hPSC-derived EPCs (D5) with high post-MACS purity, assessed by flow cytometry, were cultured on collagen IV-coated plates at approximately 4 × 10^4^ cells/cm^2^ in hECSR medium supplemented with 4 µM CHIR (Tocris). For characterization by flow cytometry, RT-qPCR, or Western blotting, EPCs were seeded in 6-well plates. For characterization by immunocytochemistry, EPCs were seeded in 48-well plates. Medium was changed every other day until D11. On D7, EPCs were transduced with 5.26 µL/cm^2^ N1ICD LV (50 µL/well in a 6-well plate, 5 µL/well in a 48-well plate) or 0.66 µL/cm^2^ GFP LV negative control (6.25 µL/well in a 6-well plate, 0.625 µL/well in a 48-well plate). On D11, cells were isolated for various downstream assays described in the sections below. Flow cytometry was used to determine expression of GFP, CD31, and caveolin-1 or PLVAP (Table S1). Gene expression was assayed by RT-qPCR (Table S2). Western blotting was used to quantify expression of Notch1 (including full length protein and intracellular domain [N1ICD]) and β-actin (Table S1). Cells were stained for GFP, caveolin-1, PLVAP, GLUT-1, and PECAM-1 and imaged with immunofluorescence microscopy (Table S1).

### Generation of doxycycline-inducible *N1ICD*-overexpressing hPSCs

*N1ICD* and preceding Kozak sequence were PCR amplified using Q5 High-Fidelity 2X Master Mix (New England Biolabs) from pWPI-N1ICD with primers containing NheI and AgeI restriction site overhangs (Table S2). The amplification product was run on a 1% agarose gel. The band corresponding to the *N1ICD* amplicon with NheI and AgeI overhangs was cut out of the gel and DNA was purified using the Monarch DNA Gel Extraction Kit (New England Biolabs). Plasmid PB-TRE-ETV2 was digested using NheI-HF and AgeI-HF (New England Biolabs) to separate the *ETV2* cassette from the plasmid backbone containing a doxycycline-inducible TRE3G promoter, an EF-1α core promoter followed by a Tet-On 3G cassette, a hygromycin resistance cassette, and 3’ and 5’ piggyBac inverted repeats. The backbone also contained an ampicillin resistance cassette outside the piggyBac inverted repeats for cloning purposes. The restriction digest fragments of PB-TRE-ETV2 were separated by gel electrophoresis on a 1% agarose gel into ~ 8 kb backbone and ~ 1 kb *ETV2* insert. The band corresponding to the backbone was excised and DNA was purified using the Monarch DNA Gel Extraction Kit (New England Biolabs). Additionally, the *N1ICD* amplicon with NheI and AgeI overhangs was similarly digested with NheI-HF and AgeI-HF to create sticky ends. The digested *N1ICD* amplicon with restriction overhangs was column washed using the DNA Clean and Concentrator-5 kit (Zymo Research, Irvine, CA). Sticky-end ligation of the *N1ICD* PCR product with restriction overhangs (~ 2.5 kb) and PB-TRE-ETV2 plasmid backbone (~ 8 kb) was performed with the T4 DNA Ligase (Thermo Scientific, Waltham, MA). The resulting ~ 10.5 kb plasmid (PB-TRE-N1ICD) (Figure S6A) was transformed into NEB Stable Competent *E. coli* (New England Biolabs). Single ampicillin-resistant colonies were picked, expanded, and plasmid DNA was purified with the ZymoPURE Plasmid Miniprep Kit (Zymo Research). The resulting PB-TRE-N1ICD plasmids were digested with NheI-HF and AgeI-HF (New England Biolabs), and the resulting restriction fragments were run on a 1% agarose gel to verify presence of an 8 kb backbone, 2.5 kb insert (*N1ICD*) and absence of a 1 kb insert (*ETV2*) (Figure S6C). Clones with appropriate restriction fragments were Sanger sequenced with 10 sequencing primers (Figure S6B, Table S2) to verify that the *N1ICD* sequenced matched that in pWPI-N1ICD.

Unedited IMR90-4 hPSCs were reverse-transfected with a Super PiggyBac Transposase Expression Vector (PB210PA-1 [System Biosciences]) and the doxycycline-inducible *N1ICD* overexpressing transposon plasmid (PB-TRE-N1ICD) using the *Trans*IT-LT1 Transfection Reagent (Mirus Bio, Madison, WI) according to the manufacturer’s protocol. Briefly, 0.5 mL mTeSR1 medium (STEMCELL Technologies) was added to a well in a Matrigel-coated 6-well plate. In a sterile tube, 400 µL Opti-MEM (Life Technologies) was supplemented with 12 µL *Trans*IT*-*LT1 Transfection Reagent and 4 µg plasmid DNA (5:2 ratio of PB-TRE-N1ICD [2857.1 ng] to PB210 [1142.9 ng]), mixed and incubated at room temperature for 15 min. During incubation, hPSCs were singularized for 10 min with Accutase (Innovative Cell Technologies), quenched in 4x volume of mTeSR1 medium and centrifuged for 5 min at 200xg. During the centrifugation, the plasmid and transfection reagent mixture was added to the well containing mTeSR1 medium and incubated at room temperature for the remainder of the procedure. Accutase-treated hPSCs were counted and resuspended in mTeSR1 medium to a final concentration of 2 × 10^6^ cells/mL. Cells were transferred to the well plate containing *Trans*IT-LT1:DNA complexes. The medium was supplemented with 10 µM ROCK inhibitor Y-27632 (Tocris) and 1X Antibiotic-Antimycotic (Life Technologies), and cells were incubated at 37 °C, 5% CO_2_. 24 h later, medium was replaced with mTeSR1 medium. Transfected hPSCs were cultured with daily mTeSR1 medium changes until 60–70% confluent, then cells were passaged to a new Matrigel-coated plate. After the cells reached ~ 20% confluency, media were replaced with mTeSR Plus medium (STEMCELL Technologies) supplemented with 50 µg/mL hygromycin B in PBS (Invitrogen, Carlsbad, CA). Medium was replaced daily with mTeSR Plus medium supplemented with 50 µg/mL hygromycin. If cell density appeared to decline on consecutive days, hygromycin was omitted and mTeSR Plus was supplemented with 10 µM Y-27632. Cells were passaged with Versene in mTeSR Plus medium without hygromycin at a split ratio of 1:6. Hygromycin selection of transfected hPSCs was performed for a total of three passages, after which 70–80% confluent wells of the heterogeneous population of PB-TRE-N1ICD hPSCs were cryopreserved in hPSC mTeSR1 freezing medium (60% mTeSR1, 30% FBS, 10% DMSO). *N1ICD* and *HEYL* expression in heterogeneous PB-TRE-N1ICD hPSCs treated with 1 µg/mL doxycycline hyclate (Thermo Scientific) in sterile DPBS or 1:1000 diluted sterile DPBS was determined by RT-qPCR.

Next, we selected PB-TRE-N1ICD hPSC clones with specific copy numbers of the integrated PB-TRE-N1ICD transposon. Three 10 cm Matrigel-coated cell culture dishes (6 mL DMEM/F-12 supplemented with Matrigel [see “hPSC maintenance” for details] added to each ~ 57 cm^2^ dish) were prepared and incubated at 37 °C, 5% CO_2_ for a minimum of 1 h before use. One well of ~ 70% confluent IMR90-4 PB-TRE-N1ICD hPSCs was dissociated with Accutase for 9 min and quenched through a 40 μm cell strainer (Beckton Dickinson) in 4x volume of mTeSR Plus. Cell counts were quantified by hemacytometer. 2 × 10^2^, 2 × 10^3^, and 1 × 10^4^ cells were separately suspended in 8 mL each of mTeSR Plus supplemented with 1X CloneR2 (STEMCELL Technologies), added to the Matrigel-coated dishes, agitated, and incubated at 37 °C, 5% CO_2_. Medium was changed daily with mTeSR Plus supplemented with 1X CloneR2 until colonies became visible. Afterwards, medium was changed daily with mTeSR Plus. After ~ 7 days, when colonies had grown but were not touching, 12 colonies from 2 × 10^3^ cells/dish and 11 colonies from 2 × 10^2^ cells/dish were picked. Briefly, Matrigel-coated 12-well plates were pre-filled with 1 mL/well mTeSR Plus supplemented with 1X Antibiotic-Antimycotic. Additionally, plates receiving colonies picked from 2 × 10^3^ cells/dish or 2 × 10^2^ cells/dish were supplemented with 1X CloneR2 or 10 µM Y-27632, respectively. Individual colonies were visualized with the EVOS XL Core Imaging System (Life Technologies), manually dislodged and aspirated with a p200 pipette tip, transferred into a separate well in the Matrigel-coated 12-well plate, and incubated at 37 °C, 5% CO_2_. Medium was changed daily with mTeSR Plus supplemented with 1X CloneR2 or 10 µM Y-27632 until colonies had attached and begun to proliferate. Medium was changed daily with mTeSR Plus. When the center of a picked colony became opaque under a brightfield microscope or increased cell death was observed, the colony was passaged with Versene to one well in a Matrigel-coated 6-well plate in mTeSR Plus supplemented with 10 µM Y-27632. Subsequent Versene passages were performed without addition of 10 µM Y-27632. In total, 11 clonal hPSC populations were isolated, gDNA was extracted using the Monarch Genomic DNA Purification Kit (New England Biolabs), and PB-TRE-N1ICD transposon copy number in each clone was quantified using PiggyBac qPCR Copy Number Kit (System Biosciences). Cells were cryopreserved in hPSC mTeSR1 freezing medium. Four clones with variable transposon copy numbers were thawed and cultured in E8 medium supplemented with 1:1000 diluted sterile DPBS or different doxycycline concentrations (1 µg/mL, 200 ng/mL, and 100 ng/mL), and *NOTCH1* expression was quantified by RT-qPCR (Figure S7B). All 4 clonal PB-TRE-N1ICD hPSC lines (3[+ Y], 9[+ Y], 10[+ CR2], and 11[+ CR2]) were sent for G-banded karyotyping (WiCell, Madison, WI) and determined to have normal karyotypes.

### Doxycycline-induced *N1ICD* overexpression in PB-TRE-N1ICD hPSC-EPCs

PB-TRE-N1ICD hPSC-derived EPCs (D5) with high post-MACS purity validated by flow cytometry were cultured on collagen IV-coated plates at approximately 4 × 10^4^ cells/cm^2^ in hECSR medium supplemented with antibiotics, with media changes every other day. We investigated the optimal timing for expression of *N1ICD* in hPSC-EPCs by culturing heterogeneous *N1ICD*-expressing hPSC-EPCs with 4 or 6 days of hygromycin reselection followed by 4 or 6 days of doxycycline. The condition with highest percentage of CD31 + cells (~ 90%) and highest CD31 mean fluorescence intensity (MFI) determined by flow cytometry was 6 days of hygromycin reselection followed by 4 days of doxycycline treatment. We also determined that hygromycin reselection was not necessary for *N1ICD* overexpression or related BBB gene expression. Following optimization of the timing of doxycycline treatment, we used the following scheme. From D5 until D11, hECSR medium was supplemented with 4 µM CHIR (Tocris) and 1:1000 DPBS. From D11 until D15, hECSR medium was supplemented with 4 µM CHIR and either 1:1000 DPBS or 1 µg/mL doxycycline hyclate (Thermo Scientific) in DPBS. On D15, cells were collected for RT-qPCR quantification of differential gene expression between doxycycline and DPBS-treated PB-TRE-N1ICD hPSC-EPCs.

### Immunocytochemistry

Cells were washed once with DPBS and fixed with ice-cold methanol for 15 min. Cells were washed once with DPBS and blocked in DPBS supplemented with 10% goat serum (Life Technologies) for 1 h at room temperature on a rocking platform. Cells were washed once with DPBS. Primary antibodies (Table S1) diluted in DPBS supplemented with 10% goat serum were added to cells and incubated at 4 °C overnight (18–24 h) on a rocking platform. The following day, cells were washed 2 times with DPBS. Secondary antibodies (Table S1) diluted in DPBS supplemented with 10% goat serum were added to cells and incubated for 1 h at room temperature on a rocking platform, protected from light. Cells were washed once with DPBS and incubated for 5 min in DPBS supplemented with 4 µM Hoechst 33342 (Life Technologies). Images were acquired using an Eclipse Ti2-E epifluorescence microscope (Nikon, Tokyo, Japan) with a 10x or 20x objective.

Images were analyzed using FIJI (https://fiji.sc/). Marker fluorescence intensity was determined as follows: mean background fluorescence in each channel was quantified by measuring the average mean gray value (MGV) of a cell-free field in 3 different image fields. Marker fluorescence intensity for an image field containing cells was normalized to the number of nuclei (indicated by Hoechst counterstain) by calculating the difference between MGV for marker of interest and the mean background MGV in the associated channel; this value was divided by the difference between the cell-containing field MGV and mean background MGV in the Hoechst channel.

### Flow cytometry

To assess expression of CD31 and CD34 in differentiated hPSC-EPCs, flow cytometry samples were prepared on D5 of differentiation. hPSC-EPCs were incubated for 15 min at 37 °C, 5% CO_2_ with Accutase, triturated, and filtered through a 40 μm cell strainer into 4x the volume of DMEM (Life Technologies) supplemented with 10% FBS (R&D Systems). Cell count was quantified using a hemocytometer and cells were centrifuged at 200xg for 5 mins. Cells were resuspended in MACS buffer at a concentration of 100 µL/10^6^ cells. Two aliquots of 10^6^ cells each were aliquoted pre-MACS and kept at 4 °C during MACS. (4–5) x 10^5^ cells were aliquoted post-MACS. Pre- and post-MACS cell suspensions were supplemented with CD31-APC and CD34-FITC antibodies or corresponding APC and FITC isotype control antibodies (Table S1) at a 1:50 dilution and incubated at 4 °C for 20 min, protected from light. Cells were washed with 2 mL MACS buffer and centrifuged at 200xg for 5 min. Pellets were resuspended in 500 µL each of 4% paraformaldehyde (PFA, Electron Microscopy Sciences, Hatfield, PA) in DPBS and incubated at room temperature for 15 min, protected from light. Cells were centrifuged at 200xg for 5 min and pellets were resuspended in 400 µL MACS buffer. Samples were analyzed on an Attune NxT V6 flow cytometer (Thermo Fisher) with excitation at 488 nm and 633 nm. FITC emission was detected with a 530/30 filter and APC emission was detected with a 670/14 filter. Data were analyzed with Flow Jo software (BD Biosciences, San Jose, CA).

To determine transduction efficiency of GFP and N1ICD lentiviruses and measure CD31 expression in transduced unedited hPSC-CECs (Figure S2C, D), cells were isolated on D11 to prepare flow cytometry samples. Cells were initially prepared in a similar fashion to the hPSC-EPCs described above. Pellets of transduced cells were resuspended in MACS buffer (100 µL/10^6^ cells) supplemented with CD31-APC or APC isotype control antibodies at a 1:50 dilution. Pellets of untransduced cells were resuspended in MACS buffer supplemented with CD31-APC. Cells were incubated at 4 °C for 20 min, protected from light. Cells were washed with 2 mL MACS buffer and centrifuged at 200xg for 5 min. Pellets were resuspended in 500 µL each of 1% PFA in DPBS and incubated 15 min at room temperature, protected from light. Cells were centrifuged at 200xg for 5 min and pellets were resuspended in 400 µL MACS buffer. Samples were analyzed on an Attune NxT V6 flow cytometer with excitation at 488 nm and 633 nm. GFP emission was detected with a 530/30 filter and APC emission was detected with a 670/14 filter. Data were analyzed with Flow Jo software.

To determine caveolin-1 and CD31 expression in GFP LV or N1ICD LV transduced hPSC-CECs or HUVECs (Figure S3, S4), hPSC-CECs were isolated at D11 and HUVECs were isolated at 6 days following transduction, respectively. Samples also included unstained and fluorescence minus one (FMO) controls. Cells were dissociated with either Accutase or Trypsin-EDTA, respectively, as previously stated, strained, and resuspended in MACS buffer (100 µL/10^6^ cells). Cell suspensions were supplemented with 1:50 diluted CD31-APC and incubated at 4 °C for 20 min, protected from light. Cells were washed with 2 mL MACS buffer and centrifuged at 200xg for 5 min. Pellets were resuspended in 500 µL each of 4% PFA in DPBS and incubated 15 min at room temperature, protected from light. Cells were centrifuged at 200xg for 5 min, and pellets were resuspended in 500 µL 0.1% Triton X-100 (Sigma Aldrich) in MACS buffer and incubated 30 min at room temperature, protected from light. Cells were washed with 2 mL MACS buffer and centrifuged at 200xg for 5 min. Cells were then resuspended in 400 µL MACS buffer supplemented with 1:50 diluted caveolin-1-PE antibody (Table S1) and incubated at 4 °C overnight on a rocking platform, protected from light. The following day, cells were washed twice with 2 mL MACS buffer and centrifuged at 200xg for 5 min. Cells were resuspended in 400 µL MACS buffer. Samples were analyzed on an Attune NxT V6 flow cytometer with excitation at 488 nm, 561 nm, and 633 nm. GFP emission was detected with a 530/30 filter, PE emission was detected with a 585/16 filter, and APC emission was detected with a 670/14 filter. Data were analyzed with Flow Jo software.

### Fluorescence-activated cell sorting (FACS)

PB-TRE-N1ICD hPSC-EPCs were cultured in a 6-well plate with or without doxycycline as described in the “Doxycycline-induced *N1ICD* overexpression in PB-TRE-N1ICD hPSC-EPCs” section of Materials & Methods. On D15, hPSC-CECs treated with or without doxycycline were isolated with Accutase (incubated 15 min at 37 °C, 1 mL/well), triturated, and filtered through a 40 μm cell strainer into 4x the volume of DMEM (Life Technologies) supplemented with 10% FBS (R&D Systems). An additional well of *N1ICD* overexpressing EPCs cultured with PBS was reserved as an isotype control. Unedited hPSCs maintained in E8 medium and unedited hPSC-EPCs cultured for 6 days in hECSR supplemented with 4 µM CHIR were used as an additional negative and positive control, respectively, for FACS. Cell count was quantified using a hemocytometer and cells were centrifuged at 200xg for 5 min. Cells were resuspended in MACS buffer at a concentration of 100 µL/10^6^ cells. Cell suspensions were supplemented with CD144-APC or APC-conjugated isotype control antibodies (Table S1) at a 1:50 dilution for at 4 °C for 20 min, protected from light. Cells were washed with 2 mL MACS buffer and centrifuged at 200xg for 5 min. Pellets were resuspended in 400 µL each of MACS buffer supplemented with 1 µg/mL DAPI (Invitrogen) and incubated at 4 °C, protected from light.

Cells were sorted using a BD FACSAria III Cell Sorter (BD Biosciences). Gates for high or low CD144-expressing cells were determined based on two negative controls (PB-TRE-N1ICD hPSC-derived EPCs stained with APC isotype control antibody or unedited hPSCs stained with CD144-APC antibody, both negative for CD144) and one positive control (unedited hPSC-EPCs cultured with CHIR, CD144 high). After establishing gates, live PB-TRE-N1ICD hPSC-CECs treated with or without doxycycline were sorted into tubes containing 500 µL MACS buffer and stored at 4 °C until RNA isolation.

### RT-qPCR

RNA extraction was performed using the Direct-zol RNA Miniprep Kit (Zymo Research). Cells were lysed with 300–600 µL TRIzol reagent (Invitrogen, Waltham, MA) depending on the estimated cell count per sample. Cell lysates were combined with an equal volume of 100% ethanol and transferred to Zymo-Spin IICR spin columns. Columns were incubated with RNase-free DNase I (Qiagen) to eliminate residual gDNA. Columns were washed with RNA Wash Buffer and Direct-zol RNA Pre-Wash according to Zymo RNA Miniprep Kit protocols. RNA was eluted with RNase-free water and concentration was determined using a NanoDrop 2000 spectrophotometer (Thermo Scientific). 250–1000 ng of RNA was reverse transcribed for 1 h at 37 °C using the OmniScript RT Kit (Qiagen). 1 U/µL RNaseOUT (Life Technologies) was included in the reverse-transcription reactions. Reaction products were diluted to 10 ng/µL. 20 µL qPCR reactions were carried out with 10 ng cDNA and 500 nM each forward and reverse primers (Integrated DNA Technologies, Coralville, IA [Table S2]) using PowerUp SYBR Green Master Mix (Life Technologies) or 1X Taqman Gene Expression Assay probes (Thermo Scientific [Table S2]) using Taqman Fast Advanced Master Mix (Thermo Scientific). Reactions were run on an AriaMx Real-Time PCR System (Agilent Technologies, Santa Clara, CA) using the thermal cycling program corresponding to either IDT primers or Taqman probes. An annealing temperature of 60 °C was used for all reactions.

### Western blotting

Cells were lysed with radioimmunoprecipitation assay (RIPA) buffer (Rockland Immunochemicals, Pottstown, PA), supplemented with Pierce Protease and Phosphatase Inhibitor (Thermo Scientific) and centrifuged at 4 °C for 5 min, 16,000xg. Supernatants were collected, transferred to new tubes, and stored at -80 °C. Protein concentration was quantified using the Pierce BCA Protein Assay Kit. For each sample, 6.5 µg of protein was diluted to equal volume with water, mixed with sample buffer, and heated to 95 °C for 5 min. Samples were then run on 4–12% Tris-glycine gels and transferred to nitrocellulose membranes. Membranes were blocked for 1 h in tris-buffered saline with 0.1% Tween-20 (TBST) supplemented with 5% non-fat dry milk. Primary antibodies (Table S1) were diluted in TBST supplemented with 5% non-fat dry milk, added to membranes, and incubated overnight (16–24 h) at 4 °C on a rocking platform. Membranes were washed 5 times with TBST. Secondary antibodies (Table S1) were diluted in TBST supplemented with 5% non-fat dry milk, added to membranes, and incubated for 1 h at room temperature on a rocking platform, protected from light. Membranes were washed 5 times with TBST and imaged using an Odyssey 9120 (LI-COR, Lincoln, NE). The same membrane was blotted for both Notch1 and β-actin; however, the membrane was initially blotted only for Notch1 and following imaging, the membrane was re-blotted for β-actin. Band intensities were quantified using Image Studio software (LI-COR).

### Fluorescent albumin accumulation assay

Fixable, Alexa Fluor 647-conjugated bovine serum albumin (BSA) (Invitrogen) was used as a substrate to quantify total fluid-phase endocytosis. D15 cultures of PB-TRE-N1ICD hPSC-CECs treated with 4 µM CHIR with or without 1 µg/mL doxycycline were pre-treated for 30 min at 37 °C with or without various endocytic pathway inhibitors (3 mM methyl-β-cyclodextrin [MβCD, Sigma-Aldrich], 20 µM chlorpromazine [CPZ, Sigma-Aldrich], or 2 µM rottlerin [Tocris]). Similarly, D6 cultures of HUVECs treated with GFP or N1ICD LV were pre-treated with or without 3 mM MβCD for 30 min at 37 °C. Cells were washed once with sterile DPBS and then incubated in hECSR (for hPSC-CECs) supplemented with 5 µg/mL BSA or 1:1000 diluted DPBS, 1:100 diluted CD144-FITC or FITC isotype control antibody, and 4 µM Hoechst 33342 (Life Technologies) or EGM-2 media (for HUVECs) supplemented with 5 µg/mL BSA or 1:1000 diluted DPBS and 4 µM Hoechst 33342 at 4 °C or 37 °C for 2 h on a rotating platform at 30 rpm, protected from light. Cells were then washed 3 times with 4 °C sterile DPBS to stop the assay and wash away any extracellular BSA. Internalization of fluorescent BSA was visualized using an Eclipse Ti2-E epifluorescence microscope (Nikon, Tokyo, Japan) with a 20x objective.

Flow cytometry was used to quantify accumulated BSA fluorescence within cells. Individual well replicates were dissociated with Accutase (for edited 10(+ CR2) hPSC-CECs, 1 mL/well for 15 min @ 37 °C) or 0.25% Trypsin-EDTA (for HUVECs, 0.5 mL/well for 10 min @ 37 °C), quenched in hECSR (hPSC-CECs) or EGM-2 (HUVECs) through a 40 μm cell strainer and centrifuged. Pellets were resuspended in 500 µL each of 4% PFA in DPBS and incubated 15 min at room temperature, protected from light. Cells were centrifuged at 200xg for 5 min and pellets were resuspended in 400 µL MACS buffer. For 3(+ Y), 9(+ Y), and 11(+ CR2) edited hPSC-CECs, cells dissociated with 300 µL/well of Accutase and quenched directly into 1.2 mL hECSR without straining in a 96-well V-bottom deep well plate (Corning). The plate was centrifuged at 300xg for 5 min @ 4 °C, and pellets were resuspended in 400 µL MACS buffer without a prior fixation step. All samples were analyzed on an Attune NxT V6 flow cytometer with excitation at 488 nm and/or at 633 nm. FITC emission was detected with a 530/30 filter and AlexaFluor 647 emission was detected with a 670/14 filter. Data were analyzed with Flow Jo software.

### Caveolin-1 Immunogold labeling and transmission electron microscopy

PB-TRE-N1ICD hPSC-EPCs were cultured on sterile 15 mm round cover glasses (CELLTREAT Scientific Products, Pepperell, MA) coated with 10 µg/mL collagen-IV within a 12-well tissue culture plate (Corning). Cells were seeded at 4 × 10^4^ cells/cm^2^ in hECSR supplemented with 4 µM CHIR, 1:1000 diluted DPBS, and 1X antibiotic-antimycotic (Life Technologies). Cells were cultured with media changes every other day until D11. On D11, medium was changed with hECSR supplemented with 4 µM CHIR, 1 µg/mL doxycycline or 1:1000 diluted DPBS, and 1X antibiotic-antimycotic. This medium was changed every other day until D15.

On D15, cells were washed once with DPBS. *N* = 4 wells each treated with doxycycline or DPBS were fixed with 4% PFA (Electron Microscopy Sciences) + 0.1% glutaraldehyde (GA, Electron Microscopy Sciences) in 0.1 M Sorensen’s phosphate buffer (PB) (for caveolin-1 immunogold labeling) or 2% PFA + 2.5% GA in 0.1 M PB (for ultrastructural imaging) for 15 min at room temperature. Cells were washed with DPBS. Cells fixed with 2% PFA + 2.5% GA were stored at 4 °C in DPBS until proceeding with downstream processing for TEM.

Cells fixed with 4% PFA + 0.1% GA (for caveolin-1 immunogold labeling) were permeabilized with 0.1% saponin from Quillaja bark (Sigma-Aldrich) in DPBS for 10 min at room temperature. Cells were washed with DPBS and blocked in 10% goat serum in DPBS for 1 h, rocking at room temperature. Cells were washed with DPBS and incubated overnight rocking at 4 °C in 10% goat serum supplemented with α-caveolin-1 primary antibody (Table S1). Cells were washed twice with DPBS, and incubated rocking at 4 °C in 10% goat serum supplemented with Nanogold-conjugated secondary antibody (Table S1, Nanoprobes, Yaphank, NY). Cells were washed in DPBS. Cells were post-fixed with 1% GA in phosphate buffer for 30 min at room temperature. The HQ Silver kit (Nanoprobes) was used to silver enhance samples for 7 min at room temperature, protected from light. All samples (both ultrastructure imaging and caveolin-1 immunogold staining) were embedded in epoxy resin and sectioned parallel to the cell culture surface with a microtome before mounting on TEM grids. Sample sections were subsequently treated with osmium tetroxide (Electron Microscopy Sciences) for 20 min. Samples for ultrastructure imaging were contrast stained with uranyl acetate for 15 min followed by lead citrate for 10 min. Samples with caveolin-1 immunogold labeling were contrasted with a lead citrate and uranyl acetate solution for 30 s. Grids were imaged using a FEI CM120 transmission electron microscope (Philips, Amsterdam, Netherlands). Images were analyzed using FIJI by a researcher who was blinded to the treatment condition. Number of vesicles, vesicle diameter, and total cell area were measured manually, while Nanogold area was determined via thresholding.

### Transendothelial electrical resistance (TEER)

Transwell inserts (6.5 mm diameter with 0.4 μm pore polyester membrane) (Corning) were coated with 50 µl of a collagen-IV (400 µg/mL) and fibronectin (100 µg/mL) solution in sterile water and incubated at 37 °C for a minimum of 4 h before seeding cells. PB-TRE-N1ICD hPSC-EPCs (clone 10[+ CR2]) were seeded on D5 at a density of 10^5^ cells/cm^2^ onto the Transwell membrane in hECSR medium supplemented with 4 µM CHIR and 1:1000 diluted DPBS. Medium volumes were 100 µL for the apical chamber and 600 µL for the basolateral chamber. Medium was replaced every other day. Starting the day after seeding, TEER was measured daily for 10 days with an EVOM2 epithelial voltohmeter with STX2 chopstick electrodes (World Precision Instruments, Sarasota, FL). TEER values were calculated by subtracting the resistance of a collagen-IV/fibronectin-coated Transwell insert without cells and multiplying by the Transwell surface area (0.33 cm^2^).

### Bulk RNA-sequencing and analysis

Ten PBS or doxycycline-treated PB-TRE-N1ICD hPSC-derived endothelial cell subpopulations obtained via FACS were centrifuged at 200xg for 5 min. Pellets were resuspended TRIzol reagent (Invitrogen) for extraction of total RNA, similar to the procedure described in “RT-qPCR” section of Material & Methods. RNA concentrations were quantified using the Qubit 4 Fluorometer (Invitrogen) and Qubit RNA High Sensitivity Assay Kit (Invitrogen). Purified RNA samples (≥ 200 ng) were sent to Novogene Corporation (Sacramento, CA) for library preparation and mRNA sequencing, with approximately 40–60 million paired end reads obtained per sample. FASTQ files were upload to the Galaxy [29] web platform and processed to generate a raw counts matrix using the publicly available bioinformatics server at usegalaxy.org. FastQC was run to ensure high Phred quality scores and low percentages (< 5%) adapter content (Galaxy). Illumina Universal Adapter sequences were trimmed from raw sequencing reads using Cutadapt (Galaxy). Data were aligned to the hg38 genome model using RNA STAR and raw reads per gene were determined using featureCounts. Differential expression analysis was performed in R using DESeq2 [30]. Heatmaps containing TPM values were generated using the pheatmap package in R. Comparison of the transcriptomic profile of *N1ICD* overexpressing CECs ± Dox subpopulations to transcriptomes of established cell and tissue types was performed with PACNet [31].

### Analysis of published scRNA-seq data

Three publicly available single cell RNA-sequencing (scRNA-seq) data sets [22, 32, 33] were analyzed using the Seurat [34] package in RStudio. We isolated endothelial cells from an integrative analysis of multiple in vivo human vascular single cell RNA-seq datasets [22], and performed differential expression analysis between brain endothelial cells and peripheral organ (i.e., heart, liver, lung, and skeletal muscle) endothelial cells. We identified a list of statistically significantly differentially expressed genes (false discovery rate < 0.05) which included transcription factors and other regulatory factors as well as blood-brain barrier marker genes.

To compare PB-TRE-N1ICD hPSC-CECs treated with CHIR with or without doxycycline to in vivo brain microvascular endothelial cells, we obtained embryonic [32] and adult [33] brain vascular scRNA-seq datasets. Within each data set, we subsetted Seurat objects corresponding to capillary endothelial cells based on metadata provided by the authors. In order to make these single cell datasets comparable to the bulk RNA-seq in vitro datasets, we pseudo-bulked the single cell transcriptomes for each gestational time point except GW20 (very few capillary ECs) in the Crouch et al. [32] dataset and all healthy control adult samples from the Yang et al. data set [33].

### Statistics

Statistics for most experiments were performed using Microsoft Excel or GraphPad Prism software. Student’s *t*-test was used to compare differences of means between 2 groups. One-way ANOVA with post-hoc Tukey’s or Dunnet’s test was used to compare differences of means between 3 or more groups. For bulk RNA-sequencing analysis, p-values were calculated using the DESeq2 Wald test with Benjamini-Hochberg correction.

## Results

### Characterization of endocytosis and transcytosis-associated markers in hPSC-CECs overexpressing *N1ICD*

**Fig. 1:**
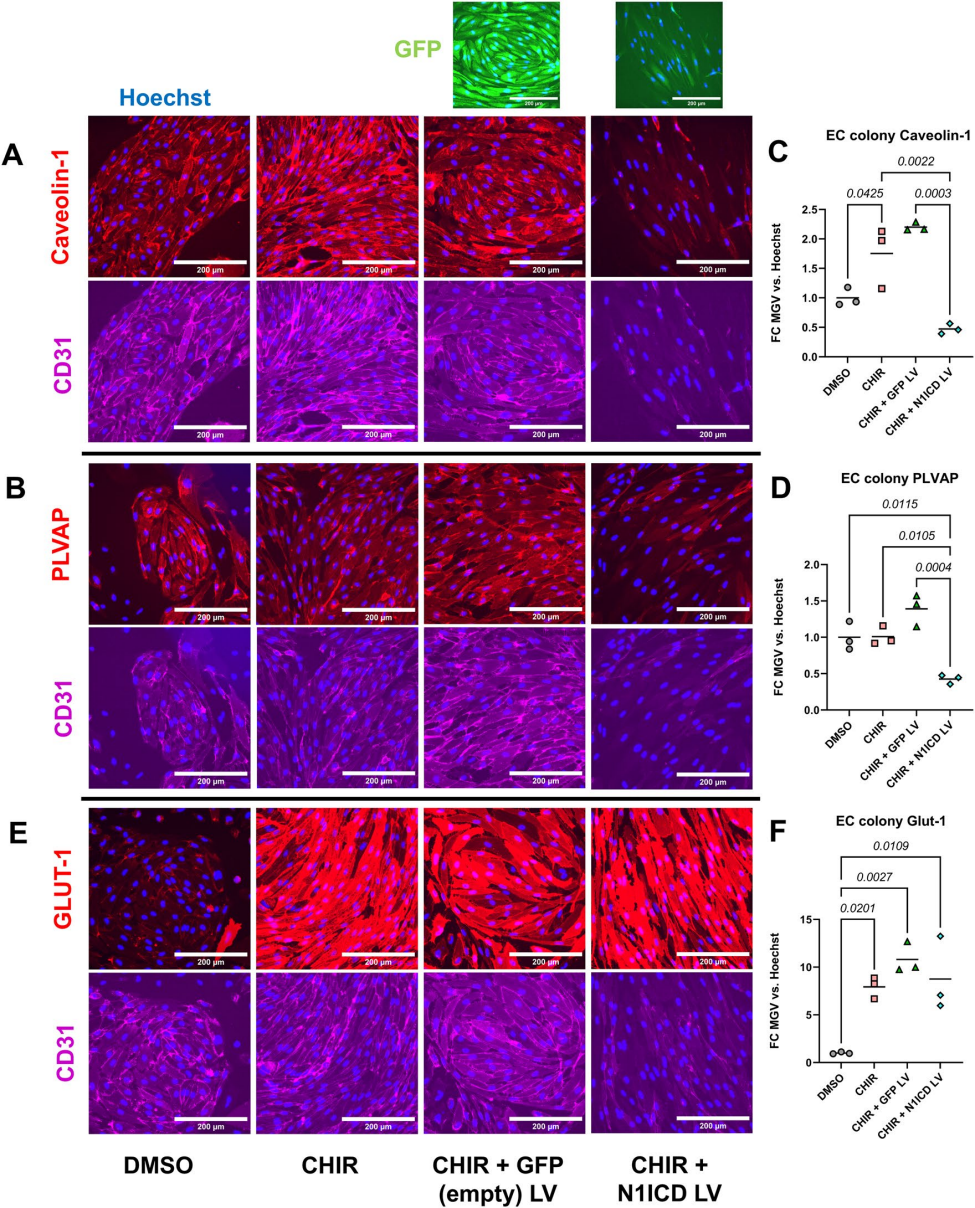
Immunostaining of unedited hPSC-EPCs and hPSC-CECs with lentiviral constitutive *N1ICD* overexpression: Immunocytochemistry (ICC) analysis of various markers in hPSC-EPCs treated with DMSO, CHIR (hPSC-CECs), CHIR and GFP LV, or CHIR and N1ICD LV on D11 after 6 days of culture in hECSR supplemented with DMSO or CHIR and 4 days following transduction with either GFP or N1ICD LV. **(A)** Representative images of ICC analysis for caveolin-1, CD31, and GFP in various treatment conditions. Only LV transduced cells expressed GFP. Hoechst nuclear counterstain is overlaid in all images. Scale bar: 200 μm. **(B)** Quantification of caveolin-1 mean gray value (MGV) in different conditions from (A), normalized to Hoechst MGV. **(C)** Representative images of ICC analysis for PLVAP and CD31 in indicated treatment conditions. GFP staining not shown. Hoechst nuclear counterstain overlaid in all images. Scale bar: 200 μm. **(D)** Quantification of PLVAP MGV in indicated conditions from (C), normalized to Hoechst MGV. **(E)** Representative images of ICC analysis for GLUT-1 and CD31 in indicated treatment conditions. Hoechst nuclear counterstain overlaid in all images. Scale bar: 200 μm. **(F)** Quantification of GLUT-1 MGV in indicated conditions from (C), normalized to Hoechst MGV. In **(B)**, **(D)**, and **(F)**, points represent *n =* 3 biological replicates from one differentiation of IMR90-4 iPSC-derived EPCs. Horizontal bars indicate mean. Hoechst-normalized relative fluorescence for each of the three markers was further normalized within each analysis such that the mean of the DMSO condition was equal to 1. Statistical analyses were performed on Hoechst-normalized data; P-values: One-way ANOVA followed by Tukey’s HSD test

To determine the effects of activated Notch signaling in hPSC-CECs, hPSC-derived EPCs were differentiated from hPSCs as previously described [25] (Figure S2A) and subsequently treated with CHIR to produce hPSC-derived CNS-like ECs (hPSC-CECs). Notch1 signaling was activated through *N1ICD* overexpression achieved by lentiviral transduction. On the second day of treatment of hPSC-derived EPCs with CHIR (day 7 overall, Figure S2A), developing hPSC-CECs were transduced with N1ICD or GFP (empty control) encoding lentivirus (Figure S2B). Lentivirus dosage and transduction were optimized for both N1ICD LV and GFP LV to achieve a transduction efficiency of at least 85–95%. Resultant cell populations were ~ 100% positive for the endothelial marker CD31, although there was a reduction in CD31 expression in N1ICD-transduced cells that correlated with increased transgene expression (e.g. increased GFP MFI in N1ICD transduced samples, Figure S2C). We next measured expression and localization of vesicular transport-associated proteins caveolin-1 [35] and PLVAP [36] by immunostaining since BMECs have reduced expression of these two proteins compared to peripheral ECs [37]. hPSC-EPCs that were treated with both 4 µM CHIR (hPSC-CECs) and transduced with N1ICD LV had significantly lower expression of caveolin-1 and PLVAP than those treated with CHIR alone or treated with CHIR and GFP LV (Fig. 1A, C, B and D). We also confirmed by flow cytometry that hPSC-EPCs treated with CHIR and N1ICD LV had significantly reduced caveolin-1 expression vs. CHIR and GFP LV-treated hPSC-EPCs (Figure S3A, C). Additionally, given that CHIR leads to strong upregulation of the BBB-enriched glucose-transporter GLUT-1 relative to the vehicle in hPSC-CECs [25], we confirmed that significant upregulation in GLUT-1 expression was maintained in hPSC-EPCs treated with CHIR and transduced with N1ICD LV relative to vehicle (Fig. 1E, F). Because our previous study demonstrated that these earlier passage, ”immature” hPSC-EPCs were more responsive to CHIR-mediated GLUT-1 upregulation [25], we tested whether N1ICD LV could also mediate reduction in caveolin-1 in human umbilical vein ECs (HUVECs). We observed a significant reduction in caveolin-1 expression in HUVECs treated with CHIR and N1ICD LV (Figure S3B, D), demonstrating that this combination of Wnt and Notch activation could also reduce expression of transcytosis genes in mature ECs lacking any in vivo BBB properties. In addition, although hPSC-CECs treated with CHIR and N1ICD LV retained expression of CD31, we observed reduced CD31 fluorescence intensity in immunostained cells (Fig. 1) and confirmed this finding in both hPSC-CECs and HUVECs transduced with N1ICD LV by flow cytometry (Figure S4). We then measured expression of several BBB-related genes via RT-qPCR in hPSC-CECs treated with N1ICD LV. We observed a significant reduction in *CAV1* and *PLVAP* expression (~ 2-fold) and significant upregulation of *MFSD2A* expression (~ 5-fold) in hPSC-CECs transduced with N1ICD LV compared to control (Figure S5A-C). Mouse in vivo studies demonstrated that lipid flippase MFSD2A, which regulates plasma membrane lipid composition and is enriched in CNS endothelial cells relative to peripheral endothelium, is associated with fewer caveolae and reduced transcytosis [10, 11]. An additional study suggested that canonical Wnt signaling is involved in an axis that upregulates MFSD2A and reduces caveolin-1-associated vesicles, resulting in reduced transcytosis at the blood-retina barrier (BRB) [38]. There was also no significant change in expression of tight junction-related genes *CLDN5*,* OCLN*, or *TJP1* (Figure S5D-F) or efflux-transporter genes *ABCB1*,* ABCG2*, or *ABCC1* (Figure S5G-I). A ~ 2.5-fold increase was observed in *SLC2A1* expression in hPSC-CECs treated with CHIR + N1ICD LV compared to Wnt signaling activation alone (CHIR + GFP LV) (Figure S5J). Overall, these data indicate that the primary effect of treatment of hPSC-CECs with CHIR and N1ICD LV is a reduction in expression of vesicular transcytosis-related genes, whereas other key BBB axes related to physical barrier and efflux transporter properties remain unchanged at the transcript level.

### Generation of hPSC lines with doxycycline-inducible overexpression of *N1ICD*

**Fig. 2:**
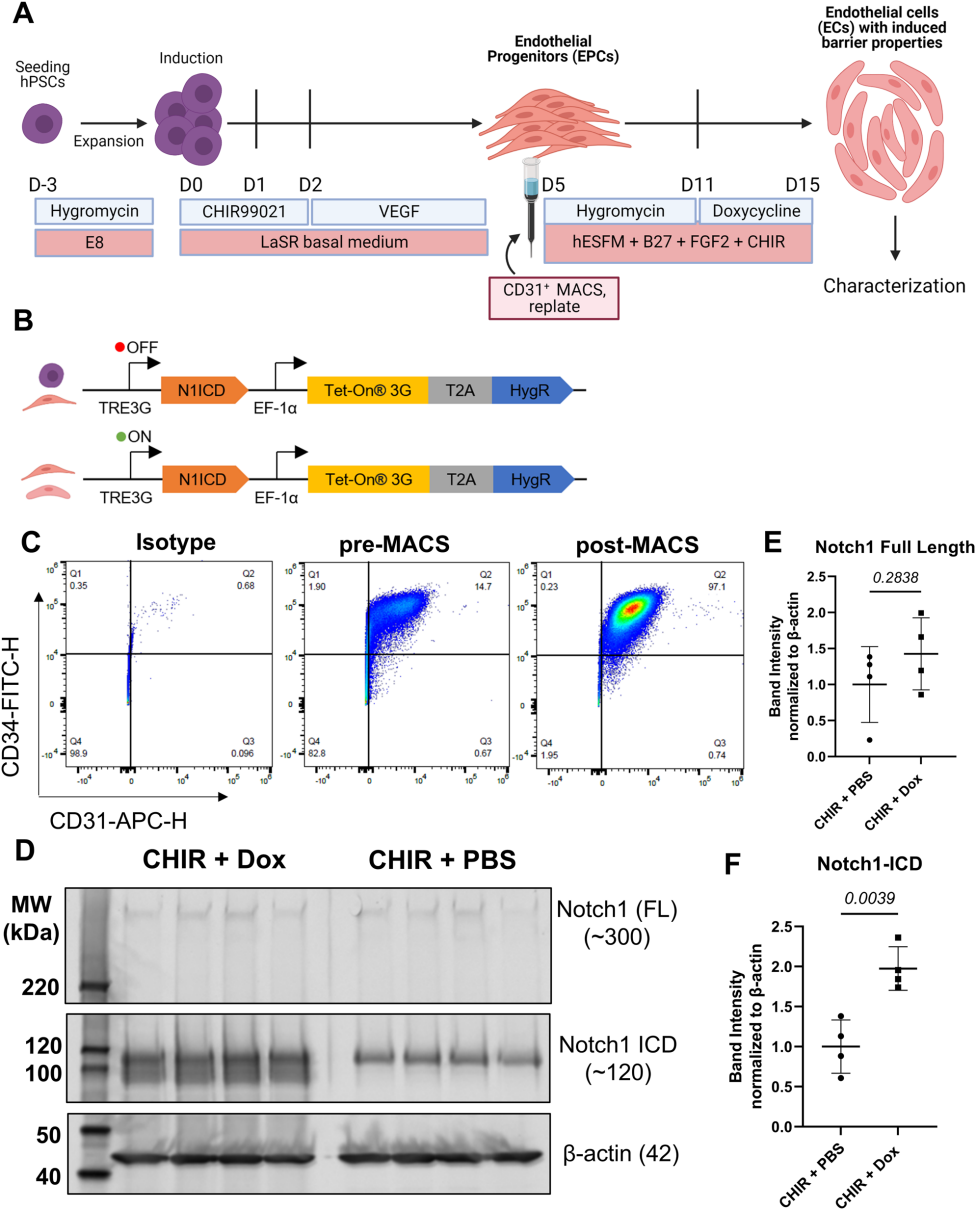
Differentiation and validation of PB-TRE-N1ICD hPSC-CECs with Dox-inducible N1ICD overexpression: **(A)** Protocol for differentiation of hPSCs to EPCs, followed by MACS sorting, hygromycin reselection, CHIR treatment to derive CECs and doxycycline treatment to overexpress *N1ICD*. **(B)** Schematic of the doxycycline inducible *N1ICD* overexpressing transposon construct. The N1ICD cassette follows a TRE3G doxycycline inducible promoter. A cassette encoding the Tet-On 3G protein follows an EF-1α core promoter, resulting in constitutive expression of this gene. Tet-On 3G must associate with doxycycline to bind to the TRE3G inducible promoter. Also following the EF-1α promoter and Tet-On 3G are self-cleaving 2A peptide linker (T2A) followed by a hygromycin resistance gene (HygR). The top portion of the schematic indicates that during differentiation of hPSCs to CECs, the TRE3G promoter is not active due to the absence of doxycycline. In the bottom portion, hPSC-CECs that have been differentiated and sorted can be treated with doxycycline to activate *N1ICD* overexpression. **(C)** Representative flow cytometry plots of D5 hPSC-EPCs derived from a population of *N1ICD* overexpressing IMR90-4 iPSCs with heterogeneous copy numbers of the integrated construct shown in (B). Graphs show percentage of CD31+/CD34 + EPCs before (pre-MACS) and after (post-MACS) sorting. **(D)** Western blotting analysis of D15 hPSC-derived PB-TRE-N1ICD 10(+ CR2) clonal EPCs treated with CHIR (hPSC-CECs), with or without doxycycline. Membranes blotted for Notch1 full length (N1 FL) protein and intracellular domain (N1ICD), as well as β-actin. Predicted approximate molecular weights of each detected protein are indicated on the right-hand side. **(E)** N1 FL and **(F)** N1ICD, normalized to respective input control (β-actin) band intensities. In all Western blot analyses, points represent *n* = 4 biological replicates from one differentiation of IMR90-4 PB-TRE-N1ICD 10(+ CR2) iPSC-derived CECs. Bars indicate mean ± SD. β-actin-normalized band intensities were further normalized within each analysis such that the mean of the CHIR + PBS condition was equal to 1. Statistical analyses were performed on β-actin-normalized data; P-values: Student’s *t*-test

When we performed immunoblotting for Notch1 protein in CHIR + GFP LV and CHIR + N1ICD LV-treated hPSC-CECs (Figure S2E), there was no difference in full-length Notch1 expression. In addition, while we detected Notch1 ICD in cells that had been transduced with N1ICD LV, we did not observe statistically significant changes (Figure S2F, G).This is likely due variability in number of cells transduced and number of copies of the *N1ICD* overexpression construct per cell, resulting in different N1ICD expression population-level averages in each sample. To circumvent this issue and to achieve more precise control over the gene dosage and duration of *N1ICD* overexpression, we generated an hPSC line with doxycycline-inducible overexpression of *N1ICD* to achieve specific, reproducible levels of overexpression by optimizing the doxycycline dose and timing. The line was generated using a piggyBac transposon vector (PB-TRE-N1ICD) containing a doxycycline-inducible *N1ICD* overexpression cassette (Figure S6A-C, Fig. 2B). After hygromycin selection of hPSCs with integration of the PB-TRE-N1ICD transposon, a heterogeneous pool of transfected hPSCs treated with doxycycline showed a significant increase in expression of *N1ICD* as well as *HEYL*, a gene regulated downstream of Notch signaling (Figure S6D, E). When the population of doxycycline-inducible *N1ICD* overexpressing hPSCs was differentiated to EPCs (Fig. 2A), it had similar purity both before (~ 15%) and after (~ 100%) CD31-selective MACS to that observed when differentiating unedited hPSC-EPCs (Fig. 2C) [26, 39]. We also demonstrated increased N1ICD expression in hPSC-CECs treated with CHIR and Dox by immunoblotting, which contrasted with the situation using the mixed population lentiviral overexpression approach (Fig. 2D).

We next selected individual clones of PB-TRE-N1ICD hPSCs having specific copy numbers of the doxycycline-inducible *N1ICD* overexpressing transposon to examine the effects of gene dosage on *N1ICD* overexpression. We selected a total of 11 clones with piggyBac transposon copy numbers ranging from ~ 2 to ~ 48 copies (Figure S7A). Four of these clones with a range of copy numbers (~ 2 [9(+ Y)], ~ 15 [11(+ CR2)], ~ 24 [10(+ CR2)], and ~ 48 [3(+ Y)] copies) were selected for further characterization. We treated these clones at the undifferentiated hPSC stage with a range of doxycycline concentrations to determine if a doxycycline dose or gene copy number correlated with *N1ICD* overexpression. Although there was a consistent increase in *N1ICD* expression for all 4 clones with increasing concentrations of doxycycline, the expression of *N1ICD* at similar doxycycline doses did not correlate with copy number of the PB-TRE-N1ICD transposon in each of the hPSC clones (Figure S7B). This finding is likely attributable to variability in genomic location of piggyBac transposon integration and thus variability in accessibility of the transposon to transcriptional machinery and other local gene regulatory elements.

### Characterization of clonal N1ICD-expressing hPSC-CECs

With the N1ICD-expressing hPSC clones in hand, we next investigated the effects of N1ICD overexpression on hPSC-CECs. We compared expression of selected BBB-related genes in hPSC-CECs derived from doxycycline-inducible *N1ICD* overexpressing clones 3(+ Y), 9(+ Y), 10(+ CR2), and 11(+ CR2). The *N1ICD* overexpressing hPSC-CECs were treated with doxycycline ranging from 100 ng/mL to 1 µg/mL or PBS controls from D11-D15 of differentiation (Fig. 2A). Significant, dose-dependent increases in *NOTCH1* and *MFSD2A* expression were observed across all 4 clones (Figure S8A, B). The highest degrees of *NOTCH1* and *MFSD2A* upregulation were observed in CECs derived from clones 10(+ CR2) and 11(+ CR2), which had intermediate copy numbers amongst the 4 clones analyzed. A dose-dependent decrease in *CAV1* expression (Figure S8C) was seen in all 4 clonal edited hPSC-CECs. Similarly, a decrease in *PLVAP* was seen with increasing doxycycline dose in all 4 lines except 9(+ Y) hPSC-CECs, which demonstrated peak reduction in *PLVAP* expression at the lowest doxycycline concentration (1:10K, 100 ng/mL), followed by an increase to just below the baseline (+ PBS) expression level at the higher dox concentrations (1:5K [200 ng/mL], 1:1K [1 µg/mL]) (Figure S8D). We also observed a general trend of a dose-dependent increase in *SLC2A1* for all 4 lines except 3(+ Y) (Figure S8E). Among the 4 lines tested, the greatest reduction in *CDH5* expression was observed in CECs derived from 10(+ CR2) and 11(+ CR2) edited hPSCs, correlating with the high degree of *N1ICD* overexpression in these two clones with increasing doxycycline dosage (Figure S8F). There was a ~ 2-fold decrease in *CDH5* expression at the highest doxycycline dose for the 3(+ Y) hPSC-CECs and a non-significant decrease for the 9(+ Y) hPSC-CECs. Taken together, these data suggest that upregulation of *MFSD2A* and *SLC2A1* and downregulation of *CAV1* and *PLVAP* are dependent on the degree *N1ICD* overexpression. The results also indicate that there is a trade-off between more BBB-like expression of genes related to vesicular transcytosis and selective nutrient transport with that of canonical endothelial marker expression levels.

**Fig. 3:**
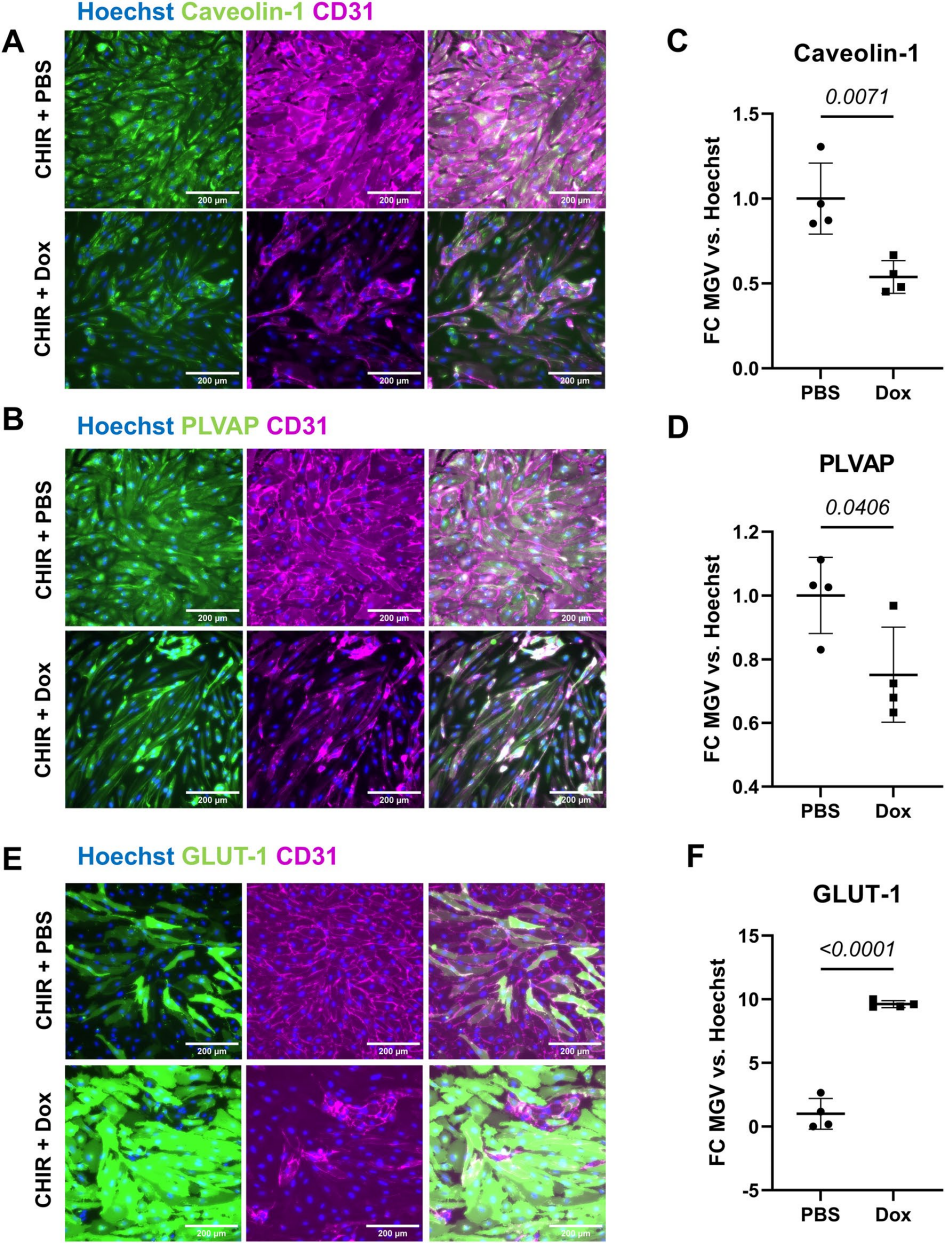
Immunostaining of PB-TRE-N1ICD hPSC-CECs: Immunocytochemistry (ICC) analysis of indicated markers in PB-TRE-N1ICD hPSC-CECs on D15 after 10 days of culture in hECSR supplemented with CHIR and 1:1000 diluted DPBS from D5 to D11 and 4 days of CHIR and doxycycline (1 µg/mL) or PBS treatment from D11 to D15. **(A)** Representative images of ICC analysis for caveolin-1 and CD31. Hoechst nuclear counterstain is overlaid in all images. Scale bar: 200 μm. **(B)** Quantification of caveolin-1 mean gray value (MGV) in different conditions from (**A**), normalized to Hoechst MGV. **(C)** Representative images of ICC analysis for PLVAP and CD31. Hoechst nuclear counterstain is overlaid in all images. Scale bar: 200 μm. **(D)** Quantification of PLVAP MGV in different conditions from (C), normalized to Hoechst MGV. **(E)** Representative images of ICC analysis for GLUT-1 and CD31. Hoechst nuclear counterstain is overlaid in all images. Scale bar: 200 μm. **(F)** Quantification of GLUT-1 MGV in different conditions from (**C**), normalized to Hoechst MGV. In (**B**), (**D**), and (**F**), points represent *n =* 4 biological replicates from one differentiation of IMR90-4 PB-TRE-N1ICD 10(+ CR2) hPSC-CECs. Horizontal bars indicate mean ± SD. Hoechst-normalized relative fluorescence for each of the three markers was further normalized within each analysis such that the mean of the PBS condition was equal to 1. Statistical analyses were performed on Hoechst-normalized data; P-values: Student’s *t* test

Given the large extent of *N1ICD* and BBB-associated gene induction observed with clone 10(+CR2), we used this clone to examine the effects of *N1ICD* overexpression on BBB-related protein expression after 1 µg/mL (1:1K) doxycycline or PBS control treatment of 10(+ CR2) hPSC-CECs for 4 days. We confirmed a significant decrease in caveolin-1 (Fig. 3A, B) and PLVAP (Fig. 3C, D), and an increase in GLUT-1 (Fig. 3E, F) expression by immunocytochemistry in hPSC-CECs derived from clone 10(+ CR2) treated with doxycycline compared to the PBS control. We also observed a variable reduction in CD31 expression in the doxycycline-treated populations, with CD31 expression at similar levels to the PBS control in some colonies and significantly reduced CD31 expression in cells outside of these colonies. In addition, we observed reduced claudin-5 expression in Dox-treated cells relative to PBS control, which also correlates with lower CD31 expression (Figure S9A, B). Consistent with these tight junction protein expression patterns, treatment of PB-TRE-N1ICD hPSC-CECs with doxycycline did not increase transendothelial electrical resistance (TEER) compared to untreated controls over 10 days (Figure S10). While there was very little if any P-glycoprotein (P-gp) expression in either condition, no difference was detected between Dox and PBS (Figure S9C, D). Occludin expression was undetectable by immunocytochemistry both in the PBS control and Dox-treated cells as previously reported for hPSC-CECs at this stage of maturity [27] (Figure S9E). The protein-level changes in caveolin-1, GLUT-1, and PLVAP were consistent with gene expression changes described above (Figure S8) and those seen in the N1ICD LV modified hPSC-CECs (Fig. 1). Similar to the reduced CD31 expression that was observed with increased N1ICD LV transduction in hPSC-CECs, the heterogeneity in CD31 expression among Dox-treated PB-TRE-N1ICD hPSC-CECs suggests varying degrees of Notch signaling activation among the population of endothelial cells.

### Sequencing analysis of hPSC-derived CECs with doxycycline-inducible overexpression of *N1ICD*

**Fig. 4:**
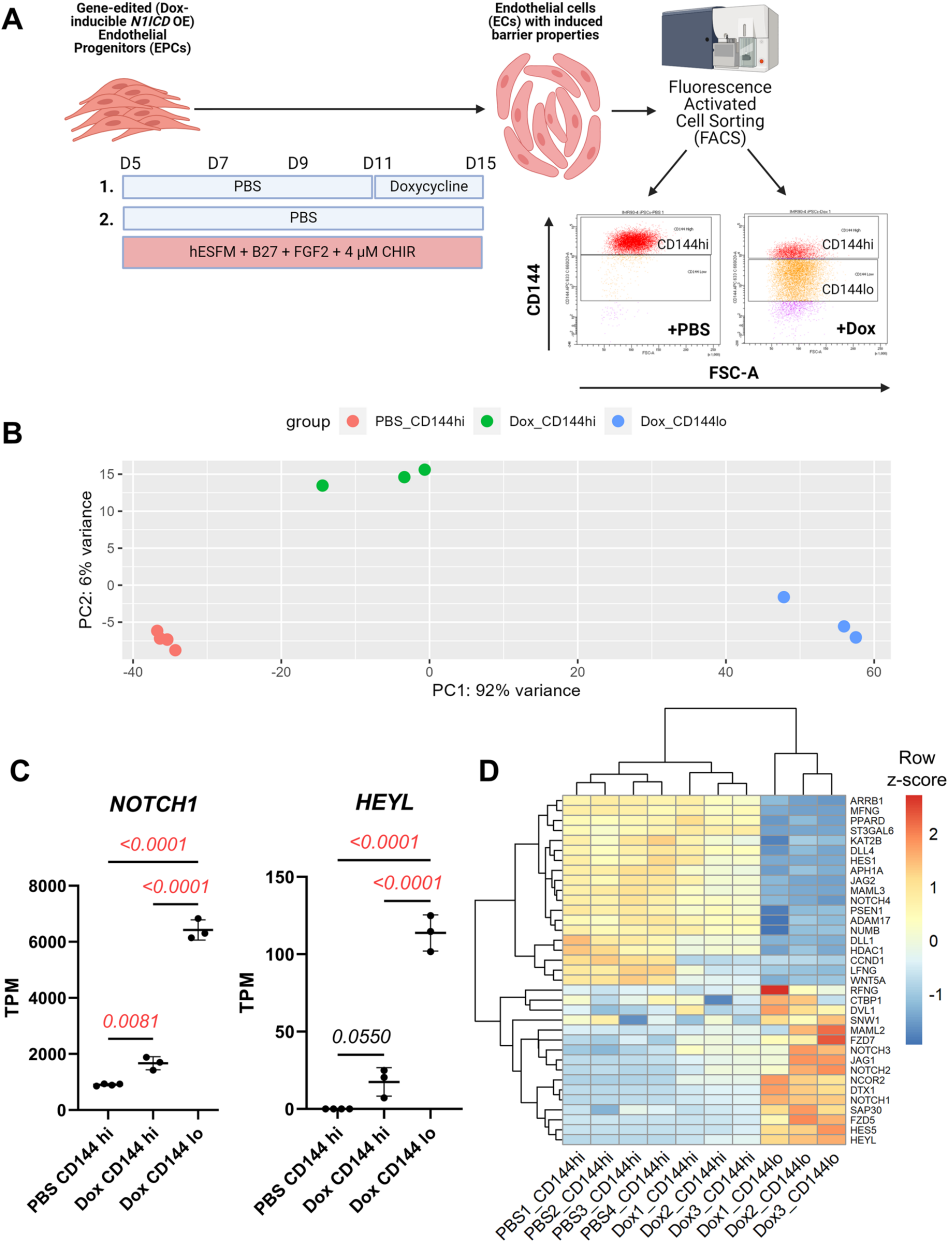
FACS sorting and bulk RNA-sequencing of hPSC-CECs with doxycycline-inducible N1ICD overexpression: **(A)** Schematic of culture timeline and FACS sorting for PB-TRE-N1ICD 10(+ CR2) hPSC-CECs treated with or without doxycycline to overexpress *N1ICD*. Cells in each PBS- (*n* = 4) and Dox-treated (*n* = 3) sample were sorted by FACS into subpopulations based on CD144 (VE-cadherin) expression. Lower boundary for CD144^high^ gate was set based on majority of PBS-treated hPSC-CECs and unedited hPSC-CECs cultured in the presence of CHIR alone. Lower boundary for CD144^low^ gate was set based on CD144 antibody-stained undifferentiated hPSCs and isotype control antibody-stained edited hPSC-CECs. Gated plots show representative distributions of PBS- and Dox-treated edited hPSC-CECs into CD144^high^ and CD144^low^ expressing subpopulations. **(B)** PCA plot showing relative differences in transcriptomic profiles of bulk RNA-sequenced subpopulations from (A), including PBS-treated CD144^high^ (PBS_CD144hi), Dox-treated CD144^high^ (Dox_CD144hi), and Dox-treated CD144^low^ (Dox_CD144lo). Raw bulk RNA-sequencing data were pre-processed using the Galaxy pipeline (usegalaxy.org) discussed in Materials & Methods and analyzed in RStudio using the DESeq2. **(C)** Normalized expression (transcripts per million, TPM) of *NOTCH1* and *HEYL* from each bulk RNA-sequenced subpopulation discussed in (**A**) and (**B**). Statistics calculated by one-way ANOVA with post-hoc Tukey’s test, significant comparisons highlighted in red. **(D)** Heatmap of row-normalized TPM expression for Notch signaling-related genes that are significantly differentially expressed (FDR < 0.05) between the 3 subpopulations. Selected genes obtained from KEGG and Hallmark Notch signaling gene sets via MSigDB (https://www.gsea-msigdb.org/gsea/msigdb). Row z-scores were calculated for each gene by subtracting mean expression across all samples from normalized expression for a specific sample and dividing the result by the standard deviation. Row z-scores range from bright red, indicating high expression, and dark blue, indicating low expression. Heatmap was produced in RStudio using the pheatmap package

Next, to understand the broader effects of *N1ICD* overexpression on BBB- and cell-type-specific gene expression, we performed RNA sequencing on the PB-TRE-N1ICD 10(+ CR2) hPSC-CECs with and without doxycycline-induced *N1ICD* overexpression. We performed fluorescence activated cell sorting (FACS) on PBS- or doxycycline-treated 10(+ CR2) edited hPSC-CECs based on surface expression of CD144 (*CDH5*, VE-cadherin) (Fig. 4A). The majority (~ 94%) of the live cells in the PBS-treated condition were CD144+. In the doxycycline-treated population, the majority (~ 90%) of the cells were CD144+ (Fig. 4A), but we observed a range of CD144 expression levels akin to what was observed with CD31 levels above (Fig. 3 and Figure S4A). Therefore, these cells were sorted as two subpopulations, one with high CD144 expression (~ 25%, similar to the vast majority of the PBS-treated cells), and one with reduced CD144 expression (~ 65%) for subsequent analyses (Fig. 4A). After FACS, RNA was isolated independently from the CD144 high and low subpopulations for the doxycycline-treated samples and the CD144 high PBS-treated sample and analyzed by paired-end read bulk RNA-sequencing.

Samples from each of the three sequenced subpopulations were analyzed using DESeq2 to identify differentially expressed genes between each of the doxycycline-treated subpopulations (Dox-treated CD144 high and CD144 low) and the PBS control (PBS-treated CD144 high). When visualized using principal component analysis (Fig. 4B), the PC1 axis, which accounts for 92% of variance between samples, is the primary axis along which samples from the different subpopulations segregated. PBS-treated CD144 high (PBS_CD144hi) was most different from Dox-treated CD144 low (Dox_CD144lo) and slightly less different from Dox-treated CD144 high (Dox_CD144hi), suggesting that the PC1 axis defines the increasing *N1ICD* expression level. Normalized *NOTCH1* expression (TPM) exhibited a ~ 2-fold increase from PBS-treated CD144 high (PBS CD144 hi) to Dox-treated CD144 high (Dox CD144 hi) and a nearly 7-fold increase from PBS CD144 hi to Dox-treated CD144 low (Dox CD144 lo) (Fig. 4C). Furthermore, we observed an increase in expression of *HEYL*, a gene downstream of Notch signaling activation, which was proportional to the increase in *NOTCH1* expression (Fig. 4C). Next, we examined a gene list derived from a combination of the Hallmark and KEGG Notch signaling pathway gene sets to see which of the Notch-related genes were significantly differentially expressed between the three sequenced subpopulations (Fig. 4D). 35 genes were differentially expressed, encompassing Notch receptors and ligands as well as Notch-signaling associated transcription factors. Around half of these genes were up- or down-regulated in response to increasing degrees of *N1ICD* overexpression. All genes encoding Notch receptors were upregulated with greater *N1ICD* overexpression dosage except for *NOTCH4*, which was downregulated in the highest *N1ICD* overexpressing subpopulation (Dox CD144 lo). Differentially expressed Notch ligands, in contrast, such as *DLL4*, *JAG2*, and *DLL1*, were primary downregulated with increasing *N1ICD* overexpression. Only *JAG1* was upregulated in Dox CD144 lo. HEY/HES family transcription factors were variably differentially expressed; *HEYL* and *HES5* were significantly upregulated and *HES1* significantly downregulated in the subpopulation with the greatest degree of *N1ICD* overexpression. The differential regulation of Notch target genes is expected as *N1ICD* overexpression can lead to both upregulation and feedback inhibition of downstream genes [40].

**Fig. 5:**
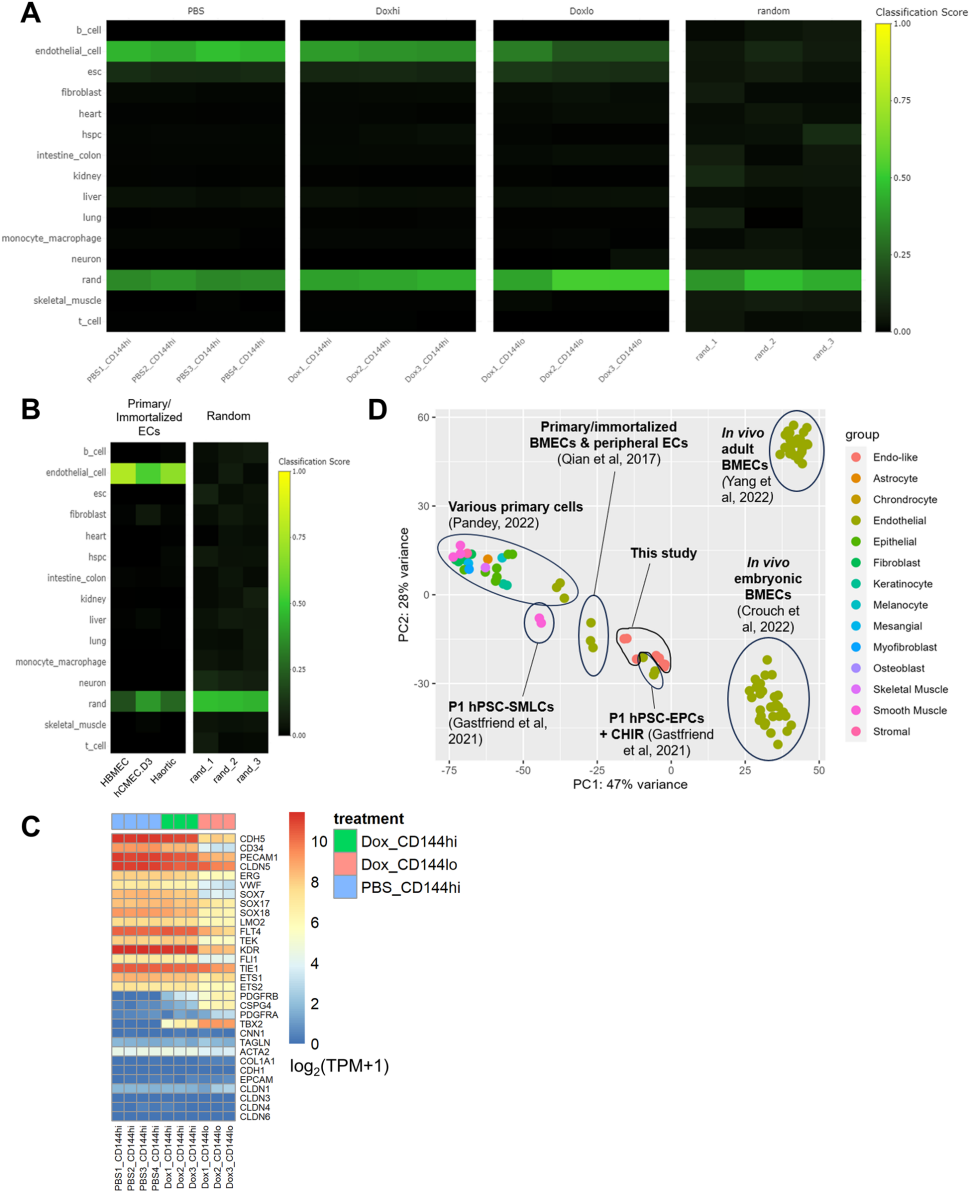
Transcriptomic comparison of PB-TRE-N1ICD hPSC-CECs ± Dox to hPSC-derived, primary or immortalized endothelial and non-endothelial cell types and to in vivo BMECs: **(A)** Analysis of bulk transcriptomic data of subpopulations of PB-TRE-N1ICD 10(+ CR2) hPSC-CEC subpopulations using PACNet [31] classification scores to identify similarity to generic cell types in a training data set. Sample replicates from left to right: PBS-treated, CD144-high (PBS_CD144hi); doxycycline-treated, CD144-high (Dox_CD144hi); doxycycline-treated, CD144-low (Dox_CD144lo); random cell type (rand) auto-generated by PACNet software. Classification score colors ranging from black to yellow indicate no transcriptomic resemblance to 100% transcriptomic match, respectively, to training data cell types indicated on y-axis. **(B)** PACNet analysis of primary human brain microvascular endothelial cells (HBMEC), immortalized hCMEC.D3 brain endothelial cells (hCMEC_D3), and primary human aortic endothelial cells (Haortic) relative to training set cell types. All analyses in (A) and (B) were performed using the PACNet webtool (https://cahanlab.org/resources/agnosticCellNet_web/). **(C)** Heatmap of transcript abundance (log_2_[TPM + 1]) of indicated endothelial, mesenchymal, fibroblast, and epithelial genes across samples with different degrees of *N1ICD* overexpression. Heatmap columns indicate sample/replicate and rows indicate genes. Cell color indicates transcript abundance, ranging from blue (lowest expression) to red (highest). Heatmaps were generated using the pheatmap package in R. **(D)** PCA plot combining several in vivo and in vitro transcriptomic datasets, including: pseudo-bulked in vivo adult brain capillary endothelial cell scRNA-seq data (Yang et al., 2022) [33]; pseudo-bulked in vivo embryonic brain capillary endothelial cells scRNA-seq data (Crouch et al., 2022) [32]; in vitro primary/immortalized brain endothelial and primary peripheral endothelial cells (similar to those presented in [B]) bulk RNA-seq data (Qian et al., 2017) [41]; in vitro Passage 1 hPSC-derived endothelial progenitor cells (EPCs) treated with CHIR and hPSC-derived smooth muscle-like cells (SMLCs) bulk RNA-seq data (Gastfriend et al., 2021) [25]; in vitro primary cells representing different developmental lineages bulk RNA-seq data (Pandey, 2022, GSE190615); in vitro PB-TRE-N1ICD hPSC-CECs with or without doxycycline bulk RNA-seq data (this study). PCA plot was generated using the DESeq2 package in R

We next asked if the cells with reduced CD144 expression maintained an endothelial identity, given the reduction in CD144 expression that was observed in a significant proportion of the doxycycline-treated 10(+ CR2) PB-TRE-N1ICD hPSC-CECs. We used Platform-Agnostic CellNet (PACNet) [31] to compare the transcriptomes of each of the sequenced PBS- and doxycycline-treated subpopulations to transcriptomes of several standardized cell and tissue types used by the PACNet algorithm (Fig. 5A). For each of the subpopulations, the classification score was highest for endothelial cells, and nearly zero for all other cell types, including ESCs or hPSCs, fibroblasts, and skeletal muscle. However, there was a nonzero classification score for a randomly generated cell type “rand” derived from the combination of multiple transcriptomes of the training cell types, a feature that was shared with the endothelial classification scores of primary or immortalized human brain endothelial cells (HBMEC and hCMEC.D3, respectively), or primary human aortic endothelial cells (Haortic) (Fig. 5B). Taken together, these data suggest that while the transcriptomes of the edited endothelial cells with or without *N1ICD* overexpression were not identical to the generic primary human endothelial cells used to train the PACNet algorithm, they maintained their endothelial cell identity and did not transform into another cell type classified by this algorithm. Furthermore, we examined transcript abundance for a list of endothelial, mesenchymal, fibroblast, and epithelial genes across all samples [25] (Fig. 5C). While slight decreases in endothelial transcript abundance were observed, these reductions were mainly observed in the subpopulation of Dox-treated cells with the greatest degree of *N1ICD* overexpression (Dox CD144 lo). Endothelial transcript abundance in the Dox CD144 hi subpopulation was relatively similar to that of the PBS control (PBS CD144 hi). Although a few mesenchymal genes such as *PDGFRB*,* CSPG4*, and *TBX2* were increased by *N1ICD* overexpression, most of the remaining non-endothelial genes in our list were unchanged, including fibroblast marker *COL1A1* and epithelial marker *EPCAM*. Notably, the transcript abundances for most of these genes (including *PDGFRA*, *CNN1*, *TAGLN*, *ACTA2*, *COL1A1*, *CDH1*, *CLDN1*, *CLDN3*, *CLDN4*, and *CLDN6*) were extremely low relative to endothelial transcripts, even in cells with the highest degree of *N1ICD* overexpression. As an additional set of comparisons, we used principal component analysis to compare the global transcriptomes of the subpopulations that were sequenced as part of this study to the transcriptomes of a host of other endothelial and non-endothelial cell types (Fig. 5D). The comparison bulk RNA sequencing datasets included Passage 1 hPSC-derived EPCs treated with CHIR (hPSC-CECs from [25]), Passage 1 hPSC-derived smooth muscle-like cells that are a byproduct of the hPSC-EPC differentiation [25], primary human brain microvascular endothelial cells, hCMEC/D3s (immortalized human brain microvascular endothelial cells), primary human aortic endothelial cells [41], and a variety of primary cells including endothelial, epithelial, smooth muscle, skeletal muscle, and fibroblast cells (Pandey et al., GEO accession: GSE190615). Furthermore, we generated pseudo-bulk transcriptomes of human brain capillary, arterial, and venous endothelial cells from adult [33] and embryonic [32] single cell RNA-sequencing datasets. The different cell types largely segregated along PC1 (47% of variance) which ranged from in vitro cells with the least endothelial character on the left to in vitro ECs including those from this study to in vivo endothelial cells with BBB properties on the right. The transcriptomes of the cells sequenced as part of this study were most similar to the Passage 1 hPSC-EPCs + CHIR (hPSC-CECs); this is unsurprising because the same differentiation protocol was used to derive the hPSC-CECs from the PB-TRE-N1ICD 10(+ CR2) hPSC line. These data indicate that the PB-TRE-N1ICD hPSC-CECs with or without doxycycline retained endothelial character but were more similar to in vitro cultured brain endothelial cells than to in vivo embryonic or adult human brain capillary endothelial cells.

### Evaluating the impact of *N1ICD* overexpression on the BBB transcriptome

**Fig. 6:**
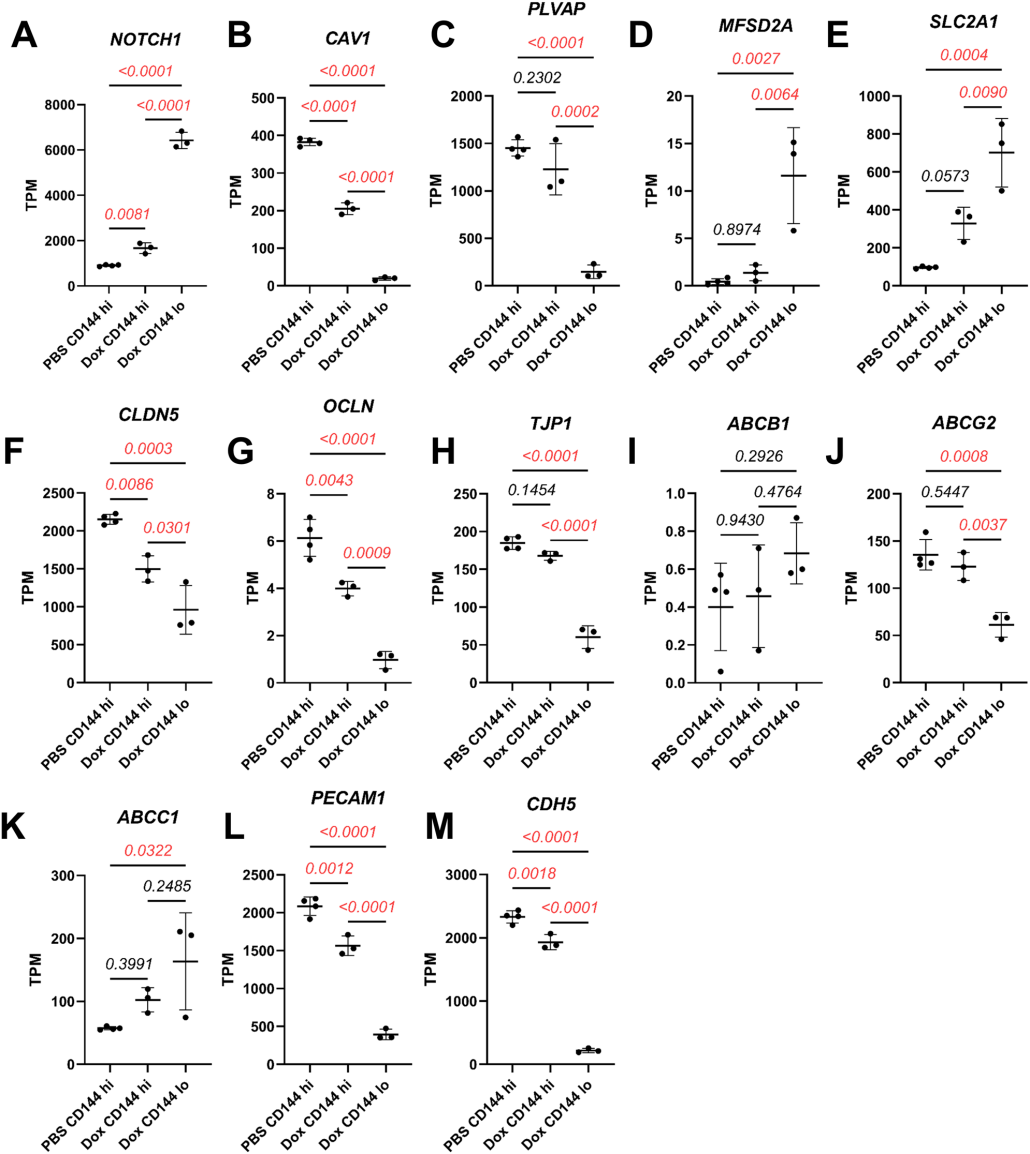
Normalized expression of selected BBB and cell identity-related genes from bulk RNA-seq of edited PB-TRE-N1ICD 10(+ CR2) hPSC-CECs ± Dox subpopulations: **(A-M)** Comparison of normalized gene expression (TPM) between bulk RNA-sequenced PBS- and Dox-treated subpopulations for indicated genes. In all analyses, points represent *n* = 3 or *n* = 4 biological replicates depending on treatment condition from one differentiation of IMR90-4 PB-TRE-N1ICD 10(+ CR2) hPSC-CECs. Bars indicate mean ± SD. Relative gene expression values are shown in TPM, and statistical analyses were performed on TPM data; P-values < 0.05 by one-way ANOVA with post-hoc Tukey’s test are highlighted in red

Next, we examined our sequencing data to determine the global effects of *N1ICD* overexpression on genes regulating key BBB properties. With respect to endocytosis and transcytosis-related genes, increasing levels of *N1ICD* overexpression (Fig. 6A) correlated with decreased *CAV1* and *PLVAP* and increased *MFSD2A* expression (Fig. 6B-D). *SLC2A1* expression was also significantly increased upon *N1ICD* upregulation (Fig. 6E). However, we observed downregulation of tight junction genes *CLDN5*,* OCLN*, and *TJP1* (Fig. 6F-H) as a function of *N1ICD* overexpression. Changes in efflux pump genes were varied; *ABCB1* was unchanged, *ABCG2* decreased, and *ABBC1* increased in response to *N1ICD* overexpression (Fig. 6I-K). These data confirmed a gene-dosage dependent role of Notch signaling activation in differential regulation of several BBB-related genes.

We next evaluated the effects of Notch signaling activation on a broader set of 310 genes which are up- or downregulated in vivo in brain endothelial cells relative to peripheral organ endothelial cells based on an integrated single-cell RNA-sequencing analysis of multiple publicly available datasets from several organs, including the brain (from reference [22]). Of the 173 genes upregulated in brain vs. peripheral endothelial cells (Figure S11A), 46 exhibited increased expression in response to Notch signaling activation in our PB-TRE-N1ICD hPSC-CECs. These include *SLC2A1* and *MFSD2A*, as previously shown, but also a host of other BBB-enriched genes, including many SLC nutrient transport genes such as *SLC7A6*,* SLC7A5*,* SLC7A2*,* SLC7A1*,* SLC5A6*,* SLC2A3*,* SLC1A5*, and *SLC1A4* (Figures S11A and S12F). Also, 56 of the 173 genes upregulated at the BBB in vivo were downregulated after *N1ICD* overexpression, including tight junction genes mentioned in Fig. 6, as well as *LSR*, which encodes the lipolysis-stimulated lipoprotein receptor, a component of tri-partite paracellular junctions in CNS endothelium [6] (Figure S12B). The remaining 61 of 173 genes upregulated in brain endothelial cells in vivo were not detected or not differentially expressed due to *N1ICD* overexpression. Of the 137 genes downregulated in brain vs. peripheral endothelial cells (Figure S11B), 50 were downregulated upon *N1ICD* overexpression including several related to transcytosis, as mentioned in Fig. 6. When we examined transcytosis-related genes more closely, caveolae-mediated transcytosis genes were primarily downregulated, whereas genes related to clathrin-mediated endocytosis and macropinocytosis (e.g. *CLTA*,* CLTB*,* CLTC*, and *APPL1*, respectively) were differentially expressed depending on the degree of *N1ICD* overexpression in the Dox-treated subpopulation (Figure S12A). Generally, clathrin-related genes were more likely to be upregulated in response to increased Notch signaling. Of the 137 genes downregulated in brain vs. peripheral endothelial cells, 27 were upregulated and 59 were not detected or not differentially expressed due to *N1ICD* activation, although many of the genes in these two categories are not obviously associated with classical BBB phenotypes. Several adherens junction genes besides *CDH5* and *PECAM1* were also downregulated in response to *N1ICD* overexpression, including *CD44* and *MCAM* (Figure S12D); however, the expression of *CDH5*, *PECAM1*, and *MCAM* remained in the range of 200–500 TPM. We also noted decreased expression of several endothelial-associated transcription factors after *N1ICD* overexpression (Figure S12E), although the normalized expression levels remained quite high. Overall, comprehensive examination of gene expression in hPSC-CECs following Notch signaling activation revealed changes in transcytosis pathway-related transcripts as well as increased expression of numerous BBB-specific nutrient transporters.

### Functional characterization of vesicles and endocytic trafficking

**Fig. 7:**
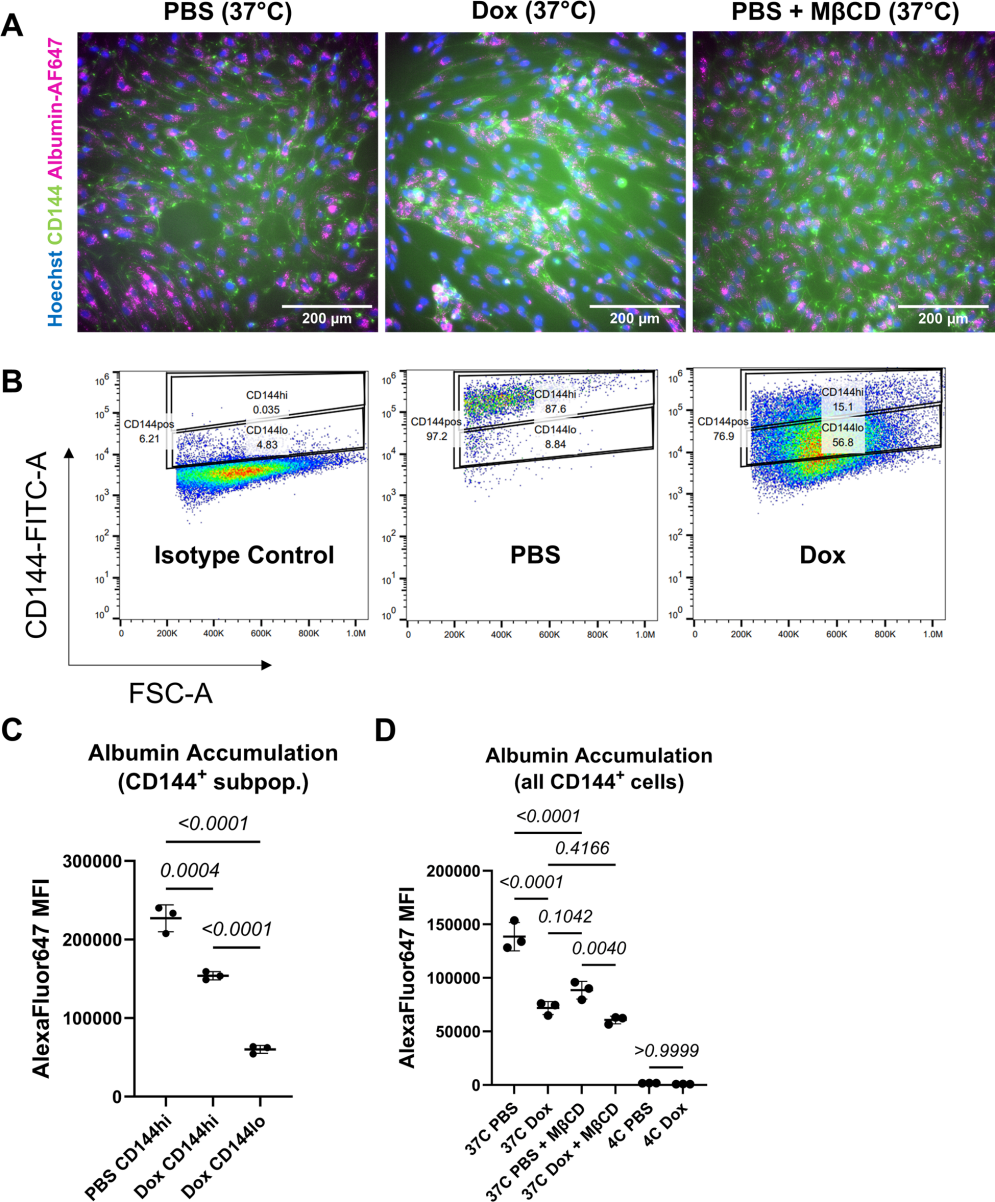
*N1ICD* overexpression reduces fluorescent albumin accumulation by inhibiting caveolae-mediated endocytosis: **(A)** Representative live cell epifluorescence microscopy images of 10(+ CR2) PB-TRE-N1ICD hPSC-CECs cultured in hECSR + CHIR, with PBS or Dox, and PBS-cultured cells with methyl-β-cyclodextrin (MβCD, inhibitor of caveolae-mediated endocytosis) pretreatment that were incubated at 37 °C with bovine serum albumin, or BSA, conjugated to AlexaFluor 647 (albumin-AF647). CD144 (VE-cadherin) and AlexaFluor 647 are shown in the 488 and 647 nm channels, respectively. Hoechst nuclear counterstain is overlaid in all images. Scale bar: 200 μm. **(B)** Representative flow cytometry plots for isotype control, PBS- and Dox-treated cells from the fluorescent albumin accumulation assay showing gating of the CD144^high^ (CD144hi) and CD144^low^ (CD144lo) subpopulations. **(C)** Quantification of albumin-AF647 accumulation in gated subpopulations incubated at 37 °C. Statistical comparisons shown between accumulation for each individual subpopulation. **(D)** Quantification of albumin-AF647 accumulation in all CD144-positive cells from each condition, with or without MβCD pretreatment. Conditions incubated at 37 °C as well as 4 °C control were included. Selected statistical comparisons are shown among PBS- or Dox-treated conditions in the presence and absence of MβCD-pretreatment. Statistical analyses for (**C**) and (**D**) were performed on raw MFI data; P-values < 0.05 by one-way ANOVA with post-hoc Tukey’s test

We next asked whether the reduced caveolin-1 and PLVAP expression in hPSC-CECs with *N1ICD* overexpression correlated with decreased albumin trafficking. To this end, we performed fluorescent substrate accumulation assays with albumin (~ 66 kDa) conjugated to Alexa Fluor 647 in PB-TRE-N1ICD hPSC-CECs treated with or without doxycycline. Albumin is a serum protein that is trafficked across healthy CNS endothelium at much lower levels than peripheral endothelium [42]. Furthermore, while caveolin-mediated endocytosis is a major pathway of albumin uptake by endothelial cells, caveolin-independent pathways including clathrin-mediated endocytosis and macropinocytosis can also participate in albumin trafficking [43, 44]. We imaged accumulation of fluorescent albumin by epifluorescence microscopy (Fig. 7A) and also performed flow cytometry to quantify albumin accumulation (Fig. 7B) in the PBS CD144 hi, Dox CD144 hi, and Dox CD144 lo subpopulations, and found that albumin accumulation inversely correlated with levels of *N1ICD* overexpression (Fig. 7C).

We next explored if Notch signaling activation caused the reduction in albumin accumulation via a specific reduction in caveolae-mediated endocytosis. PBS- and Dox-treated hPSC-CECs were treated with methyl-β-cyclodextrin (MβCD), an inhibitor of caveolae-mediated endocytosis [45]. We found that the reduction in albumin accumulation achieved by blocking caveolae-mediated endocytosis alone (PBS + MβCD) was similar in magnitude to the reduction achieved simply by upregulation of Notch signaling (Dox). However, when comparing PBS + MβCD and Dox + MβCD, there was still a slight but significant reduction in albumin accumulation when Notch signaling activation was combined with blockade of caveolae-mediated endocytosis (Fig. 7D). This suggests that although Notch signaling primarily resulted in reduction of caveolae mediated endocytosis, other endocytic pathways were also reduced when this pathway is activated in hPSC-CECs. Therefore, we investigated other pathways for substrate trafficking across endothelial cells, including clathrin-mediated endocytosis and macropinocytosis [13]. We inhibited clathrin-mediated endocytosis with chlorpromazine (CPZ) and macropinocytosis with rottlerin, as previously described [25]. Albumin accumulation was reduced when PBS-treated cells were incubated with CPZ, but not with rottlerin (Figure S13A), suggesting in addition to caveolae-mediated endocytosis, clathrin-mediated endocytosis is another mechanism for intracellular accumulation of albumin. Thus, Notch signaling activation via *N1ICD* overexpression reduces caveolae- and clathrin-mediated endocytosis, but not macropinocytosis, and results in reduced accumulation of albumin in the PB-TRE-N1ICD hPSC-CECs in vitro.

We confirmed the phenotypic effects of reduced caveolar-based trafficking in *N1ICD* stem cell clones 3(+ Y), 9(+ Y), and 11(+ CR2). Fluorescent albumin accumulation was decreased in the 2 clones (3[+ Y] and 11[+ CR2]) having the highest *N1ICD* upregulation, and the fold reduction in Alexa Fluor 647 signal intensity correlated with the degree of *N1ICD* upregulation in each of these clones (Figure S13B-D, Figure S8A). Finally, we investigated whether HUVECs transduced with *N1ICD* overexpressing lentivirus also demonstrated reduced albumin accumulation since they also exhibited decreased caveolin-1 expression. We confirmed reduced albumin accumulation in these primary human endothelial cells (Figure S13E) with *N1ICD* overexpression; however it was not reduced to the level afforded by the caveolae inhibitor MβCD. This is perhaps unsurprising given that the level of N1ICD protein induced by lentiviral overexpression was much lower than with Dox-inducible overexpression in our edited cell line (Figure S2E, Fig. 2D).

**Fig. 8:**
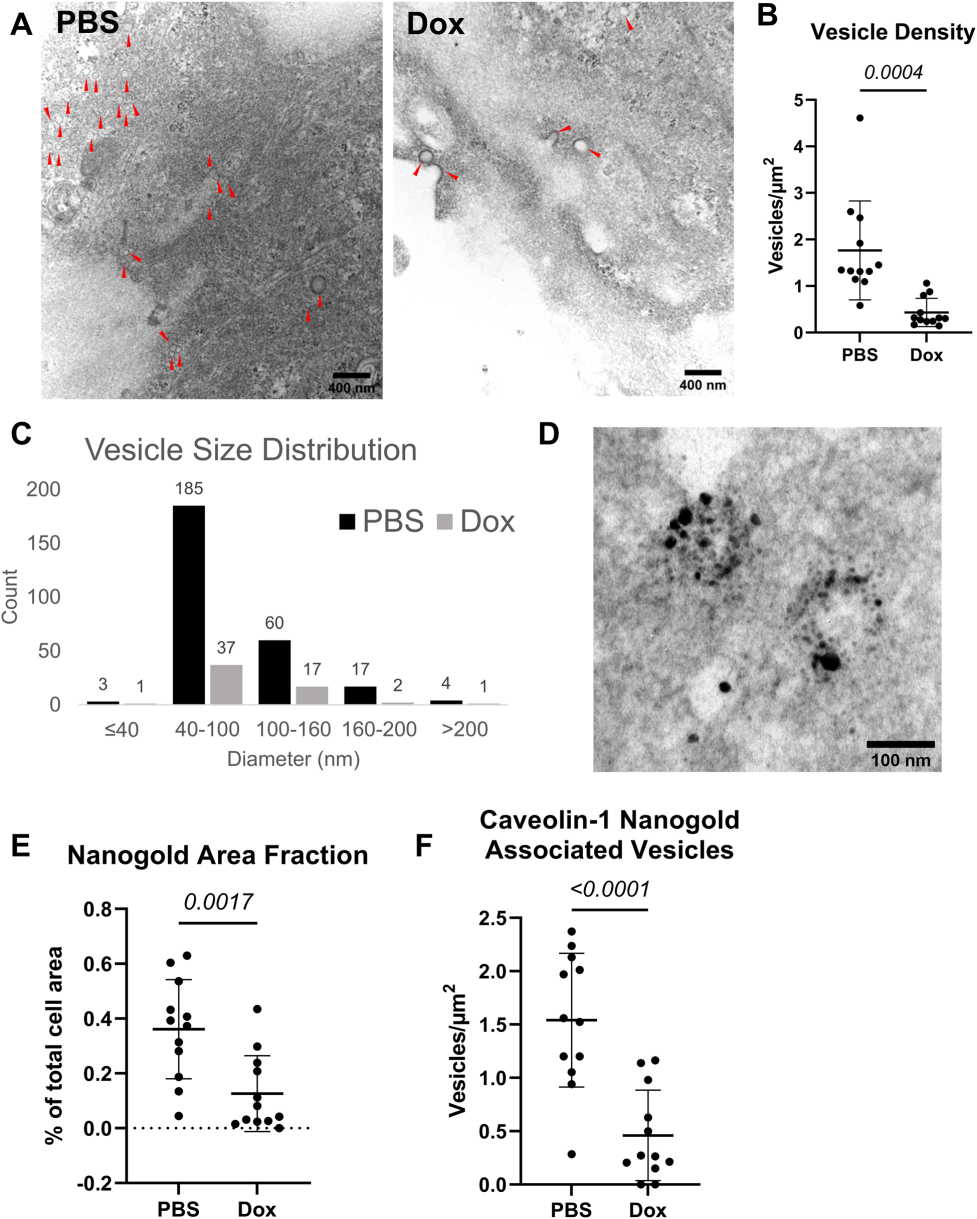
*N1ICD* overexpression reduces abundance of total and caveolae-associated vesicles: Transmission electron microscopy (TEM) analysis of PBS- or Dox-treated PB-TRE-N1ICD 10(+ CR2) hPSC-CECs. **(A)** Representative ultrastructural TEM images. Red arrowheads indicate locations of vesicles. Scale bar: 400 nm. **(B)** Quantification of vesicle density relative to total cell area for each image. **(C)** Quantification of vesicle count per diameter range across all 12 images for PBS- or Dox-treated conditions. **(D)** Representative immunolabeled TEM image of Nanogold particles bound to caveolin-1 in PBS-treated cells. Scale bar: 100 nm. **(E)** Quantification of area fraction occupied by Nanogold particles relative to total cell area. **(F)** Quantification of caveolin-1/Nanogold-associated vesicles relative to total cell area. For (**C**), (**E**), and (**F**), points represent 3 images measured per biological replicate for *n* = 4 replicates in each treatment condition. Horizontal bars indicate mean ± SD. Statistical analyses were performed on cell area-normalized counts for (**C**) and (**F**) and on area fraction for (**E**). All P-values < 0.05 by Student’s *t*-test

Finally, we visualized and quantified vesicles in doxycycline-treated 10(+ CR2) PB-TRE-N1ICD hPSC-CECs with transmission electron microscopy (TEM). We observed fewer vesicles per cell area in Dox-treated samples relative to PBS control (Fig. 8A-B). We measured the diameter for all identifiable vesicles in both conditions and found that the majority of vesicles were of the size range for caveolae (50–100 nm) and clathrin-coated vesicles (70–150 nm) [13] (Fig. 8C). Upon treatment with doxycycline, there was a decrease in the number of vesicles between 40 and 160 nm in diameter (Fig. 8C). We immunolabeled hPSC-CECs for caveolin-1 with Nanogold-conjugated antibodies. In these images, caveolae appeared as circular regions with reduced electron density (less gray) compared to the surrounding cell area, with at least one adjacent dark, electron-dense dot, representative of a Nanogold particle (Fig. 8D). In Dox-treated cells, we observed a smaller percentage of the total cell area taken up by caveolin-1 associated Nanogold particles (Fig. 8E) and fewer caveolin-1 Nanogold-associated vesicles per cell area (Fig. 8F). These data indicate that not only did upregulation of Notch signaling via *N1ICD* overexpression reduce caveolin-1 expression, but it also reduced the average number of vesicles and the number of caveolae, specifically. Combined with the albumin uptake experiments, these data suggest that the activation of Notch-1 signaling decreases caveolae-mediated endocytosis processes.

## Discussion

Notch signaling contributes to the development and maintenance of the vasculature [46]. In this study, we demonstrated that Notch signaling plays an important role in the induction of BBB properties in hPSC-derived CNS-like endothelial cells. Most notably, Notch signaling reduced expression of caveolin-1 and PLVAP, increased expression of *MFSD2A*, and reduced albumin endocytosis with fewer caveolar vesicles in both hPSC-derived CECs and primary HUVECs. Future studies should investigate whether Notch signaling can similarly induce the BBB-like reduced vesicle-based endocytosis phenotype in primary peripheral microvascular endothelial cells. Furthermore, we demonstrated that Notch signaling activation increased expression of the glucose transporter GLUT-1 and several other BBB-enriched selective nutrient transporters. We also observed that despite a reduction in expression of endothelial markers CD31 and CD144, endothelial cells subjected to Notch signaling activation via overexpression of *N1ICD* maintained their endothelial identity and did not significantly acquire characteristics of other cell types. However, Notch activation did not improve tight junction protein expression and led to little to no induction of efflux transporter P-glycoprotein or other drug efflux pump transcripts. We showed through a combination of protein-level characterization, bulk RNA-seq, and functional assays that Notch signaling activation by overexpression of *N1ICD* in hPSC-derived CECs pre-treated with Wnt signaling activation induced BBB-like properties, particularly reduced fluid-phase endocytosis, that were not previously achieved with activation of Wnt signaling alone [25], although significant transcriptional differences existed between N1ICD-expressing hPSC-derived CECs in vitro and adult BMECs in vivo*.*

The main BBB property induced by *N1ICD* overexpression was reduced vesicular endocytosis. Our findings in the hPSC-derived CEC model are consistent with prior reports of this effect in the mouse retina [23]. Not only did we see the reduction in vesicle trafficking in developing hPSC-derived CECs, we were also able to recapitulate reduced caveolin-1 and reduced vesicular endocytosis in primary endothelial cells (e.g., HUVECs) which are terminally differentiated, indicating that the Notch signaling axis can induce BBB properties at various stages of endothelial development. This finding contrasts with BBB property-inducing signals such as canonical Wnt signaling activation which demonstrated that developmental plasticity of endothelial cells is a key determinant for responsiveness to Wnt cues [25]. We also identified several other effects of Notch activation hPSC-derived CECs. As one example, we observed reduced transcript and protein-level expression of claudin-5, as well as reduced transcript level expression of occludin, albeit no occludin protein is present in hPSC-CECs at this stage. Thus, it appears that sufficiently high *N1ICD* overexpression can reduce tight junction integrity. These findings contrast to another study which demonstrated signaling through NOTCH1 in primary human BMECs is important for maintenance of claudin-5 expression [24]; potentially drawing a distinction between the role of Notch signaling in BBB property induction vs. maintenance.

Our study also demonstrated that although the endothelial identity remains strong after *N1ICD* overexpression, there is reduced expression of endothelial markers CD31 (PECAM1) and CD144 (VE-cadherin) proportional to the extent of *N1ICD* overexpression. Interestingly, comparisons of mouse single cell transcriptomes of brain capillary endothelial and peripheral organ (e.g., lung) endothelial cells suggest that BMECs have ~ 2-fold and ~ 6-fold lower expression of *Pecam1* and *Cdh5* (encoding VE-cadherin), respectively, relative to peripheral endothelial cells [47, 48]. These observations indicate that in the process of acquiring their unique phenotype, brain endothelial cells may exhibit reductions in expression of canonical endothelial markers compared to those in capillary beds of peripheral organs. Most of the effects of Notch signaling activation, even at lower gene dosage of *N1ICD* overexpression, were on transcytosis-related transcripts and nutrient (SLC) transporters. Fewer significant changes were observed in transcripts related to tight and adherens junctions, efflux transporters, and endothelial TF-related genes except for a subset of genes at higher *N1ICD* gene dosage.

A natural question that arises upon demonstration of the effects of Notch signaling in vitro is the potential origin of those signals in vivo. Some have suggested Notch signaling in brain microvascular endothelial cells in vivo may occur through juxtacrine signaling from mural cells via unknown Notch ligands [20]. In a recent scRNA-seq analysis of the human fetal brain vasculature [32], Crouch and colleagues showed that early in development (15 gestational weeks, or GWs), brain endothelial cells of various subtypes, including arterial, capillary, venous, tip and mitotic endothelial cells, primarily express *NOTCH1* and *NOTCH4* receptor genes with arterial cells having the highest expression of *NOTCH1*. In contrast, mural cells, including classical pericytes, smooth muscle cells (SMCs), and mitotic mural cells, mainly express *NOTCH3*, as previously reported [28, 49, 50]. Most interestingly, the primary cell types expressing Notch ligand genes including *DLL4*,* JAG1*, and *JAG2* during human development are endothelial cells, particularly arterial and tip cells, suggesting that much of the BBB-inductive signaling associated with the Notch pathway may be a juxtracrine process between different endothelial subtypes [32]. Later in development (23 GWs), the primary endothelial Notch receptor is *NOTCH4*, and arterial and tip endothelial cells mainly express ligand *DLL4*, whereas SMCs in the ganglionic eminence/subventricular zone express *JAG1*, which can also potentially communicate with endothelial Notch receptors [32]. Induction of BBB-related genes coincident with upregulation in Notch-related gene expression has also been observed in vascular organoids co-cultured with neural organoids [51]. The authors observed spontaneous emergence of mural cells as well as several endothelial subtypes in these assembloids mirroring those found at the brain arteriovenous tree [32, 33, 52–54], contributing to activation of Notch signaling.

Finally, we demonstrated functional reduction of vesicular endocytosis as a result of *N1ICD* expression, predominantly via the caveolae-mediated pathway, via fluorescent albumin accumulation assays and transmission electron microscopy. Notch signaling activation also induces upregulation of *MFSD2A* in hPSC-CECs. MFSD2A is a lipid flippase enriched in BMECs that is tied to reduced levels of caveolae-mediated transport at the BBB [10, 11, 38, 55], although it may not play a direct role [56–58]. Previously, only Wnt [38] and PTEN/AKT [59] signaling were reported to mediate increased MFSD2A expression at the BBB. The present study reinforces a strong association between increased Notch signaling, *MFSD2A* upregulation, caveolin-1 and PLVAP downregulation, and reduced caveolae-mediated endocytosis.

## Conclusion

In summary, our findings illuminate the role of Notch signaling in human BBB induction and thereby contribute to the understanding of the complex interplay of various cell signals that are important for induction of BBB properties and are a step towards generation of improved human-derived in vitro BBB models. Specifically, upregulation of Notch1 signaling through N1ICD expression in an hPSC-derived model of the developing BBB induced brain endothelial characteristics at the transcriptional, protein, and functional levels. Notably, N1ICD upregulated expression of the glucose transporter GLUT1 and downregulated expression of endocytosis proteins PLVAP and caveolin1. N1ICD expression in this model resulted in a reduced abundance of vesicles and reduced caveolae-mediated endocytosis.

## Supplementary Information

Below is the link to the electronic supplementary material.

Supplementary Material 1

## Data Availability

The transcriptomic datasets generated and analyzed in this study are available at ArrayExpress (https://www.ebi.ac.uk/biostudies/arrayexpress) with accession E-MTAB-15954.

## Abbreviations

ABC: ATP binding cassette
ACTA2: Actin 2
ANOVA: Analysis of variance
APC: Allophycocyanin
APPL1: Adaptor protein, phosphotyrosine interacting with PH domain and leucin zipper 1
BBB: Blood-brain barrier
BCA: Bicinchoninic acid
BMEC: Brain microvascular endothelial cell
BSA: Bovine serum albumin
CAV1: Caveolin 1
CD: Cluster of differentiation
CDH5: Cadherin 5
cDNA: Complementary DNA
CEC: CNS-like endothelial cells
ChIP: Chromatin immunoprecipitation
CLDN: Claudin
CLT: Clathrin
CNN1: Calponin 1
CNS: Central nervous system
COL1A1: Collagen type 1 alpha chain
CPZ: Chlorpromazine
CSPG4: Chondroitin sulfate proteoglycan 4
DLL: Delta-like ligand
DMEM: Dulbecco’s modified Eagle medium
DMSO: Dimethyl sulfoxide
DOX: Doxycycline
DPBS: Dulbecco’s phosphate buffered saline
EC: Endothelial cell
EDTA: Ethylenediaminetetraacetic acid
EF-1α: Elongation factor 1 alpha
EGM2: Endothelial growth medium 2
EPC: Endothelial progenitor cell
EPCAM: Epithelial cell adhesion molecule
ESC: Embryonic stem cell
ETV2: ETS variant transcription factor 2
FACS: Fluorescence activated cell sorting
FBS: Fetal bovine serum
FGF2: Fibroblast growth factor 2
FITC: Fluorescein isothiocyanate
GA: Glutaraldehyde
GFP: Green fluorescent protein
GLUT1: Glucose transporter 1
GW: Gestational week
HBMEC: Human brain microvascular endothelial cell
hECSFM: Human endothelial serum-free medium
HES: Hairy and enhancer of split
HEYL: Hairy/enhancer of split related with YRPW-motif like
hPSC: Human pluripotent stem cell
HSD: Honestly significant difference
HUVEC: Human umbilical vein endothelial cell
HygR: Hygromycin resistance
ICC: Immunocytochemistry
iPSC: Induced pluripotent stem cell
IRES: Internal ribosome entry site
JAG: Jagged
JNK: Jun N-terminal kinase
KEGG: Kyoto encyclopedia of genes and genomes
LSR: Lipolysis-stimulated lipoprotein receptor
LV: Lentivirus
MACS: Magnetic activated cell sorting
MβCD: methyl-beta-cyclodextrin
MCAM: Melanoma cell adhesion molecule
MFI: Mean fluorescence intensity
MFSD2A: Major facilitator superfamily domain containing 2a
MGV: Mean gray value
N1ICD: Notch1 receptor intracellular domain
N1 FL: Notch 1 full length
OCLN: Occludin
PB: Phosphate buffer
PBS: Phosphate buffered saline
PCR: Polymerase chain reaction
PDGFR: Platelet-derived growth factor receptor
PECAM-1: Platelet endothelial cell adhesion molecule 1
PFA: Paraformaldehyde
P-gp: P-glycoprotein
PLVAP: Plasmalemma vesicle-associated protein
PTEN: Phosphatase and tensin homolog
qPCR: Quantitative polymerase chain reaction
RBPJ: Recombination signal binding protein for immunoglobulin kappa J region
RIPA: Radioimmunoprecipitation assay
ROCK: Rho kinase
RT: Reverse transcription
scRNAseq: Single cell RNA sequencing
SD: Standard deviation
SLC: Solute carrier
SMAD4: Mothers against decapentaplegic homolog 4
SMC: Smooth muscle cell
TAGLN: Transgelin
TBST: Tris-buffered saline with Tween 20
TBX2: T-box transcription factor 2
TEER: Transendothelial electrical resistance
TEM: Transmission electron microscopy
TF: Transcription factor
TGF-β: Transforming growth factor beta
TJP1: Tight junction protein 1
TPM: Transcripts per million
VEGF: Vascular endothelial growth factor
