# Supplementary material for: Notch signaling activation reduces vesicular endocytosis in human pluripotent stem cell-derived CNS-like endothelial cells
Source: Fluids Barriers CNS. 2026 Jan 16;23:22. doi: 10.1186/s12987-025-00754-6 (PMC12874909; doi:10.1186/s12987-025-00754-6)
Supplement: Supplementary file 1 — Supplementary Material 1 [file 12987_2025_754_MOESM1_ESM.docx]

**Supplementary Figures & Tables**


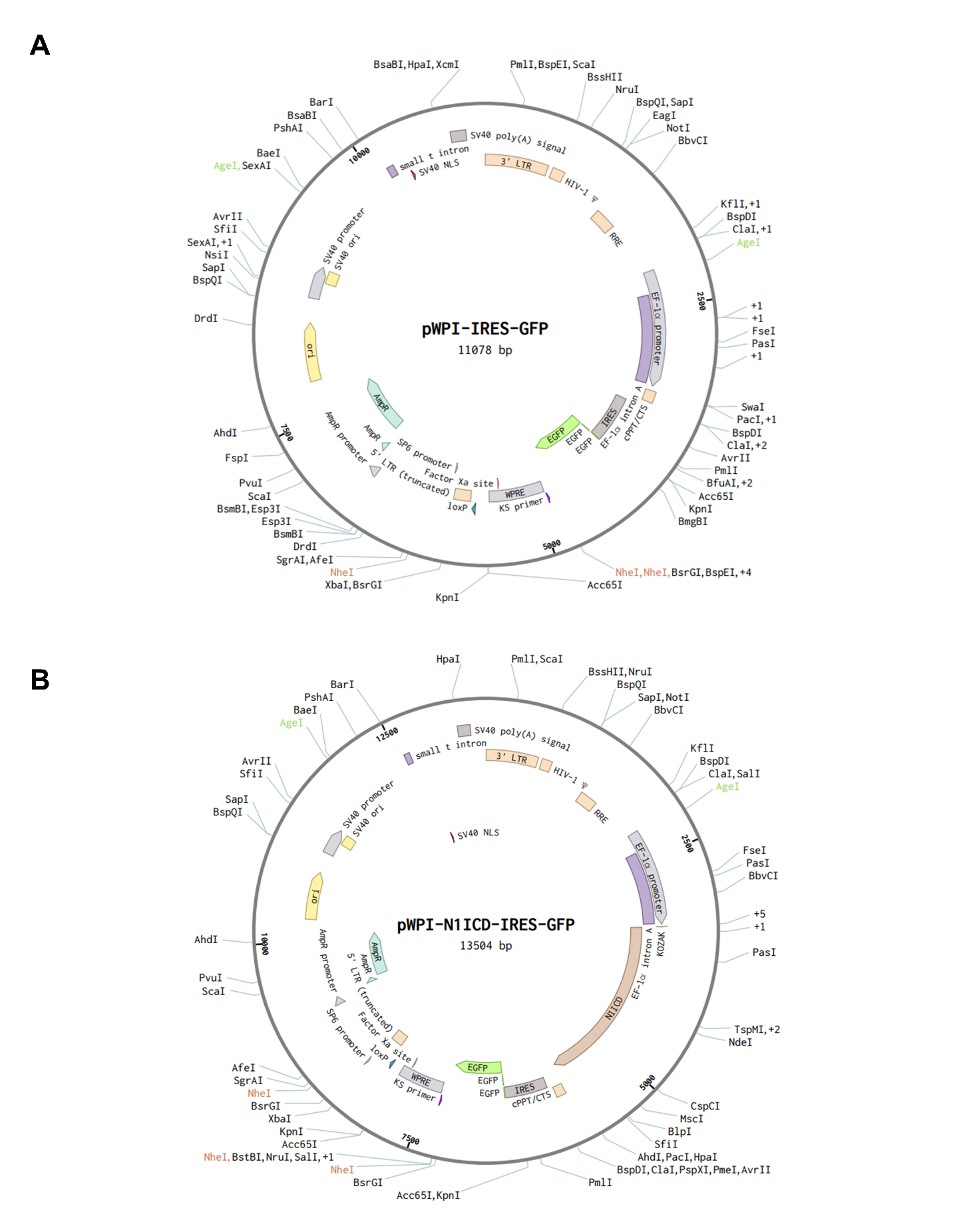


**Figure S1. Transfer plasmids for GFP and N1ICD lentiviruses: (A)** Plasmid map for pWPI (Addgene #12254). EF-1α core promoter is followed by internal ribosome entry site (IRES) and *GFP* gene. **(B)** Plasmid map for pWPI-N1ICD (Addgene #185525). Identical to pWPI with the difference that EF-1α promoter is followed by gene encoding human native Notch1 intracellular domain (*N1ICD*), which is then followed by an IRES and *GFP*. Both plasmids also contain an ampicillin resistance gene (AmpR) downstream or the aforementioned cassettes for plasmid cloning and colony selection in *E. coli*.


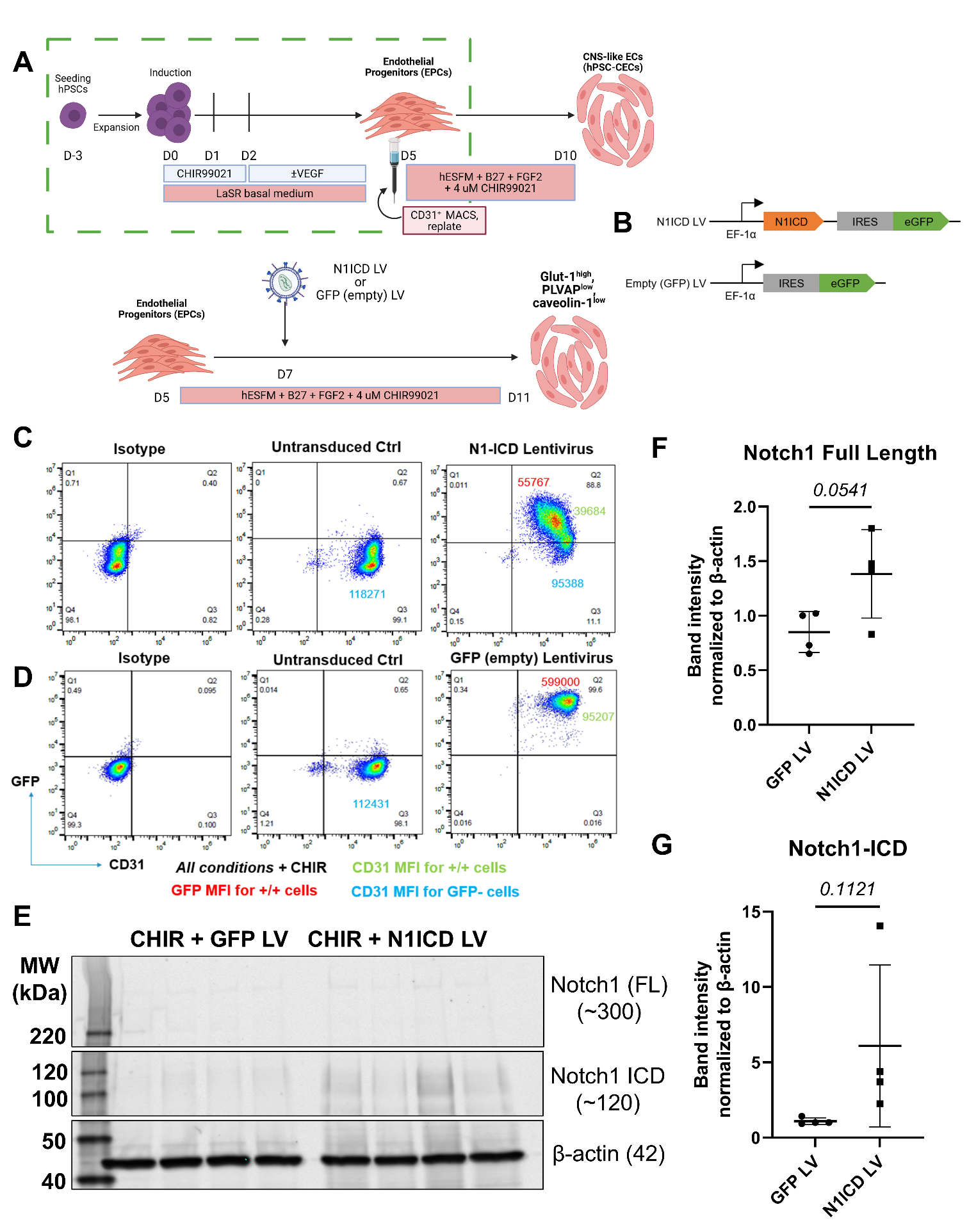


**Figure S2. Protocol for hPSC-CEC differentiation and validation of lentiviral transduction**: **(A)** Protocol for differentiation of unedited hPSCs to EPCs [26]. After hPSC-EPCs were differentiated and sorted, they were cultured in hECSR medium (hESFM + B27 + FGF2), optionally supplemented with small molecules, recombinant proteins, or genetic overexpression vehicles like lentivirus. In the case of our initial experiments, unedited hPSC-EPCs that had been cryopreserved after sorting were thawed and plated on D5, cultured in hECSR supplemented with CHIR for conversion to hPSC-CECs and on D7 they were transduced with N1ICD or GFP LV, and subsequently cultured for an additional 4 days. On D11, cells with high GLUT-1 expression and low expression of caveolin-1 and PLVAP were characterized in downstream assays. **(B)** Schematic of the lentiviral transfer plasmid constructs for GFP and N1ICD LVs. EF-1α core promoter is followed by an IRES and *GFP* for both transfer plasmids. In N1ICD LV, IRES and *GFP* are preceded by a human native *N1ICD* cassette. Representative flow cytometry plots showing GFP and CD31 fluorescence in unedited hPSC-CECs transduced with **(C)** GFP or **(D)** N1ICD LV. Each analysis used an isotype control and un-transduced control to establish gates for both markers. CD31 mean fluorescence intensity (MFI) is shown in blue for GFP negative cells. CD31 MFI and GFP MFI for GFP positive cells are shown in green and red, respectively. **(E-G)** Western blotting analysis of D11 hPSC-CECs treated with CHIR and GFP LV or CHIR and N1ICD LV. **(E)** Membrane blotted for Notch1 full length (N1 FL) protein and intracellular domain (N1ICD), followed by re-probing for β-actin. Predicted approximate molecular weights of each detected protein are indicated on the right-hand side. Quantification of Western blots band intensities for **(F)** N1 FL and **(G)** N1ICD, normalized to respective input control (β-actin) band intensities. In all Western blot analyses, points represent n = 4 biological replicates from one differentiation of IMR90-4 iPSC-derived CECs. Bars indicate mean ± SD. β-actin-normalized band intensities were further normalized within each analysis such that the mean of the CHIR + GFP LV condition was equal to 1. Statistical analyses were performed on β-actin-normalized data; P-values: Student’s *t*-test.


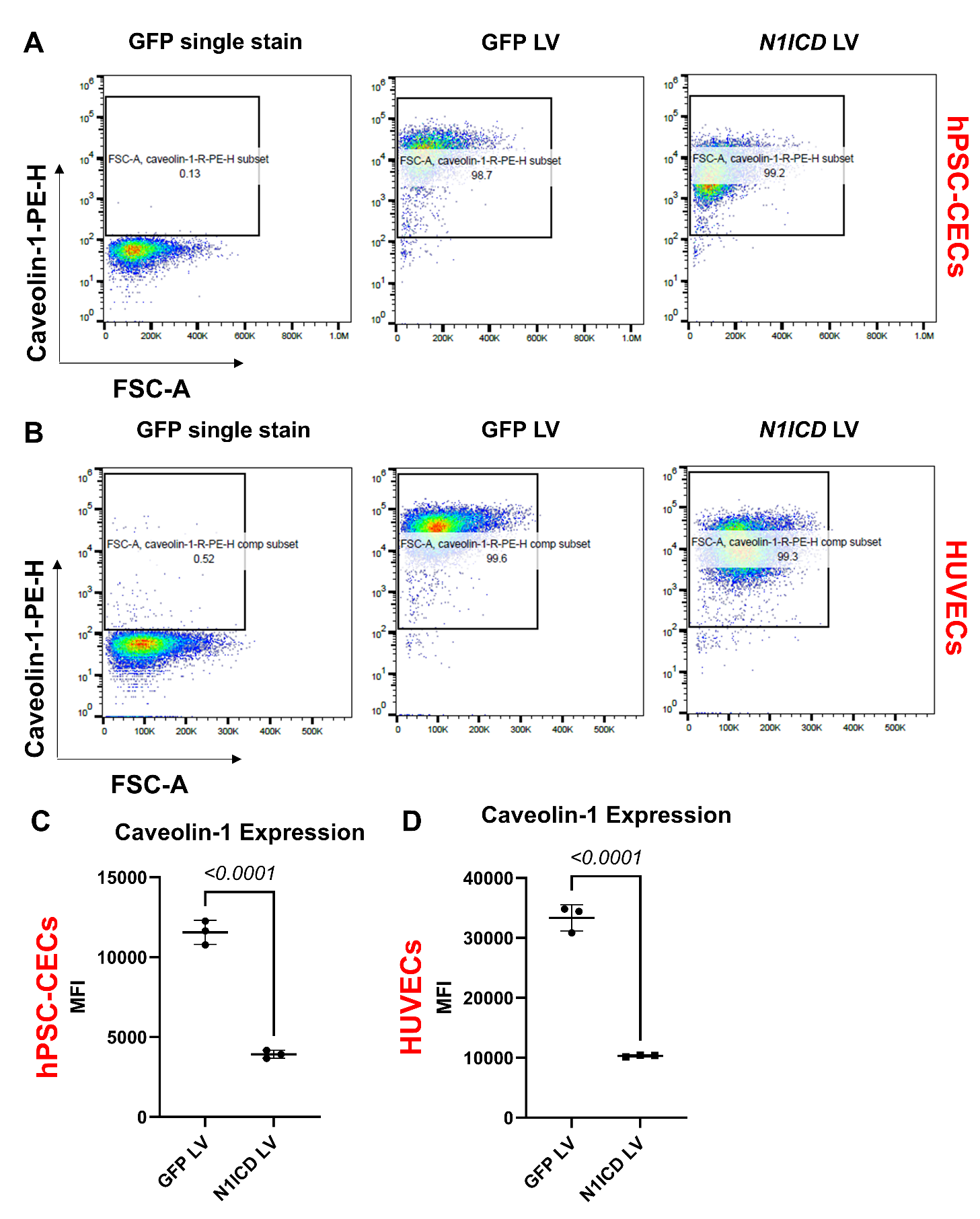


**Figure S3. Caveolin-1 flow cytometry in transduced unedited hPSC-CECs and HUVECs**: **(A)** Representative flow cytometry plots for hPSC-CECs cultured for 6 days (D5-D11) in hECSR supplemented with CHIR and transduced on D7 with GFP LV or N1ICD LV. Samples also included a GFP single stain control (transduced with GFP LV, not stained with caveolin-1-PE). **(B)** Representative flow cytometry plots for HUVECs transduced with GFP LV or N1ICD LV and cultured for 6 days in EGM-2 medium. Samples included similar controls described in (A). **(C)** Quantification of caveolin-1 MFI in hPSC-CECs treated with CHIR and GFP LV vs. CHIR and N1ICD LV. Points represent *n* = 3 biological replicates from one differentiation of IMR90-4 iPSC-derived CECs. Bars indicate mean ± SD. **(D)** Quantification of caveolin-1 MFI in HUVECs transduced with GFP LV vs. N1ICD LV. Points represent *n* = 3 biological replicates. Bars indicate mean ± SD. Statistical analyses were performed on un-normalized data; P-values: Student’s *t*-test.


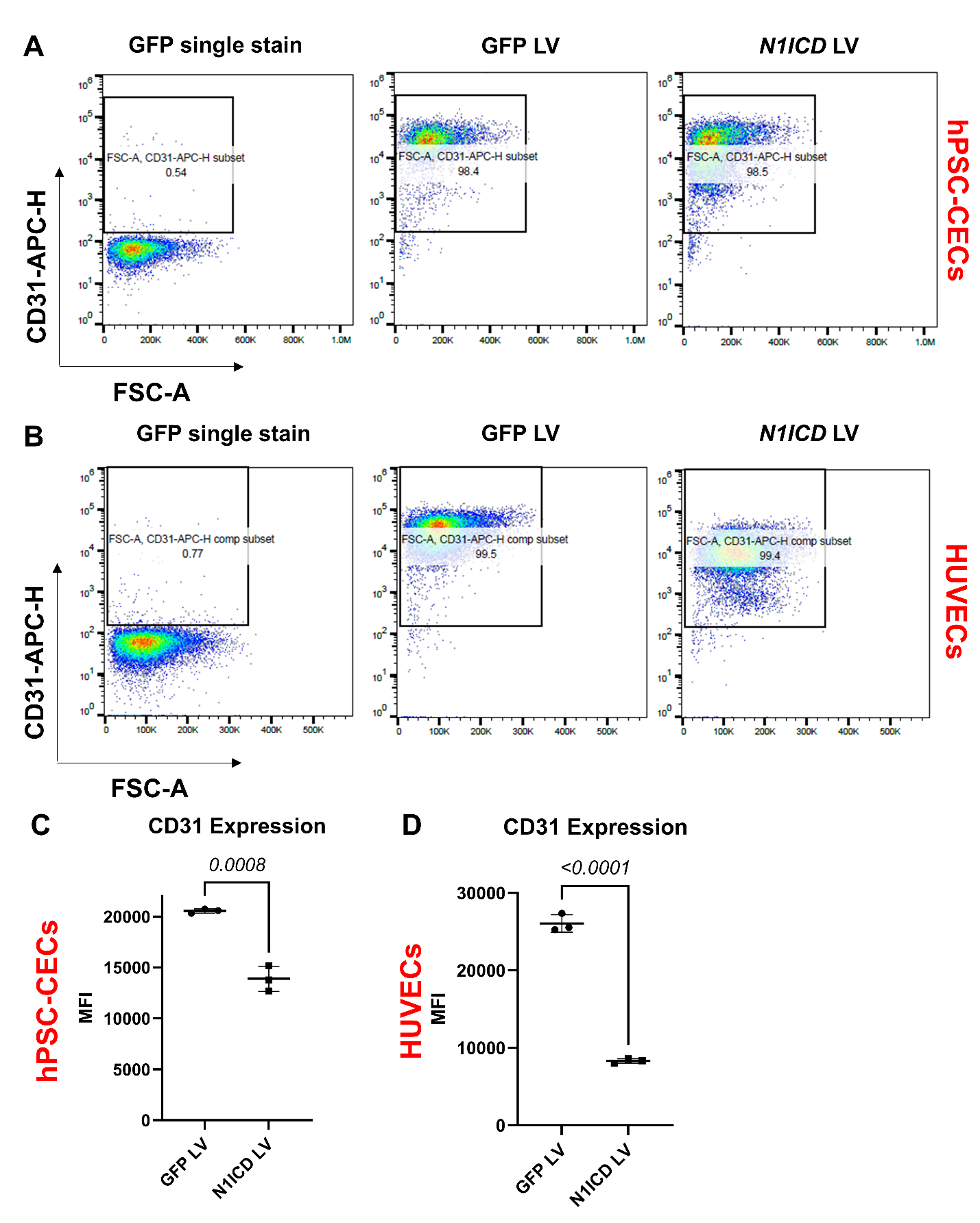


**Figure S4. CD31 flow cytometry in transduced hPSC-CECs and HUVECs**: **(A)** Representative flow cytometry plots for hPSC-CECs cultured for 6 days (D5-D11) in hECSR supplemented with CHIR and transduced on D7 with GFP LV or N1ICD LV. Samples included a GFP single stain control (transduced with GFP LV, not stained with CD31-APC) and CD31 single stain control (un-transduced, stained with CD31-APC; data not shown). **(B)** Representative flow cytometry plots for HUVECs transduced with GFP LV or N1ICD LV and cultured for 6 days in EGM-2 medium. Samples included similar controls described in (A). **(C)** Quantification of CD31 MFI in hPSC-CECs treated with CHIR and GFP LV vs. CHIR and N1ICD LV. Points represent *n* = 3 biological replicates from one differentiation of IMR90-4 iPSC-derived CECs. Bars indicate mean ± SD. **(D)** Quantification of CD31 MFI in HUVECs transduced with GFP LV vs. N1ICD LV. Points represent *n* = 3 biological replicates. Bars indicate mean ± SD. Statistical analyses were performed on un-normalized data; P-values: Student’s *t*-test.


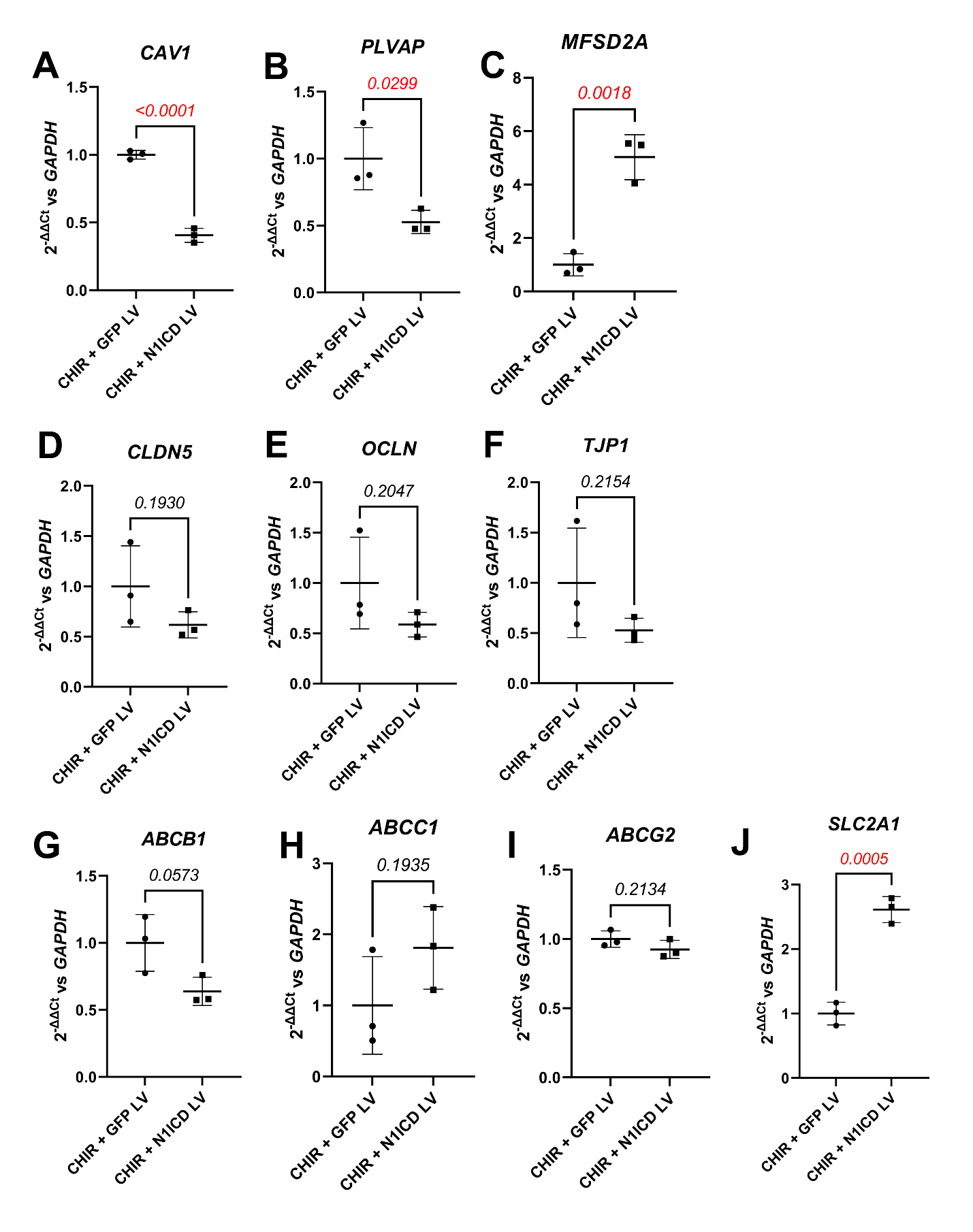


**Figure S5. Analysis of transcriptional changes in blood-brain barrier-related markers in hPSC-CECs with constitutive N1ICD overexpression:** RT-qPCR analysis of indicated BBB markers in D11 hPSC-CECs with induced barrier properties, treated with CHIR and GFP LV or CHIR and N1ICD LV. **(A-J)** Relative expression (2^-ΔΔCt^ normalized to GAPDH) of vesicular transcytosis-related genes CAV1, PLVAP, MFSD2A, tight junction-related genes CLDN5, OCLN, and TJP1, efflux transport-related genes ABCB1, ABCC1, and ABCG2, and GLUT-1 glucose transporter encoding gene SLC2A1. In all analyses, points represent n = 3 biological replicates from one differentiation of IMR90-4 iPSC-derived CECs. Bars indicate mean ± SD. Relative gene expression values normalized to GAPDH were further normalized within each analysis such that the mean of the CHIR + GFP LV condition was equal to 1. Statistical analyses were performed on normalized data; P-values < 0.05 by Student’s t-test are highlighted in red.


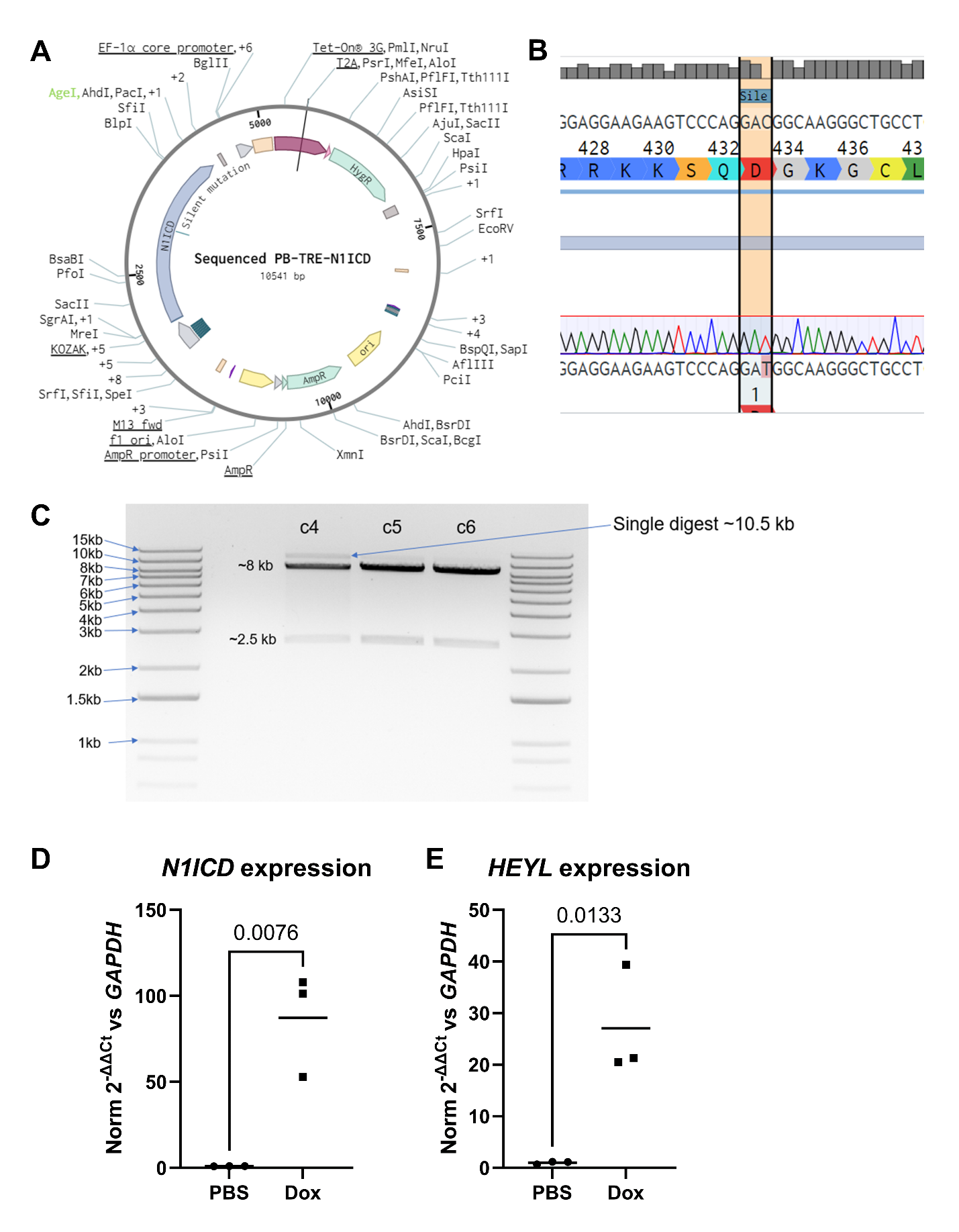


**Figure S6. Generation of a hPSC line with doxycycline-inducible overexpression of *N1ICD***: **(A)** Schematic of the PB-TRE-N1ICD plasmid, which contains the transposon encoding doxycycline-inducible *N1ICD* overexpression elements that was integrated into the hPSC genome by a piggyBac transposase. The transposon is located between 3’ and 5’ piggyBac inverted repeats. A TRE3G doxycycline-inducible promoter is followed by the human native *N1ICD* sequence, similar to the sequence for *N1ICD* in the pWPI-N1ICD plasmid. There is also an EF-1α core promoter followed by a Tet-On 3G gene, followed by a hygromycin resistance cassette (HygR). Outside of the piggyBac inverted repeats is an ampicillin resistance gene that can be used for selection of transformed *E. coli* clones during plasmid amplification. **(B)** Screenshot of results of Sanger sequencing of cloned PB-TRE-N1ICD plasmid showing a silent mutation (GAC 🡪 GAT) in coding sequence of *N1ICD* for amino acid 433 (aspartic acid). **(C)** Gel electrophoresis image showing purified PB-TRE-N1ICD from 3 separate clones (c4, c5, c6), digested at both NheI and AgeI restriction sites. Ladder band sizes are shown on the right. All 3 clones have bands of expected size for the doxycycline-inducible plasmid backbone (~8 kb) and *N1ICD* insert (~2.5 kb). A band is also seen at ~10.5 kb corresponding to a small fraction of plasmid digested at a single restriction site. Clone c6 was Sanger sequenced (results shown in (A) and (B)). **(D-E)** RT-qPCR analysis of a population of *N1ICD* overexpressing IMR90-4 iPSCs containing heterogeneous copy numbers of the PB-TRE-N1ICD transposon, after 3 days treatment with 1 μg/mL doxycycline or PBS control. Expression of **(D)** *NOTCH1* and **(E)** *HEYL* (gene downstream of Notch signaling activation) are expressed as 2^-ΔΔCt^ normalized to *GAPDH*. Points represent *n* = 3 biological replicates. Horizontal bar indicates mean. Relative gene expression values normalized to *GAPDH* were further normalized within each analysis such that the mean of the PBS condition was equal to 1. Statistical analyses were performed on *GAPDH-*normalized data; *P*-values: Student’s *t*-test.


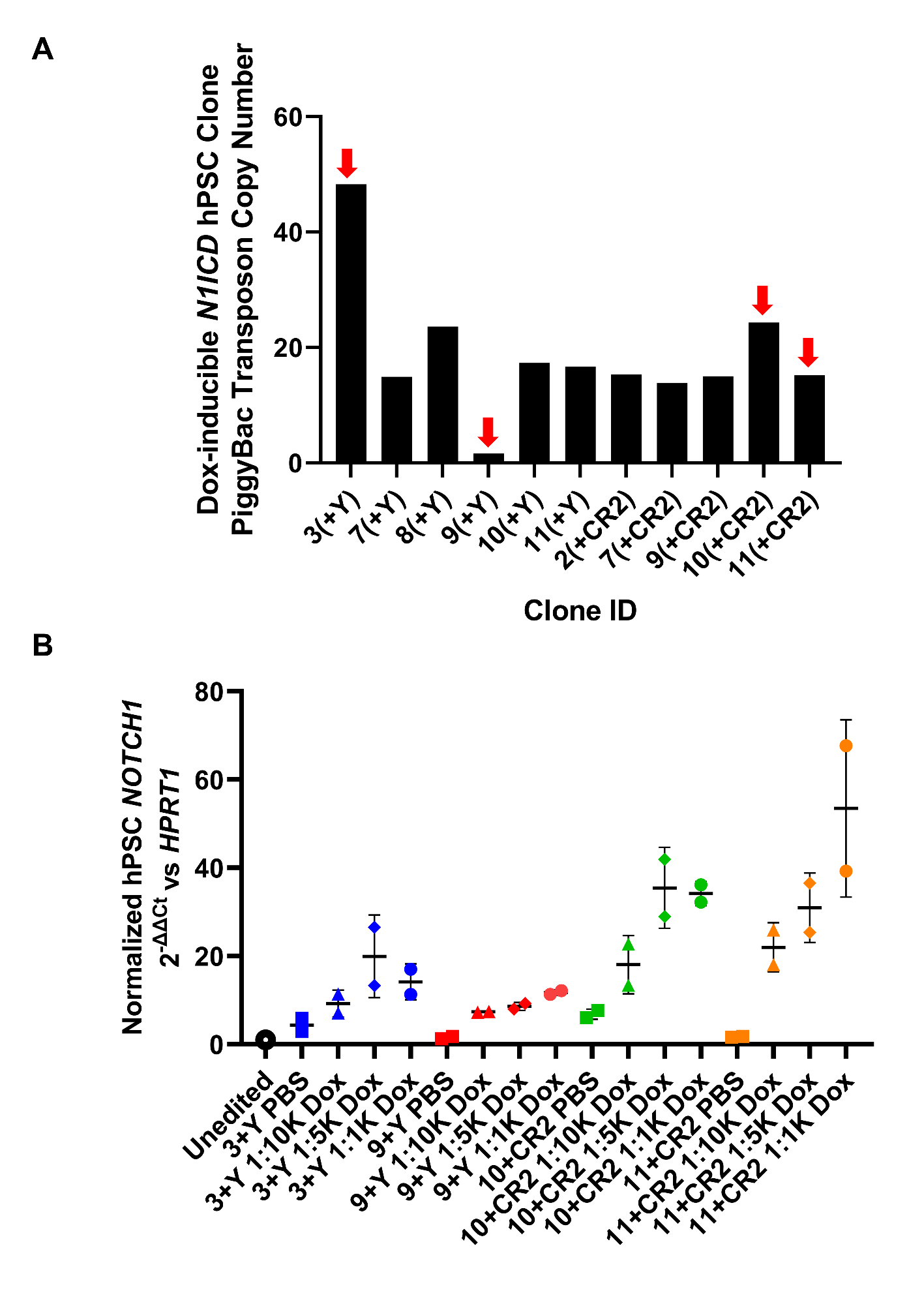


**Figure S7. Characterization of clonal PB-TRE-N1ICD hPSC-CECs**: **(A)** RT-qPCR quantification of piggyBac transposon copy number in 11 clonal populations of *N1ICD* overexpressing IMR90-4 iPSCs containing the PB-TRE-N1ICD transposon. The assay determines the number of piggyBac inserts relative to the 2 *UCR1* loci per genome. PiggyBac insert copy number = 0.5 x (2^-((average piggyBac insert Ct) – (average UCR1 Ct))^). Clone names are indicated on the x-axis; (+Y) or (+CR2) notation indicates whether the edited IMR90-4 iPSC clones were generated in medium supplemented with ROCK inhibitor Y-27632 or CloneR2 respectively. Clones with highly variable copy numbers indicated by red arrows were selected for further characterization. **(B)** RT-qPCR analysis of *N1ICD* expression in PB-TRE-N1ICD hPSC clones 3(+Y) [48.3 copies], 9(+Y) [1.6 copies], 10(+CR2) [24.3 copies], and 11(+CR2) [15.2 copies] treated for 3 days with 1 μg/mL, 200 ng/mL, or 100 ng/mL doxycycline or PBS control, with *n* = 2 biological replicates per condition. An additional unedited hPSC sample (*n* = 1) was included for reference. *N1ICD* expression is expressed as 2^-ΔΔCt^ normalized to *HPRT1*. Bars indicate mean ± SD. Relative gene expression values normalized to *HPRT1* were further normalized to expression in unedited hPSCs such that relative *N1ICD* expression in unedited hPSCs was equal to 1.


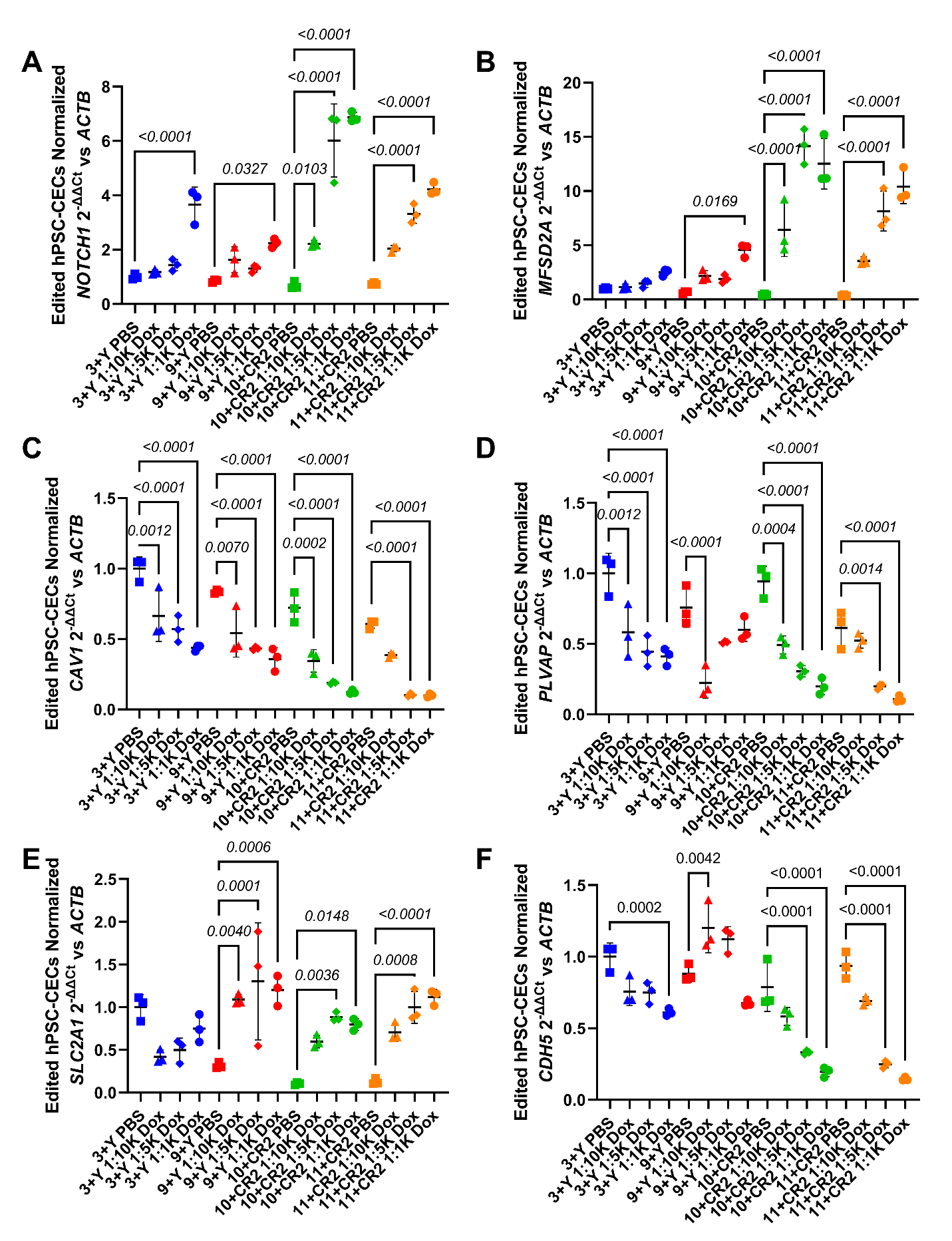


**Figure S8. Blood-brain barrier gene expression in clonal PB-TRE-N1ICD hPSC-CECs as a function of doxycycline concentration.** RT-qPCR analysis of various markers in D15 N1ICD overexpressing clonal hPSC-CECs treated with 4 μM CHIR with or without various concentrations of doxycycline (100 ng/mL [1:10K], 200 ng/mL [1:5K], or 1 ug/mL [1:1K]) or 1:1000 diluted PBS. Relative expression (2^-ΔΔCt^ normalized to ACTB) of **(A)** NOTCH1, **(B)** MFSD2A, **(C)** CAV1, **(D)** PLVAP, **(E)** SLC2A1, and **(F)** CDH5 in CECs derived from edited hPSC clones 3(+Y), 9(+Y), 10(+CR2), and 11(+CR2). In all analyses, points represent n = 3 biological replicates from one differentiation of IMR90-4 PB-TRE-N1ICD 10+CR2 hPSC-derived CECs. Bars indicate mean ± SD. Relative gene expression values normalized to ACTB were further normalized within each analysis for (A)-(F) such that the mean of the 3(+Y) 1:1K PBS condition was equal to 1. Statistical analyses were performed on normalized data; P-values: One-way ANOVA with post-hoc Tukey’s HSD test.


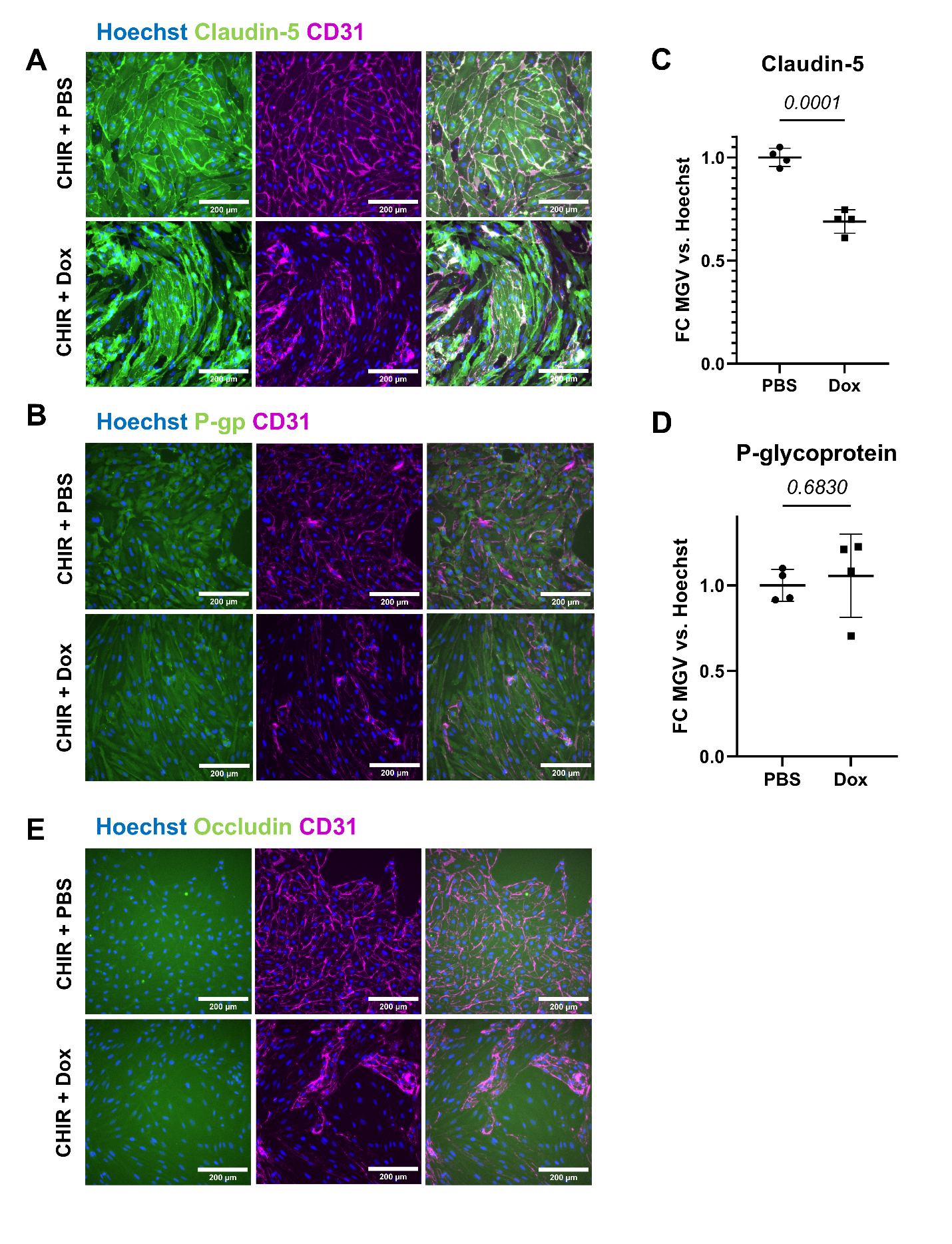


**Figure S9. Immunostaining of PB-TRE-N1ICD 10(+CR2) hPSC-CECs with doxycycline-inducible N1ICD overexpression, continued:** Immunocytochemistry (ICC) analysis of BBB markers in PB-TRE-N1ICD hPSC-CECs treated with CHIR and 1:1000 diluted PBS or CHIR and 1 μg/mL doxycycline on D15 after 10 days of culture in hECSR supplemented with CHIR from D5 to D11 and 4 days of CHIR and doxycycline or PBS treatment from D11 to D15. **(A)** Representative images of ICC analysis for claudin-5 and CD31. Hoechst nuclear counterstain is overlaid in all images. Scale bar: 200 μm. **(B)** Quantification of claudin-5 mean gray value (MGV) in different conditions from (A), normalized to Hoechst MGV. **(C)** Representative images of ICC analysis for P-glycoprotein (P-gp) and CD31. Hoechst nuclear counterstain is overlaid in all images. Scale bar: 200 μm. **(D)** Quantification of P-gp MGV in different conditions from (C), normalized to Hoechst MGV. In (B) and (D), points represent n = 4 biological replicates from one differentiation of IMR90-4 PB-TRE-N1ICD 10(+CR2) hPSC-derived CECs. Horizontal bars indicate mean ± SD. Hoechst-normalized relative fluorescence for each of the three markers was further normalized within each analysis such that the mean of the PBS condition was equal to 1. **(E)** Representative images of ICC analysis for occludin and CD31. Hoechst nuclear counterstain is overlaid in all images. Scale bar: 200 μm. Statistical analyses were performed on Hoechst-normalized data; P-values: Student’s t test.


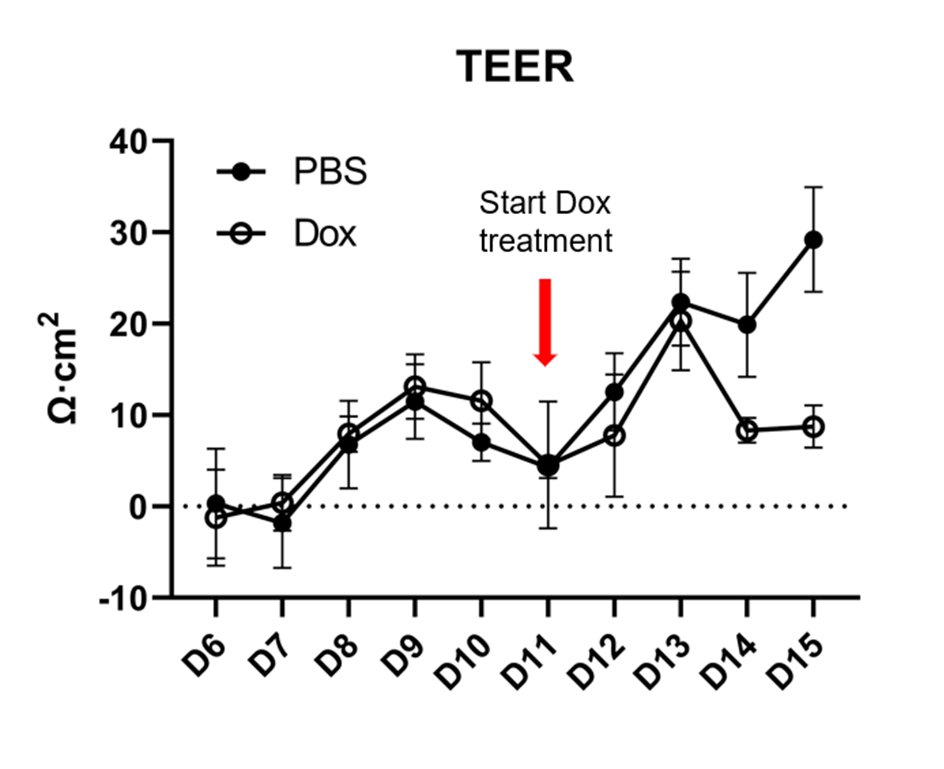


**Figure S10. TEER measurement in PB-TRE-N1ICD 10(+CR2) hPSC-CECs ± Dox:** Transendothelial electrical resistance (TEER) of *N1ICD* overexpressing hPSC-CECs cultured in hECSR supplemented with CHIR, treated with or without 1 μg/mL doxycycline from D11-D15. X axis indicates the day in the differentiation based on the schematic shown in Figure 4A. Points represent *n* = 4 biological replicates from one independent differentiation of IMR90-4 PB-TRE-N1ICD hPSC-CECs. Horizontal bars indicate standard deviation of means of TEER at each time point.


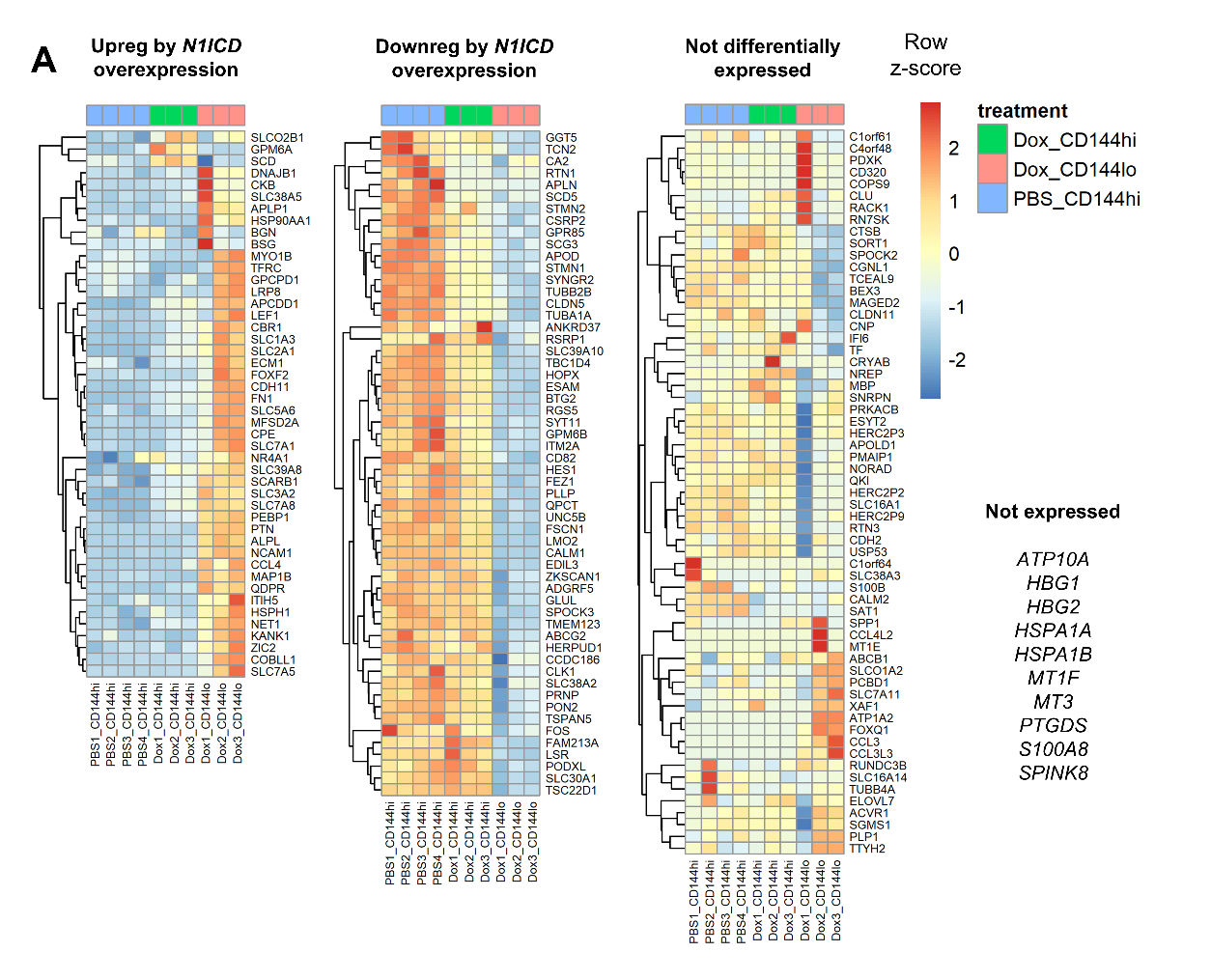

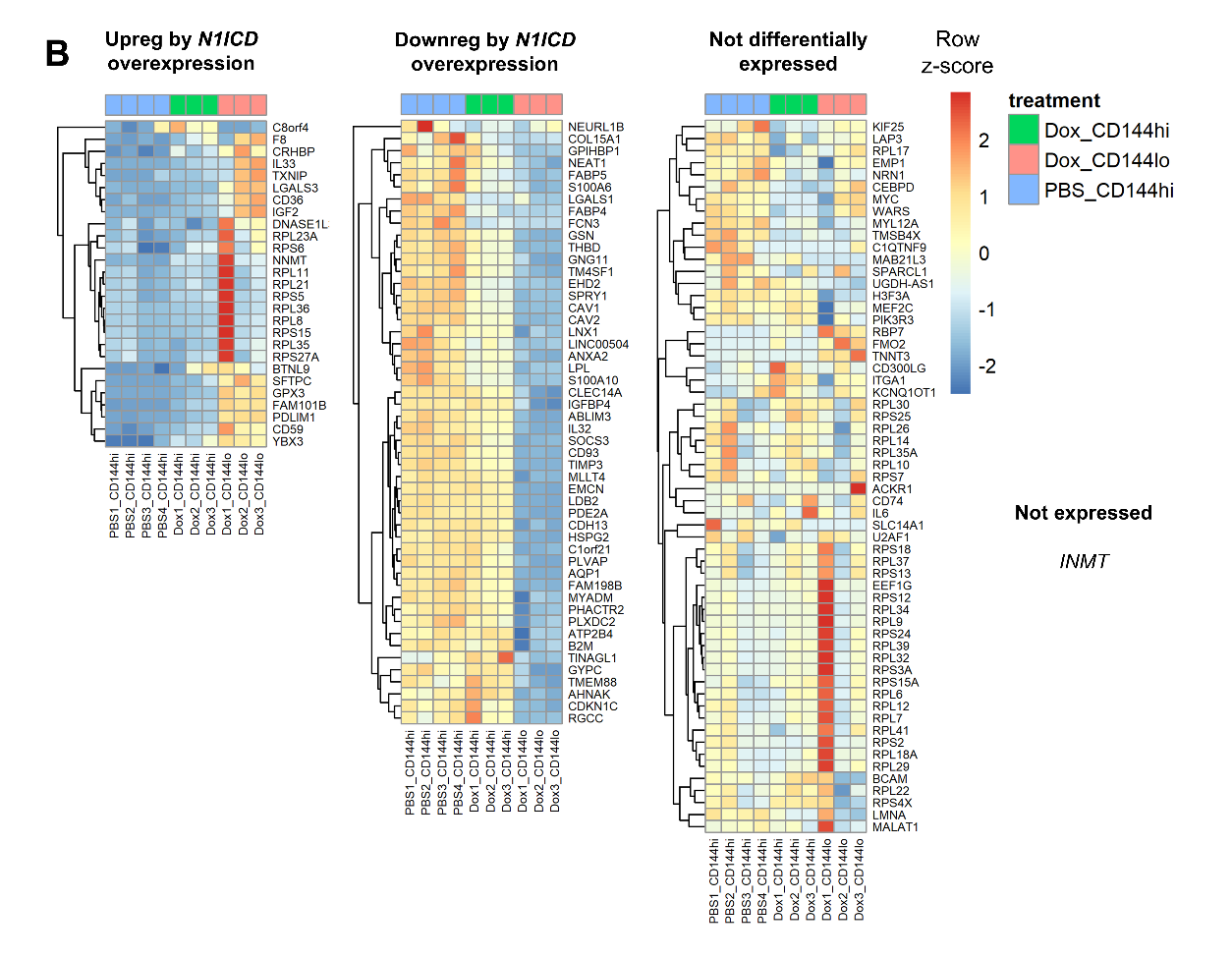


**Figure S11. Differential expression of genes expected up- and down-regulated at the blood-brain barrier in PB-TRE-N1ICD 10(+CR2) hPSC-CECs ± Dox:** Endothelial cells were isolated using Seurat from an integrated single cell transcriptomic analysis [22] of multiple organs, including brain vasculature and peripheral organ vascular beds from lung, liver, heart and skeletal muscle. From this, 310 genes were identified to be significantly differentially expressed (absolute value [log (fold change)] >1, false discovery rate < 0.05) between brain endothelial cells and peripheral organ endothelial cells. 173 genes were found to be upregulated and 137 downregulated in brain compared to peripheral endothelium. Heatmaps show relative expression of the 310 genes between the three subpopulations in the PBS- and doxycycline-treated PB-TRE-N1ICD 10(+CR2) hPSC-CECs (PBS-treated, CD144hi; Dox-treated, CD144hi; Dox-treated, CD144lo). **(A)** Of the 173 genes expected to be upregulated in brain endothelial cells, 46 are upregulated in response to *N1ICD* overexpression (highest in Dox_CD144lo subpopulation). 56/173 genes are downregulated in response to *N1ICD* overexpression, 61/173 are not differentially expressed and 10 are not expressed (TPM = 0). **(B)** Of the 137 genes expected to be downregulated in brain endothelial cells, 50 are downregulated in response to *N1ICD* overexpression (lowest in Dox_CD144lo subpopulation). 27/137 genes are upregulated in response to *N1ICD* overexpression, 59/137 are not differentially expressed and 1 is not expressed (TPM = 0). Heatmap columns indicate sample/replicate and rows indicate genes. Cell color indicates row z-score normalized gene expression level, ranging from blue (lowest expression) to red (highest). Heatmaps were generated using the pheatmap package in R.


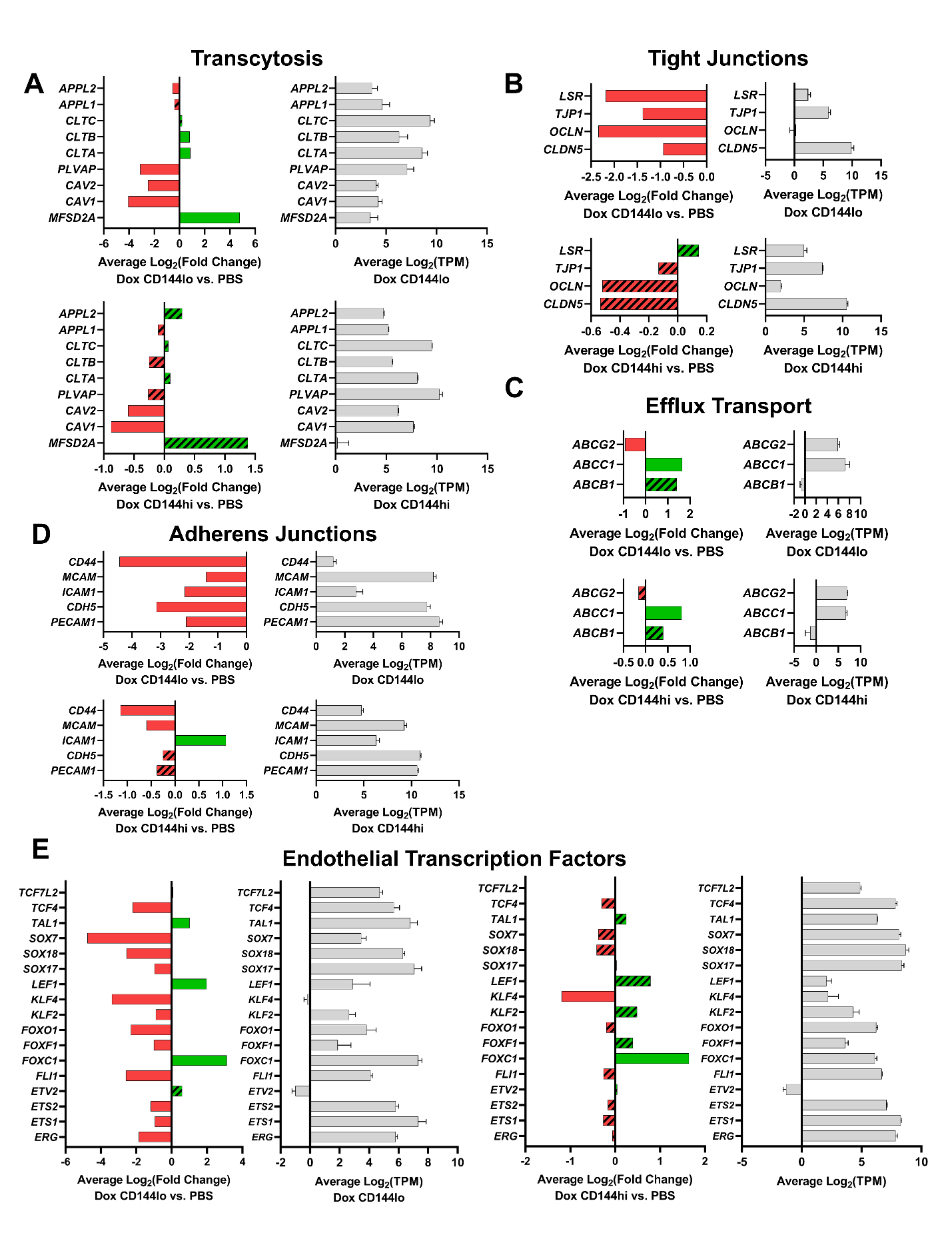


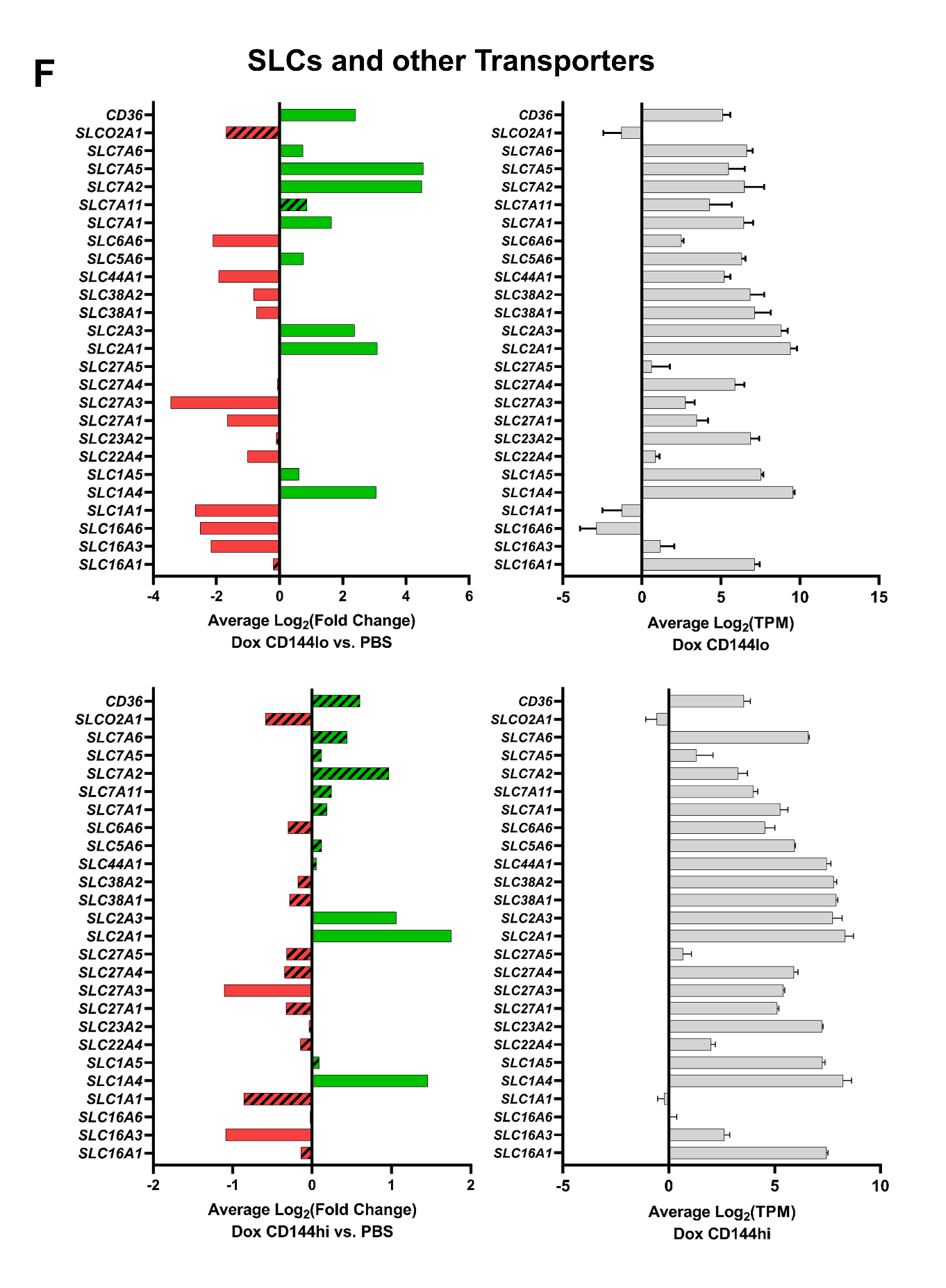


**Figure S12. Relative expression of selected blood-brain barrier related genes in PBS- and Dox-treated subpopulations of PB-TRE-N1ICD 10(+CR2) hPSC-CECs:** Average log_2_(fold change) in Dox-treated subpopulation (CD144hi or CD144lo) relative to PBS control and average log_2_(TPM) for Dox-treated cells from CD144hi or CD144lo subpopulations of various genes related to blood-brain barrier and general endothelial cell properties. Genes in each category were selected based on literature, including a list used in a recent publication by Porkoláb et al. [61]. The categories of genes listed include **(A)** Transcytosis-related, **(B)** tight junction-related, **(C)** efflux transport-related, **(D)** adherens junction-related, **(E)** endothelial transcription factor-related, and **(F)** SLC cassette nutrient transporter-related. Values for average log_2_(fold change) in Dox vs. PBS-treated cells were obtained from DESeq2 analysis. Green and red bars denote positive and negative fold-change, respectively. Shading indicates that the fold change is not statistically significant (FDR ≥ 0.05). In graphs of normalized gene expression (average log_2_[TPM]), vertical bars indicate standard deviation between replicates for Dox-treated cells. All graphs generated using GraphPad Prism.


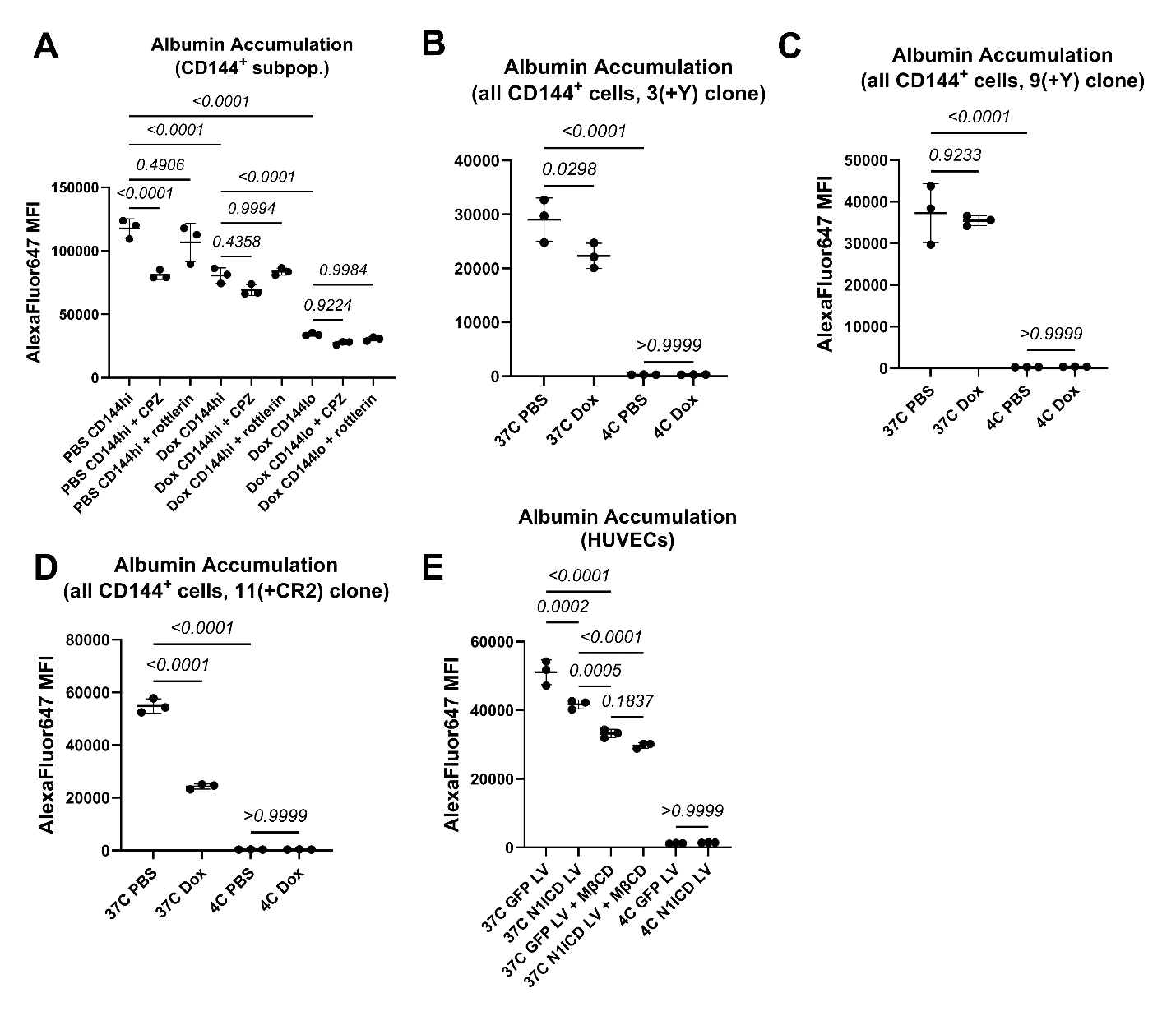


**Figure S13. *N1ICD* overexpression in PB-TRE-N1ICD 10(+CR2) hPSC*-*CECs and primary endothelial cells reduces fluorescent albumin accumulation by inhibiting caveolae- and clathrin-mediated endocytic pathways:** Quantification of flow cytometry-based measurement of endocytic uptake of bovine serum albumin, or BSA, conjugated to AlexaFluor647 (albumin-AF647) into **(A-D)** PB-TRE-N1ICD hPSC-CECs treated with doxycycline or PBS or **(E)** human umbilical vein endothelial cells (HUVECs) treated with *N1ICD* overexpressing lentivirus or control *GFP* lentivirus. **(A)** Quantification of albumin-AF647 accumulation in gated subpopulations for 10(+CR2) edited hPSC-CECs incubated at 37°C, both with and without chlorpromazine (CPZ, clathrin-mediated endocytosis inhibitor) or rottlerin (macropinocytosis inhibitor) pretreatment. Selected statistical comparisons shown between accumulation for each subpopulation and its respective CPZ- or rottlerin-pretreated condition as well as between individual subpopulations in the absence of CPZ or rottlerin pretreatment. **(B)** Quantification of albumin-AF647 accumulation in all CD144-positive cells from each condition for 3(+Y) edited hPSC-CECs. Conditions incubated at 37°C as well as 4°C control were included. Selected statistical comparisons are shown among PBS- or Dox-treated conditions at both temperatures. **(C)** Quantification of albumin-AF647 accumulation in all CD144-positive cells from each condition for 9(+Y) edited hPSC-CECs. Conditions incubated at 37°C as well as 4°C control were included. Selected statistical comparisons are shown among PBS- or Dox-treated conditions at both temperatures. **(D)** Quantification of albumin-AF647 accumulation in all CD144-positive cells from each condition for 11(+CR2) edited hPSC-CECs. Conditions incubated at 37°C as well as 4°C control were included. Selected statistical comparisons are shown among PBS- or Dox-treated conditions at both temperatures. **(E)** Quantification of albumin-AF647 accumulation in HUVECs transduced with N1ICD LV or GFP LV, with or without MβCD pretreatment. Conditions incubated at 37°C as well as 4°C control were included. Selected statistical comparisons are shown among GFP LV- or N1ICD LV-transduced conditions in the presence and absence of MβCD-pretreatment. Points in each graph represent *n =* 3 biological replicates from one differentiation of PB-TRE-N1ICD hPSCs or vial of HUVECs, respectively. Horizontal bars indicate mean ± SD. Statistical analyses for all comparisons were performed on raw MFI data; P-values < 0.05 by one-way ANOVA with post-hoc Tukey’s test.

**Table S1. Antibodies.**

| Target | Species/  Isotype | Manufacturer, clone (product number), RRID | Fluorophore | App* | Dilution |
| --- | --- | --- | --- | --- | --- |
| Caveolin-1 | Rabbit polyclonal | Cell Signaling Technology (3238), RRID: AB_ 2072166 | Unconjugated | ICC | 1:500 |
| Caveolin-1 | Rabbit monoclonal | Cell Signaling Technology (3267)  RRID: AB_2275453 | Unconjugated | TEM | 1:100 |
| Caveolin-1 | Rat monoclonal | Miltenyi Biotec REAL686 (130-129-282), RRID: AB_2904649 | PE | FC | 1:50 |
| CD31 (PECAM-1) | Rabbit polyclonal | Lab Vision (RB-10333-P),  RRID: AB_720502 | Unconjugated | ICC | 1:100 |
| CD31 (PECAM-1) | Mouse IgG1 | Miltenyi Biotec AC128 (130-119-891), RRID: AB_2784124 | APC | FC | 1:50 |
| CD34 | Mouse IgG2a | Miltenyi Biotec AC136 (130-113-178), RRID: AB_2726005 | FITC | FC | 1:50 |
| CD144 (VE-cadherin) | Recomb. human IgG1 | Miltenyi Biotec REA199  (130-125-985), RRID: AB_2857821 | APC | FC | 1:50 |
| CD144 (VE-cadherin) | Recomb human IgG1 | Miltenyi Biotec REA199  (130-123-688), RRID: AB_2819510 | FITC | FC | 1:50-1:100 |
| GFP | Mouse IgG2a | Santa Cruz, B-2 (sc-9996),  RRID: AB_627695 | Unconjugated | ICC | 1:100 |
| GLUT-1 | Mouse IgG2a | Invitrogen SPM498 (MA5-11315), RRID: AB_10979643 | Unconjugated | ICC | 1:200 |
| Isotype control | Mouse IgG1 | R&D Systems 11711 (IC002A),  RRID: AB_357239 | APC | FC | 1:50 |
| Isotype control | Mouse IgG1, κ | BioLegend MOPC-21 (400108), RRID: AB_326429 | FITC | FC | 1:50 |
| Notch1 | Rabbit IgG | Cell Signaling Technology, D1E11 (3608), RRID: AB_2153354 | Unconjugated | WB | 1:500 |
| PLVAP | Rabbit polyclonal | Prestige Antibodies (HPA002279), RRID: AB_1079636 | Unconjugated | ICC | 1:200 |
| β-actin | Rabbit IgG | Cell Signaling Technology, 13E5 (4970), RRID: AB_2223172 | Unconjugated | WB | 1:1000 |
| Rabbit IgG | Goat polyclonal | LI-COR, (925-58071),  RRID: AB_10956166 | IRDye 680RD | WB | 1:5000 |
| Mouse IgG2a | Goat polyclonal | Invitrogen (A-21131),  RRID: AB_141618 | Alexa Fluor 488 | ICC | 1:200 |
| Rabbit IgG | Goat polyclonal | Invitrogen (A-11008),  RRID: AB_143165 | Alexa Fluor 488 | ICC | 1:200 |
| Mouse IgG | Goat polyclonal | Invitrogen (A-21422),  RRID: AB_141822 | Alexa Fluor 555 | ICC | 1:200 |
| Rabbit IgG | Goat polyclonal | Invitrogen (A-11012),  RRID: AB_2534079 | Alexa Fluor 594 | ICC | 1:200 |
| Mouse IgG2a | Goat polyclonal | Invitrogen (A-21135),  RRID: AB_2535774 | Alexa Fluor 594 | ICC | 1:200 |
| Rabbit IgG | Goat polyclonal | Invitrogen (A- 21245),  RRID: AB_2535813 | Alexa Fluor 647 | ICC | 1:200 |
| Mouse IgG1 | Goat polyclonal | Invitrogen (A-21240),  RRID: AB_141658 | Alexa Fluor 647 | ICC | 1:200 |
| Mouse IgG | Goat polyclonal | Invitrogen (A-21235),  RRID: AB_2535804 | Alexa Fluor 647 | ICC | 1:200 |
| Rabbit IgG | Goat polyclonal | Nanoprobes (#2003),  RRID: AB_2687591 | Nanogold® | TEM | 1:50 |

*Application: FC, flow cytometry; ICC, immunocytochemistry; WB, western blotting; TEM, transmission electron microscopy

**Table S2. Primer sequences.**

| Gene | Primer sequence (5’ - 3’) |
| --- | --- |
| Primers for cloning | |
| *NOTCH1* forward | TAA GCA TTA ATT AAG CCA CCA TGG TGC TGC TGT CCC GCA AGC G |
| *NOTCH1* reverse | TGC TTA TTA ATT AAT TAC TTG AAG GCC TCC GGA A |
| EF-1⍺ promoter forward | TCA AGC CTC AGA CAG TGG TTC |
| IRES reverse | CCT CAC ATT GCC AAA AGA CG |
| NheI overhang for *N1ICD* (SB001) | ACT AAA GCT AGC TGT CGT GAG GAA TTT CGA CAT TT |
| AgeI overhang for *N1ICD* (SB002) | ACT AAA ACC GGT CCC CCT TTT CTT TTA AAA GTT AAC CG |
| Primers for Sanger sequencing | |
| TRE3G promoter forward (BD69) | GTA CGG TGG GCG CCT ATA AA |
| *N1ICD* reverse (SB003) | GGG CAC CGT CTG AAG CGT TCT T |
| *N1ICD* forward (SB004) | GCA GTG GAC TCA GCA GCA CCT G |
| *N1ICD* reverse (SB005) | GCG GCC CAT GTT GTC CTG GAT G |
| *N1ICD* forward (SB006) | GAG GGC ATG CTG GAG GAC CTC A |
| *N1ICD* forward (SB007) | GGA AGC AAG GAG GCC AAG GAC C |
| *N1ICD* reverse (SB008) | GTG AAA TTC AGG GCC CCT CCG C |
| *N1ICD* forward (SB009) | CCC TGC AGC ATG GCA TGG TAG G |
| *N1ICD* reverse (SB010) | GGG GCT CTC CTG GGG CAG AAT A |
| PB-TRE backbone reverse (BD70) | GGG TAT CGA CAG AGT GCC AG |
| Primers for RT-qPCR | |
| *ABCB1* Taqman probe | Hs00184500_m1 |
| *MFSD2A* Taqman probe | Hs00293017_m1 |
| *ABCC1* forward | CTG AGT TCC TGC GTA CCT ATG |
| *ABCC1* reverse | TGC CAT TCT CCA TTT GCT TTG |
| *ABCG2* forward | CTC AGA TCA TTG TCA CAG TCG T |
| *ABCG2* reverse | GTC GTC AGG AAG AAG AGA ACC |
| *ACTB* forward | ACA GAG CCT CGC CTT TG |
| *ACTB* reverse | CCT TGC ACA TGC CGG AG |
| *CAV1* forward | CAT GGC AGA CGA GCT GAG |
| *CAV1* reverse | AAA CTG TGT GTC CCT TCT GG |
| *CDH2* forward | CTC CAA TCA ACT TGC CAG AA |
| *CDH2* reverse | ATA CCA GTT GGA GGC TGG TC |
| *CDH5* forward | GAA CCA GAT GCA CAT TGA TGA AG |
| *CDH5* reverse | TGC CCA CAT ATT CTC CTT TGA G |
| *CLDN5* forward | TGA CCT TCT CCT GCC ACT A |
| *CLDN5* reverse | AAG CGA AAT CCT CAG TCT GAC |
| *HEYL* forward | CAC TTG AAA ATG CTC CAT GCC |
| *HEYL* reverse | ACT CCC GAA AAC CAA TGC TC |
| *HPRT1* forward | TTG TTG TAG GAT ATG CCC TTG A |
| *HPRT1* reverse | GCG ATG TCA ATA GGA CTC CAG |
| *FN1* forward | TGG TGT CAC AGA GGC TAC TA |
| *FN1* reverse | GGG CTC GCT CTT CTG ATT ATT |
| *GAPDH* forward | ACA TCG CTC AGA CAC CAT G |
| *GAPDH* reverse | TGT AGT TGA GGT CAA TGA AGG G |
| *GFP* forward (SB012) | TCG CCG ACC ACT ACC AGC AGA A |
| *GFP* reverse (SB013) | CGC GCT TCT CGT TGG GGT CTT T |
| *N1ICD* forward (SB004) | GCA GTG GAC TCA GCA GCA CCT G |
| *N1ICD* reverse (SB011) | CTG CAG GAG GCG ATC ATG AGC G |
| *OCLN* forward | ATG GCA AAG TGA ATG ACA AGC |
| *OCLN* reverse | AGG CGA AGT TAA TGG AAG CTC |
| *PLVAP* forward | TGG ACA CCT GCA TCA AGA C |
| *PLVAP* reverse | GGA TCT TCC TCT TGA ACT CCT C |
| *SLC2A1* forward | GTG CCA TAC TCA TGA CCA TCG |
| *SLC2A1* reverse | GGC CAC AAA GCC AAA GAT G |
| *TJP1* forward | CGC GTC TCT CCA CAT ACA TTC |
| *TJP1* reverse | GCT GGC TTA TTC TGA GAT GGA |
